# Asymmetric organocatalytic decarboxylative Mannich reaction using β-keto acids: A new protocol for the synthesis of chiral β-amino ketones

**DOI:** 10.3762/bjoc.8.144

**Published:** 2012-08-13

**Authors:** Chunhui Jiang, Fangrui Zhong, Yixin Lu

**Affiliations:** 1 Department of Chemistry & Medicinal Chemistry Program, Life Sciences Institute, National University of Singapore, 3 Science Drive 3, Republic of Singapore, 117543

**Keywords:** decarboxylative addition, β-keto acid, Mannich reaction, organocatalysis

## Abstract

The first decarboxylative Mannich reaction employing β-keto acids, catalyzed by cinchonine-derived bifunctional thiourea catalyst has been described. The desired β-amino ketones were obtained in excellent yields and with moderate to good enantioselectivities.

## Introduction

Chiral β-amino ketones are an important class of building blocks for the synthesis of 1,3-amino alcohols [1–2], 1,3-amino acids [3] and other bioactive nature products [4–6]. Given their synthetic significance, methods for the asymmetric synthesis of β-amino ketones have been extensively investigated over the past few decades [7]. Among them, the Mukaiyama–Mannich reaction performed with silyl enol ethers and sulfonyl aldimines, catalyzed by a chiral Lewis acid complex, is one of the most important synthetic methods [8–13]. Apparently, direct use of inactivated ketones as a donor would be of great practical value. Indeed, direct approaches such as asymmetric enamine catalysis [14–17] and Brønsted acid catalysis [18] have been reported, through the activation of ketones or aryl imines [19]. However, substrates for the enamine activation are limited to only acetone and cyclic alkyl ketones. Application of aryl methyl ketones in the asymmetric Mannich reaction by enamine catalysis remains elusive. On the other hand, the only chiral Brønsted acid catalytic system based on BINOL-phosphates was reported by Rueping et al. Unfortunately, the yields of the reported reactions were unsatisfactory and the enantioselectivities were modest [20].

In recent years, inspired by the enzymatic synthesis of polyketides and fatty acids in biological systems, the enantioselective decarboxylative reactions of malonic acid half thioesters (MAHTs) have received much attention. In this regard, various electrophiles, including aldehydes, ketones, imines, activated alkenes and azodicarboxylates, have been employed as electrophiles in the presence of metal [21–25] or organocatalysts [26–36].

**Scheme 1:**
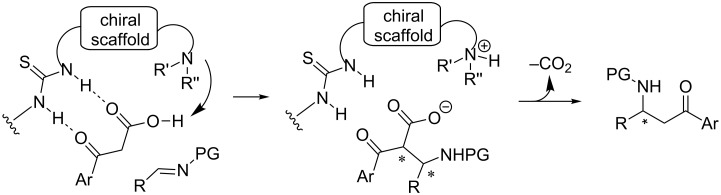
Working hypothesis: Decarboxylative Mannich reaction.

To provide a practical solution to the low reactivity associated with aryl methyl ketones, we wondered whether β-keto acids could serve as an enolate equivalent to aryl methyl ketones upon decarboxylation (Scheme 1). The proposed addition–decarboxylation sequence is consistent with current mechanistic understanding [31,35,37–38]. However, we cannot exclude an alternative decarboxylation–addition pathway at this stage. In fact, in sharp contrast to the popular use of malonic acid half thioesters (MAHTs) as an ester enolate equivalent in enantioselective decarboxylative additions [39], the employment of β-keto acids as a reaction partner in decarboxylative processes has rarely been explored [37]. Herein, we reported the first decarboxylative Mannich reaction between the β-keto acids and sulfonylimines, affording chiral β-amino ketones in excellent yields and good enantioselectivities.

## Findings

In our initial screening, we examined the model reaction between tosylimine **1a** and β-keto acid **2a** in the presence of a range of bifunctional catalysts (Table 1). We first evaluated the catalytic effects of several cinchona alkaloid derivatives. Commercially available cinchonidine (**CD-1**) led to the formation of the product with disappointing enantioselectivity (Table 1, entry 1). Quinine-derived sulfonamide [40], β-isocupreidine **(β-ICD**) [41–42] and biscinchona alkaloid (DHQ)_2_AQN were all found to be poor catalysts (Table 1, entries 2–4). On the other hand, cinchona alkaloid derived bifunctional thiourea tertiary amine catalysts afforded much improved results (Table 1, entries 5–7). Among them, the cinchonine based thiourea **C-1** turned out to be the best catalyst, and the Mannich product was isolated with 58% ee (Table 1, entry 7). In addition, we also examined several other bifunctional catalysts based on amino acids [43–44], including threonine derived **Thr-1** [45], and tryptophan based **Trp-1** [46], as well as threonine incorporated multifunctional catalyst **CD-3** [47]. However, no further improvement could be achieved (Table 1, entries 8–10). The influence of different imines on the reaction was subsequently explored, and it was found that the electronic nature of the sulfonyl protective groups affected the enantioselectivity. While the employment of nosylimine **1b** led to decreased enantioselectivity (Table 1, entry 11), replacement of tosylimine **1a** with *N*-(*p*-methoxybenzenesulfonyl)imine **1c** resulted in further improvement, and the product was obtained in 65% ee (Table 1, entry 12). However, when ethoxycarbonylimine **1d** was used, nearly racemic products were obtained, suggesting the importance of the sulfonyl group in the asymmetric induction (Table 1, entry 13). Less reactive imines, such as diphenylphosphinoylimine **1e** and Cbz-imine **1f**, proved to be unsuitable for the reaction (Table 1, entries 14 and 15).

**Table 1:** Exploration of the decarboxylative addition of β-ketoacids to imines.

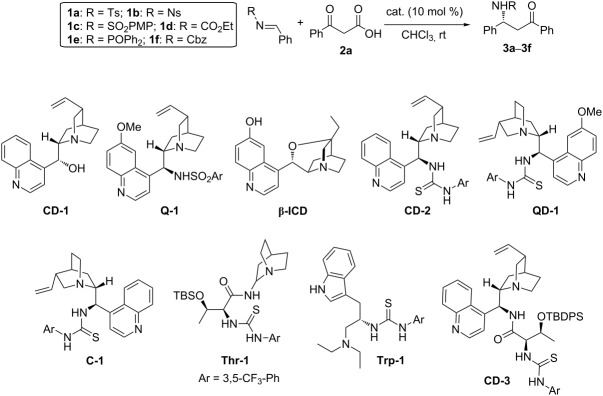				

Entry^a^	**1**	cat	Yield (%)^b^	ee (%)^c^

1	**1a**	**CD-1**	86	17
2	**1a**	**Q-1**	54	13
3	**1a**	(DHQ)_2_AQN	90	2
4	**1a**	**β-ICD**	93	27
5	**1a**	**CD-2**	97	44
6	**1a**	**QD-1**	93	54
7	**1a**	**C-1**	95	58
8	**1a**	**Thr-1**	88	12
9	**1a**	**Trp-1**	95	21
10	**1a**	**CD-3**	92	13
11	**1b**	**C-1**	95	49
12	**1c**	**C-1**	96	65
13	**1d**	**C-1**	91	5
14	**1e**	**C-1**	trace	–
15	**1f**	**C-1**	trace	–

^a^Reactions were performed with **1** (0.05 mmol), **2a** (0.075 mmol) and the catalyst (0.005 mmol) in CHCl_3_ (0.5 mL). ^b^Isolated yield. ^c^Determined by HPLC analysis on a chiral stationary phase.

A screening of the solvent effect was then followed, and the results are summarized in Table 2. In general, the reaction proceeded very well in common aprotic solvents, and excellent yields were consistently obtained (Table 2, entries 1–9). Enantioselectivity of the reaction varied, and diethyl ether was found to be the best solvent, furnishing the desired product with 72% ee. Employment of other etheric solvents, including methyl *tert*-butyl ether and dioxane, and lowering reaction temperature did not offer further improvement (Table 2, entries 10–12).

**Table 2:** Solvent screening.

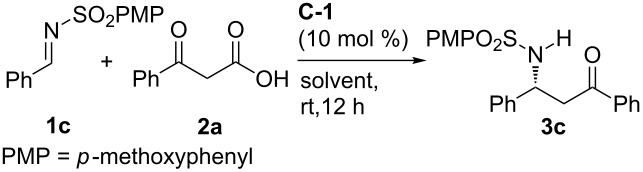			

Entry^a^	Solvent	Yield (%)^b^	ee (%)^c^

1	CHCl_3_	96	65
2	THF	93	64
3	DCM	92	66
4	toluene	90	63
5	**diethyl ether**	**93**	**72**
6	ethyl acetate	92	66
7	benzene	90	67
8	DCE	91	66
9	acetone	92	52
10	methyl *tert*-butyl ether	92	62
11	dioxane	94	65
12^d^	diethyl ether	67	65

^a^Reactions were performed with **1c** (0.05 mmol), **2a** (0.075 mmol) and **C-1** (0.005 mmol) in the solvent specified (0.5 mL). ^b^Isolated yield. ^c^Determined by HPLC analysis on a chiral stationary phase. ^d^Reaction was performed at 0 °C.

To establish the substrate scope, a number of sulfonylimines derived from aromatic aldehydes were employed as acceptors, and the results are summarized in Table 3. In general, the reaction worked well for imines with various substituents at different positions of the phenyl ring, including electron-withdrawing groups, electron-donating groups and halogen atoms, and excellent yields and moderate ee values were obtained (Table 3, entries 1–10). Heterocycles were well-tolerated, and good enantioselectivities were obtained with 2-furyl and thiophen-2-yl containing substrates (Table 3, entries 11 and 12). The aryl groups of β-keto acids could also be varied, and the reaction was applicable to β-keto acids with different aromatic substituents (Table 3, entries 13–17). Furthermore, the reaction was also applicable to alkyl β-keto acids, and comparable chemical yields and enantioselectivities were attainable (Table 3, entries 18–19). The absolute configurations of the products were assigned by comparing the optical rotation of **3a** with the value reported in the literature [48] (see the Supporting Information File 1 for details).

**Table 3:** Substrate scope.

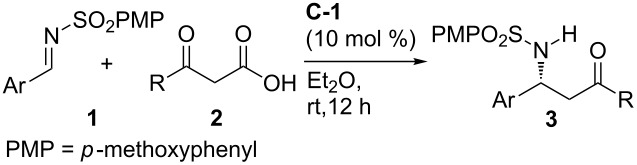					

Entry^a^	Ar	R	**3**	Yield (%)^b^	ee (%)^c^

1	Ph	Ph	**3c**	93	72
2	4-Me-C_6_H_4_	Ph	**3g**	90	64
3	4-Br-C_6_H_4_	Ph	**3h**	88	61
4	4-CF_3_-C_6_H_4_	Ph	**3i**	85	55
5	4-OMe-C_6_H_4_	Ph	**3j**	97	62
6	2-F-C_6_H_4_	Ph	**3k**	89	65
7	2-Me-C_6_H_4_	Ph	**3l**	92	65
8	2-Br-C_6_H_4_	Ph	**3m**	87	59
9	3-Me-C_6_H_4_	Ph	**3n**	97	65
10	3-Br-C_6_H_4_	Ph	**3o**	96	61
11	2-furyl	Ph	**3p**	94	83
12	thiophen-2-yl	Ph	**3q**	87	77
13	Ph	4-F-C_6_H_4_	**3r**	95	64
14^d^	Ph	3-Cl-C_6_H_4_	**3s**	62	70
15	Ph	2-naphthyl	**3t**	62	69
16	Ph	4-Me-C_6_H_4_	**3u**	93	67
17	Ph	2-OMe-C_6_H_4_	**3v**	88	60
18	Ph	*n*-Pr	**3w**	92	54
19	Ph	*t*-Bu	**3x**	75	73

^a^Reactions were performed with **1** (0.05 mmol), **2** (0.075 mmol) and **C-1** (0.005 mmol) in Et_2_O (0.5 mL). ^b^Isolated yield. ^c^Determined by HPLC analysis on a chiral stationary phase. ^d^The catalyst loading was 20 mol %.

In conclusion, we have developed the first organocatalytic decarboxylative Mannich reaction employing β-keto acids as the donor. The reaction was effectively catalyzed by cinchonine-based bifunctional catalyst **C-1**, and the synthetically useful β-amino ketones were prepared in excellent yields and with moderate to good enantioselectivities. The method reported represents a new protocol for the asymmetric construction of β-amino ketones.

## Experimental

### General procedure for the decarboxylative Mannich reaction of β-keto acids and aldimines

To a solution of imine **1c** (13.8 mg, 0.05 mmol) and **C-1** (2.8 mg, 0.005 mmol) in ether (0.5 mL) at room temperature, was added β-keto acid **2a** (12.3 mg, 0.075 mmol). The reaction mixture was stirred for 12 h. The solvent was then removed under reduced pressure, and the residue was purified by flash chromatography on silica gel (hexane/ethyl acetate 5:1 to 3:1) to afford **3c** as a white solid (18.4 mg, 93% yield).

## Supporting Information

File 1 Characterization data and spectra of synthesized compounds.
