# Supplementary material for: Asymmetric organocatalytic decarboxylative Mannich reaction using β-keto acids: A new protocol for the synthesis of chiral β-amino ketones
Source: Beilstein J Org Chem. 2012 Aug 13;8:1279–83. doi: 10.3762/bjoc.8.144 (PMC3458749; doi:10.3762/bjoc.8.144)

Supporting Information

for

Asymmetric organocatalytic decarboxylative Mannich reaction using β-keto acids: A new protocol for the synthesis of chiral β-amino ketones

Chunhui Jiang, Fangrui Zhong and Yixin Lu*

Address: Department of Chemistry & Medicinal Chemistry Program, Life Sciences Institute, National University of Singapore, 3 Science Drive 3, Republic of Singapore, 117543

Email: Yixin Lu - chmlyx@nus.edu.sg

* Corresponding author

Characterization data and spectra of synthesized compounds

A. General information S2

B. Representative procedure S3

C. Determination of configurations S3

D. Analytical data and HPLC chromatogram S5

E. NMR spectra S34

A. General information

All the starting materials were obtained from commercial sources and used without further purification unless otherwise stated. THF and diethyl ether were dried and distilled from sodium benzophenone ketyl prior to use. $CHCl_3$ and CH_2Cl_2 were distilled from CaH_2 prior to use. Dioxane was dried and distilled from Na prior to use. All the solvents used in reactions involving phosphorous-containing compounds were degassed by dry N_2. ^{1}H and ^{13}C NMR spectra were recorded on a Bruker ACF300 or AMX500 (500 MHz) spectrometer. Chemical shifts were reported in parts per million (ppm), and the residual solvent peak was used as an internal reference: proton (chloroform δ 7.26), carbon (chloroform δ 77.0). Multiplicity was indicated as follows: s (singlet), d (doublet), t (triplet), q (quartet), m (multiplet), dd (doublet of doublet), br s (broad singlet). Coupling constants were reported in hertz (Hz). Low-resolution mass spectra were obtained on a Finnigan/MAT LCQ spectrometer in ESI mode, and a Finnigan/MAT 95XL-T mass spectrometer in FAB mode. All high-resolution mass spectra were obtained on a Finnigan/MAT 95XL-T spectrometer. For thin layer chromatography (TLC), Merck precoated TLC plates (Merck 60 F_{254}) were used, and compounds were visualized with a UV light at 254 nm. Further visualization was achieved by staining with iodine, or ceric ammonium molybdate followed by heating on a hot plate. Flash chromatographic separations were performed on Merck 60 (0.040–0.063 mm) mesh silica gel. The Enantiomerically excesses of products were determined by chiral-phase HPLC analysis, using a Daicel Chiralcel ID column (250 × 4.6 mm), or Chiralpak OD-H column (250 × 4.6 mm).

The *N*-sulfonylated imines **1** [1-2] and β-keto acids **2** [3] were prepared by following the literature procedure.

B. Representative procedure

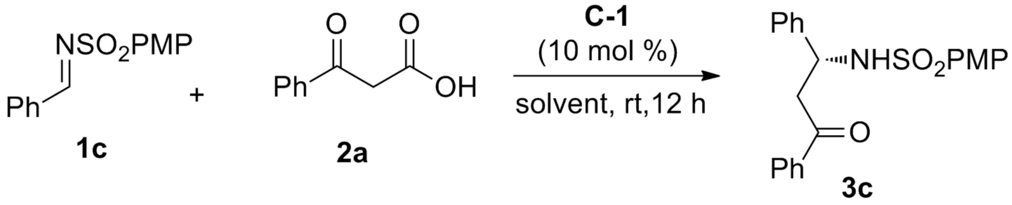

To a solution of imine **1c** (13.8 mg, 0.05 mmol) and **C-1** (2.8 mg, 0.005 mmol) in ether (0.5 mL) at room temperature was added β-keto acid **2a** (12.3 mg, 0.075 mmol). The reaction mixture was stirred for 12 h. The solvent was then removed under reduced pressure, and the residue was purified by flash chromatography on silica gel (hexane/ethyl acetate 5:1 to 3:1) to afford **3c** as a white solid (18.4 mg, 93% yield).

C. Determination of configurations

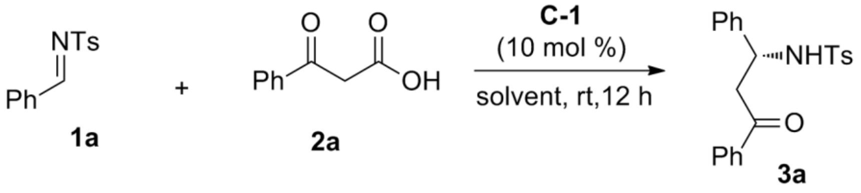

68% ee; $[\alpha]^{25}_{D}$ = +15.8 (*c* 1.0, $CHCl_3$)
ref.: >99% *ee*; $[\alpha]^{25}_{D}$ = –22.0 (*c* 1.0, $CHCl_3$)

Following the representative procedure, compound **3a** was obtained as a white solid (95% yield).

(*R*)-4-Methyl-*N*-(3-oxo-1,3-diphenylpropyl)benzenesulfonamide (**3a**): A white solid; $[\alpha]^{25}_{D}$ = +15.8 (*c* 1.00, $CHCl_3$); ^{1}H NMR (500 MHz, $CDCl_3$) δ 7.80 (d, *J* = 8.3 Hz, 2H), 7.62 (d, *J* = 7.7 Hz, 2H), 7.59–7.50 (m, 1H), 7.41 (t, *J* = 7.5 Hz, 2H), 7.32–7.11 (m, 8H), 5.72 (d, *J* = 6.8 Hz, 1H), 4.85 (dd, *J* = 12.4 Hz, 6.2 Hz, 1H), 3.59 (dd, *J* = 17.4 Hz, 5.4 Hz, 1H), 3.46 (dd, *J* = 17.3 Hz, 6.2 Hz, 1H), 2.36 (s, 3H); HRMS (ESI) *m*/*z*: calcd for $C_{22}H_{21}NO_4S$ $[M + Na]^+$ 418.1074, found 418.1083; The characterization data were in agreement with the literature value.[3] The *ee* value was 68%, t_R (major) = 11.2 min, t_R (minor) = 14.8 min (Chiralcel OD-H,

λ = 254 nm, 30% iPrOH/hexanes, flow rate = 1.0 mL/min).

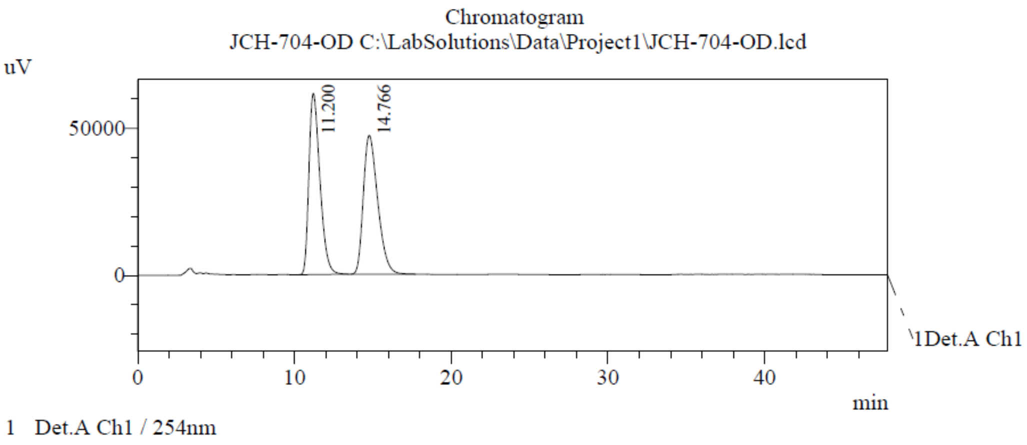

PeakTable

Detector A Ch1 254nm

Peak#	Ret. Time	Area	Height	Area %	Height %
1	11.200	3006108	61626	49.960	56.630
2	14.766	3010949	47195	50.040	43.370
Total		6017057	108820	100.000	100.000

Racemic **3a**

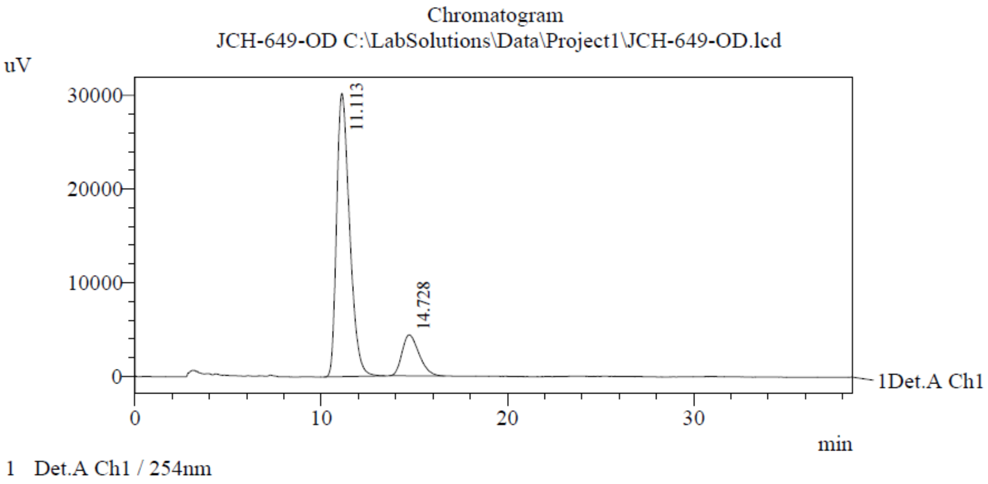

PeakTable

Detector A Ch1 254nm

Peak#	Ret. Time	Area	Height	Area %	Height %
1	11.113	1466534	30226	84.091	87.370
2	14.728	277457	4369	15.909	12.630
Total		1743992	34595	100.000	100.000

Enantiomerically enriched **3a**

The absolute configuration of **3a** was determined to be *R* by comparison with the sign of the optical rotation of the *S* enatiomer reported in the literature [4]. The configurations of other **C-1**-catalyzed decarboxylative Mannich adducts were assigned by analogy.

D. Analytical data and HPLC chromatogram

(*R*)-4-Methoxy-*N*-(3-oxo-1,3-diphenylpropyl)benzenesulfonamide (**3c**)

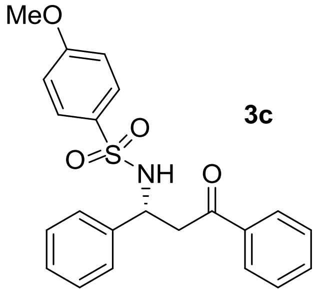

3c

A white solid; $[\alpha]^{25}_{D}$ = +33.8 (*c* 1.00, $CHCl_3$); ^1H NMR (500 MHz, $CDCl_3$) δ 7.81 (dd, *J* = 8.4 Hz, 1.2 Hz, 2H), 7.69–7.63 (m, 2H), 7.59–7.50 (m, 1H), 7.45–7.37 (m, 2H), 7.37–7.12 (m, 6H), 6.85–6.80 (m, 2H), 5.71 (d, *J* = 6.8 Hz, 1H), 4.85 (dd, *J* = 12.5 Hz, 6.2 Hz, 1H), 3.82 (s, 3H), 3.58 (dd, *J* = 17.3 Hz, 5.6 Hz, 1H), 3.47 (dd, *J* = 17.3 Hz, 6.2 Hz, 1H); ^{13}C NMR (126 MHz, $CDCl_3$) δ 197.79, 162.72, 139.90, 136.31, 133.57, 131.78, 129.31, 128.64, 128.55, 128.03, 127.67, 126.71, 113.99, 55.53, 54.43, 44.76; HRMS (ESI) *m*/*z*: calcd for $C_{22}H_{21}NO_4S$ $[M + Na]^+$ 418.1074, found 418.1083; The *ee* value was 73%, t_R (major) = 15.3 min, t_R (minor) = 20.3 min (Chiralcel OD-H, λ = 254 nm, 30% iPrOH/hexanes, flow rate = 1.0 mL/min).

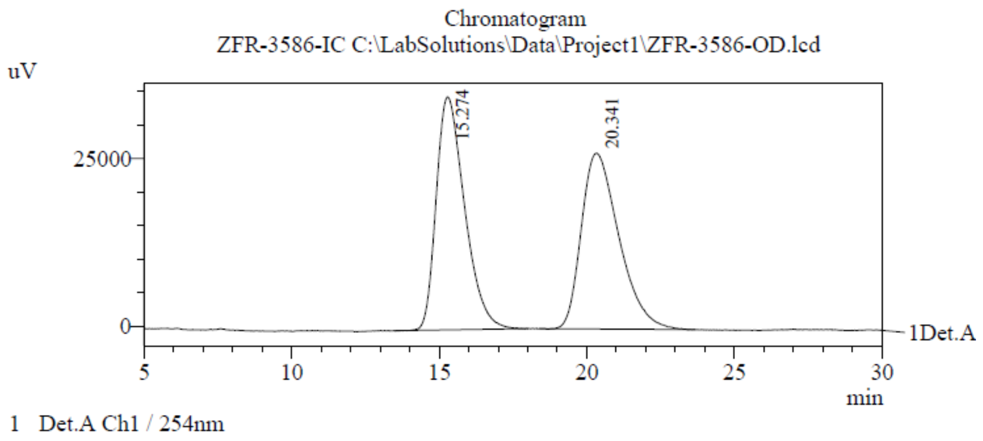

PeakTable

Detector A Ch1 254nm

Peak#	Ret. Time	Area	Height	Area %	Height %
1	15.274	2329310	34745	50.028	56.991
2	20.341	2326701	26221	49.972	43.009
Total		4656011	60966	100.000	100.000

Racemic **3c**

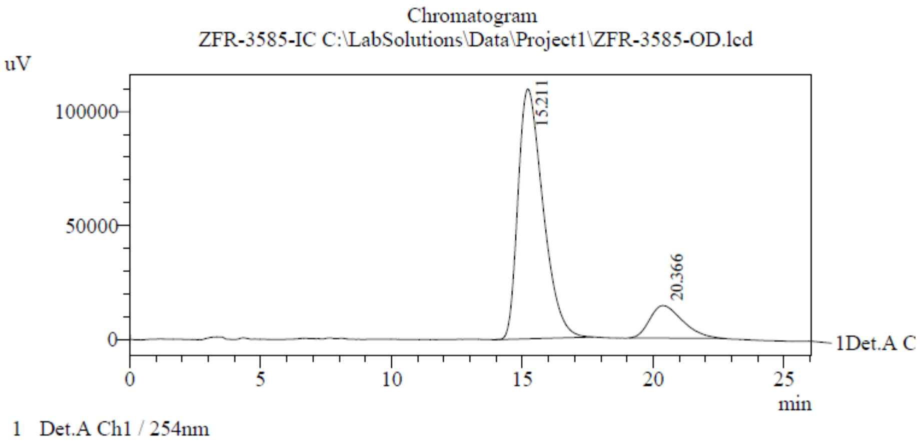

PeakTable

Detector A Ch1 254nm

Peak#	Ret. Time	Area	Height	Area %	Height %
1	15.211	7286622	109520	86.384	88.802
2	20.366	1148526	13810	13.616	11.198
Total		8435148	123330	100.000	100.000

Enantiomerically enriched **3c**

(*R*)-4-Methoxy-*N*-(3-oxo-3-phenyl-1-*p*-tolylpropyl)benzenesulfonamide (**3g**)

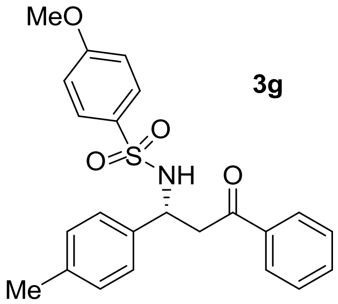

3g

A white solid; $[\alpha]^{25}_{D}$ = +30.4 (*c* 1.00, $CHCl_3$); ^{1}H NMR (500 MHz, $CDCl_3$) δ 7.81 (dd, *J* = 8.3 Hz, 1.1 Hz, 2H), 7.69–7.64 (m, 2H), 7.54 (dd, *J* = 10.6 Hz, 4.3 Hz, 1H), 7.41 (t, *J* = 7.8 Hz, 2H), 7.02 (dd, *J* = 21.9 Hz, 8.1 Hz, 4H), 6.88–6.75 (m, 2H), 5.58 (d, *J* = 6.5 Hz, 1H), 4.79 (q, *J* = 6.2 Hz, 1H), 3.82 (s, 3H), 3.58 (dd, *J* = 17.3 Hz, 5.6 Hz, 1H), 3.46 (dd, *J* = 17.3 Hz, 6.4 Hz, 1H), 2.26 (s, 3H); ^{13}C NMR (126 MHz, $CDCl_3$) δ 197.82, 162.73, 137.39, 136.97, 136.36, 129.34, 129.22, 128.62, 128.04, 126.61, 113.97, 55.52, 54.22, 44.83, 20.98; HRMS (ESI) *m*/*z*: calcd for $C_{23}H_{23}NO_4S$ $[M + Na]^+$ 426.0614, found 426.0634; The *ee* value was 64%, t_R (major) = 11.6 min, t_R (minor) = 15.9 min (Chiralcel OD-H, λ = 254 nm, 30% iPrOH/hexanes, flow rate = 1.0 mL/min).

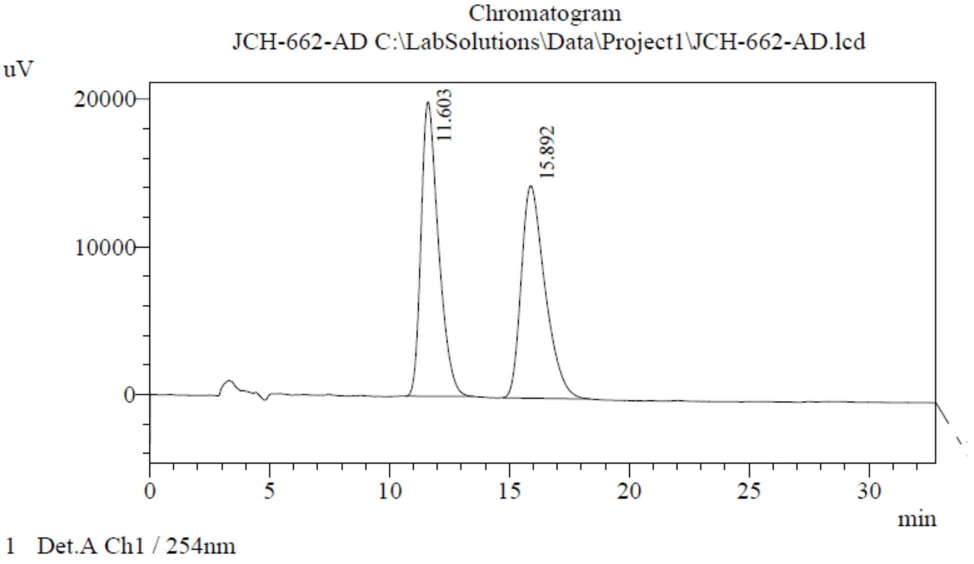

PeakTable

Detector A Ch1 254nm

Peak#	Ret. Time	Area	Height	Area %	Height %
1	11.603	1008812	19937	50.256	58.083
2	15.892	998544	14388	49.744	41.917
Total		2007356	34325	100.000	100.000

Racemic **3g**

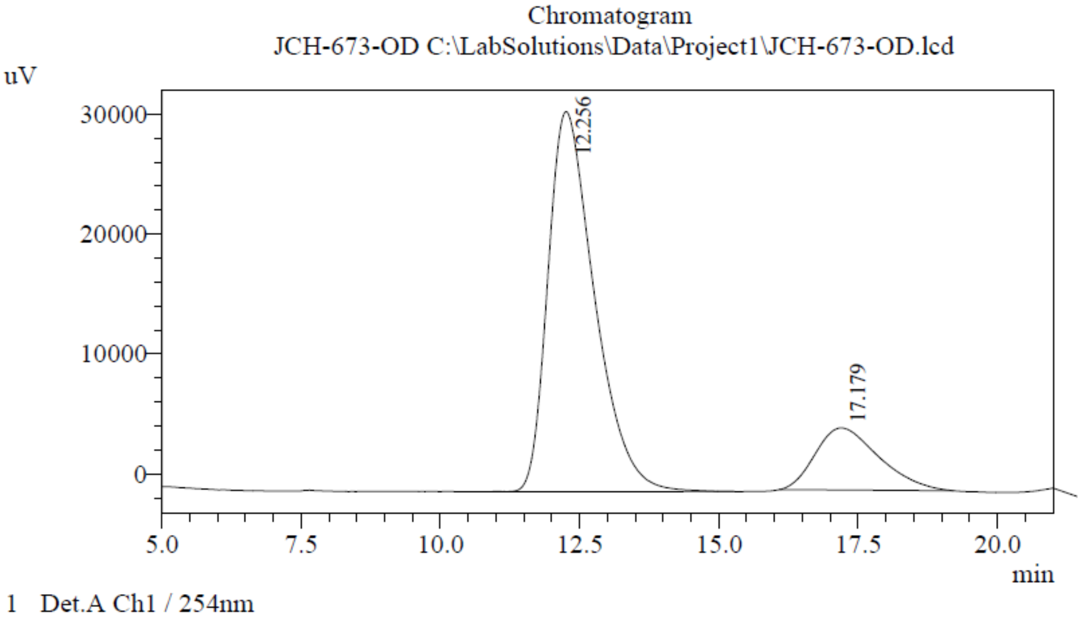

PeakTable

Detector A Ch1 254nm

Peak#	Ret. Time	Area	Height	Area %	Height %
1	12.256	1845022	31716	81.752	85.976
2	17.179	411842	5173	18.248	14.024
Total		2256865	36889	100.000	100.000

Enantiomerically enriched **3g**

(*R*)-*N*-(1-(4-Bromophenyl)-3-oxo-3-phenylpropyl)-4-methoxybenzenesulfonamide (**3h**)

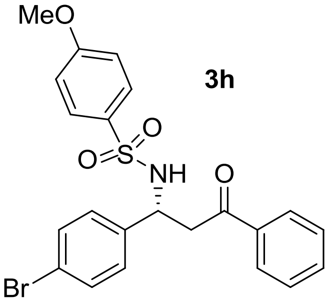

3h

A white solid; $[\alpha]^{25}_{D}$ = +55.6 (*c* 1.00, $CHCl_3$); ^{1}H NMR (500 MHz, $CDCl_3$) δ 7.80 (d, *J* = 8.0 Hz, 2H), 7.66–7.49 (m, 3H), 7.42 (t, *J* = 7.6 Hz, 2H), 7.29 (d, *J* = 8.3 Hz, 2H), 7.05 (d, *J* = 8.3 Hz, 2H), 6.81 (d, *J* = 8.7 Hz, 2H), 5.87 (d, *J* = 7.0 Hz, 1H), 4.82 (q, *J* = 6.1 Hz, 1H), 3.83 (s, 3H), 3.52 (dd, *J* = 17.4 Hz, 5.7 Hz, 1H), 3.42 (dd, *J* = 17.4 Hz, 6.0 Hz, 1H); ^{13}C NMR (126 MHz, $CDCl_3$) δ 197.53, 162.84, 138.93, 136.13, 133.75, 131.67, 131.53, 129.26, 128.71, 128.60, 128.02, 121.51, 114.01, 55.58, 53.90, 44.46; HRMS (ESI) *m*/*z*: calcd for

$C_{22}H_{20}BrNO_4S$ [M + Na]$^+$ 496.0189, found 496.0179; The *ee* value was 61%, t_R (major) = 13.3 min, t_R (minor) = 20.6 min (Chiralcel OD-H, λ = 254 nm, 30% iPrOH/hexanes, flow rate = 1.0 mL/min).

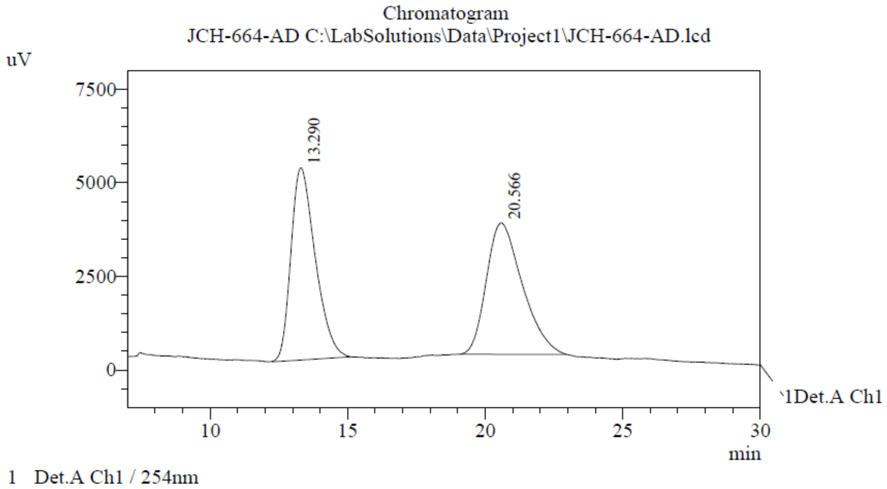

PeakTable

Detector A Ch1 254nm

Peak#	Ret. Time	Area	Height	Area %	Height %
1	13.290	316131	5132	49.743	59.371
2	20.566	319403	3512	50.257	40.629
Total		635533	8645	100.000	100.000

Racemic **3h**

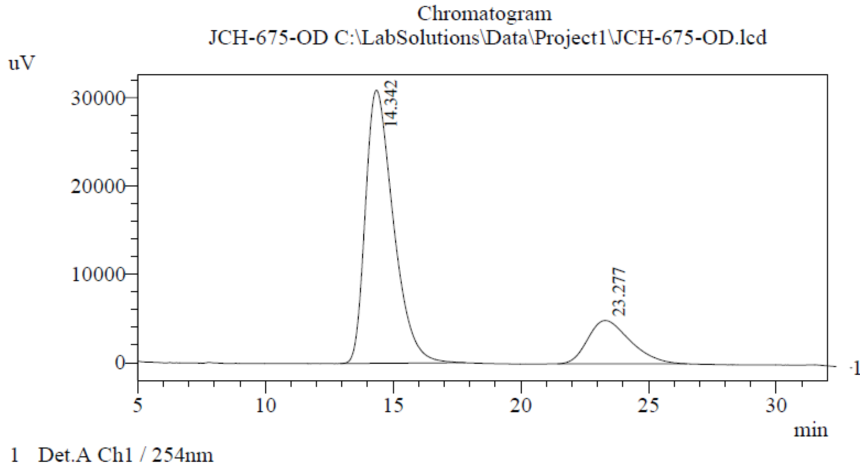

PeakTable

Detector A Ch1 254nm

Peak#	Ret. Time	Area	Height	Area %	Height %
1	14.342	2357825	30917	80.614	86.290
2	23.277	567025	4912	19.386	13.710
Total		2924850	35829	100.000	100.000

Enantiomerically enriched **3h**

(*R*)-4-Methoxy-*N*-(3-oxo-3-phenyl-1-(4-(trifluoromethyl)phenyl)propyl)benzene-sulfonamide (**3i**)

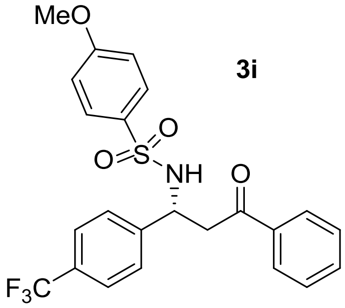

3i

A white solid; $[\alpha]^{25}_{D}$ = +63.0 (*c* 1.00, $CHCl_3$); ^{1}H NMR (500 MHz, $CDCl_3$) δ 7.80 (d, *J* = 7.6 Hz, 2H), 7.67–7.51 (m, 3H), 7.42 (t, *J* = 7.0 Hz, 4H), 7.31 (d, *J* = 8.0 Hz, 2H), 6.79 (d, *J* = 8.8 Hz, 2H), 5.98 (d, *J* = 7.2 Hz, 1H), 4.93 (dd, *J* = 12.2 Hz, 5.9 Hz, 1H), 3.80 (s, 3H), 3.55 (dd, *J* = 17.5 Hz, 5.7 Hz, 1H), 3.46 (dd, *J* = 17.5 Hz, 5.8 Hz, 1H); ^{13}C NMR (126 MHz, $CDCl_3$) δ 197.41, 162.89, 143.86, 136.06, 133.87, 131.63, 129.26, 128.76, 128.05, 127.32, 125.40 (d, *J* = 3.7 Hz), 114.01, 55.50, 54.02, 44.39; HRMS (ESI) *m/z*: calcd for $C_{23}H_{20}F_3NO_4S$ $[M + Na]^+$ 486.0957, found 486.0964; The *ee* value was 55%, t_R (major) = 10.5 min, t_R (minor) = 17.3 min (Chiralcel OD-H, λ = 254 nm, 30% iPrOH/hexanes, flow rate = 1.0 mL/min).

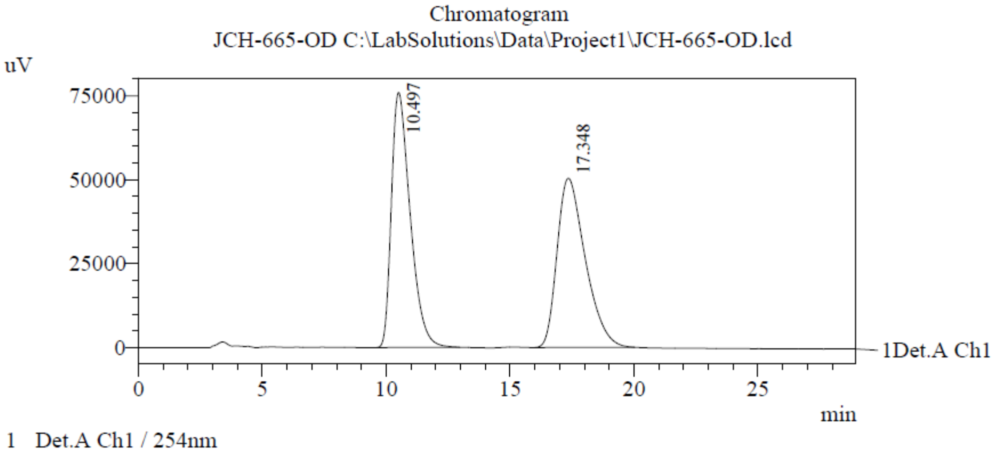

PeakTable

Detector A Ch1 254nm

Peak#	Ret. Time	Area	Height	Area %	Height %
1	10.497	4003136	75994	50.088	60.126
2	17.348	3989143	50397	49.912	39.874
Total		7992279	126391	100.000	100.000

Racemic **3i**

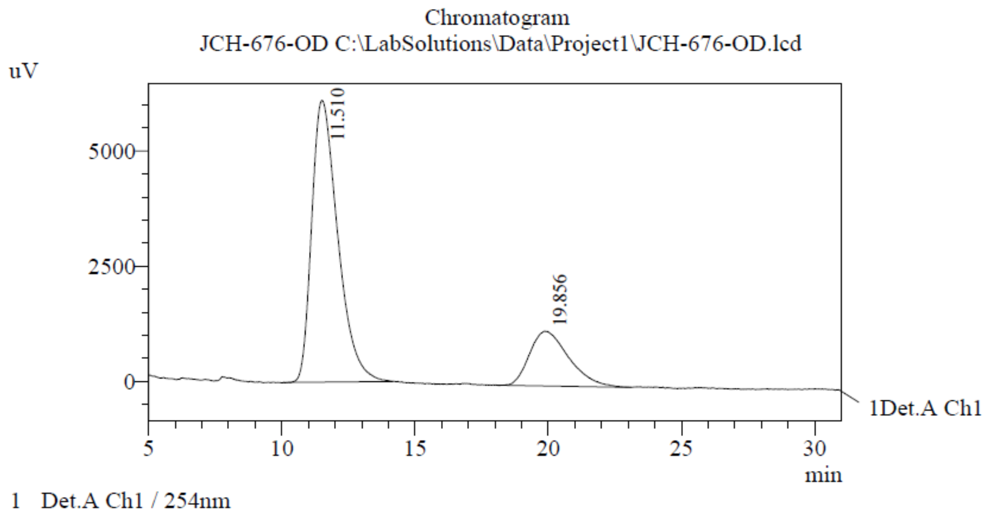

PeakTable

Detector A Ch1 254nm

Peak#	Ret. Time	Area	Height	Area %	Height %
1	11.510	416355	6111	77.354	83.713
2	19.856	121892	1189	22.646	16.287
Total		538248	7300	100.000	100.000

Enantiomerically enriched **3i**

(*R*)-4-Methoxy-N-(1-(4-methoxyphenyl)-3-oxo-3-phenylpropyl)benzenesulfonamide (**3j**)

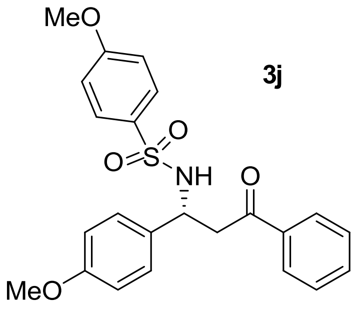

3j

A white solid; $[\alpha]^{25}_{D}$ = +77.4 (*c* 1.00, $CHCl_3$); ^{1}H NMR (500 MHz, $CDCl_3$) δ 7.82 (d, *J* = 7.5 Hz, 2H), 7.66 (t, *J* = 5.8 Hz, 2H), 7.55 (t, *J* = 7.3 Hz, 1H), 7.42 (t, *J* = 7.7 Hz, 2H), 7.12 (t, *J* = 7.9 Hz, 1H), 6.83 (d, *J* = 8.8 Hz, 2H), 6.79–6.64 (m, 3H), 5.64 (d, *J* = 6.7 Hz, 1H), 4.82 (q, *J* = 6.2 Hz, 1H), 3.82 (s, 3H), 3.69 (s, 3H), 3.57 (dd, *J* = 17.3 Hz, 5.7 Hz, 1H), 3.46 (dd, *J* = 17.3 Hz, 6.2 Hz, 1H); ^{13}C NMR (126 MHz, $CDCl_3$) δ 197.80, 162.75, 159.67, 141.47, 136.35, 133.58, 131.84, 129.61, 129.34, 128.65, 128.05, 118.90, 113.99, 113.19, 112.50, 55.54, 55.11, 54.42, 44.68; HRMS (ESI) *m*/*z*: calcd for $C_{23}H_{23}NO_5S$ $[M + Na]^+$ 448.1189, found 448.1180; The *ee* value was 62%, t_R (major) = 17.4 min, t_R (minor) = 23.3 min (Chiralcel OD-H, λ = 254 nm, 30% *i*-PrOH/hexanes, flow rate = 1.0 mL/min).

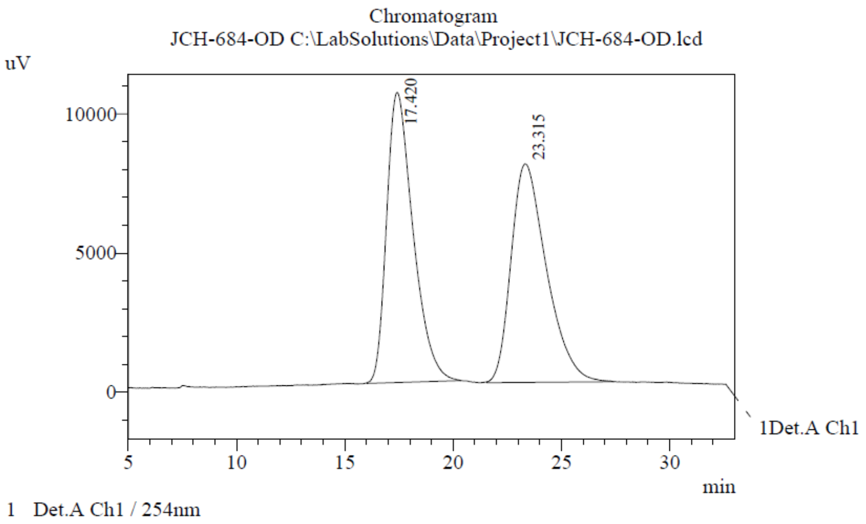

PeakTable

Detector A Ch1 254nm

Peak#	Ret. Time	Area	Height	Area %	Height %
1	17.420	886937	10423	49.835	57.033
2	23.315	892826	7852	50.165	42.967
Total		1779763	18275	100.000	100.000

Racemic **3j**

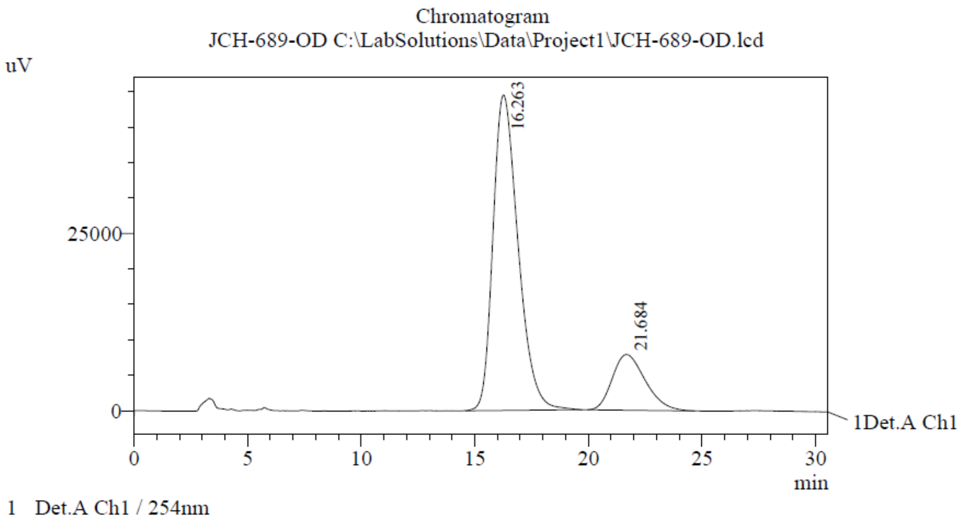

PeakTable

Detector A Ch1 254nm

Peak#	Ret. Time	Area	Height	Area %	Height %
1	16.263	3425631	44531	81.161	84.985
2	21.684	795136	7867	18.839	15.015
Total		4220767	52399	100.000	100.000

Enantiomerically enriched **3j**

(*R*)-*N*-(1-(2-Fluorophenyl)-3-oxo-3-phenylpropyl)-4-methoxybenzenesulfonamide (**3k**)

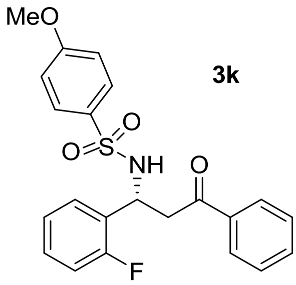

3k

A white solid; $[\alpha]^{25}_{D}$ = +33.6 (*c* 1.00, $CHCl_3$); ^{1}H NMR (500 MHz, $CDCl_3$) δ 7.80 (dd, *J* = 8.3 Hz, 1.2 Hz, 2H), 7.67–7.61 (m, 2H), 7.58–7.50 (m, 1H), 7.41 (t, *J* = 7.8 Hz, 2H), 7.29 (td, *J* = 7.7 Hz, 1.6 Hz, 2H), 7.13 (tdd, *J* = 7.3 Hz, 5.4, 1.7 Hz, 1H), 6.97 (td, *J* = 7.6 Hz, 1.1 Hz, 1H), 6.89 (ddd, *J* = 11.0 Hz, 8.2 Hz, 1.0 Hz, 1H), 6.77 (d, *J* = 8.9 Hz, 2H), 5.79 (d, *J* = 8.5 Hz, 1H), 5.11 (dt, *J* = 8.4 Hz, 6.0 Hz, 1H), 3.79 (s, 3H), 3.54–3.47 (m, 2H); ^{13}C NMR (126 MHz, $CDCl_3$) δ 197.43, 162.72, 160.84, 158.89, 136.14, 133.64, 131.66, 129.41, 129.37, 129.33, 129.26, 129.19,128.67, 128.07, 126.97, 126.87, 124.17, 124.15, 115.52, 115.34, 113.96, 55. HRMS (ESI) *m*/*z*: calcd for $C_{22}H_{20}FNO_4S$ $[M + Na]^+$ 436.0989, found 436.0974; The *ee* value was 65%, t_R (major) = 46.5 min, t_R (minor) = 55.1 min (Chiralcel ID-H, λ = 254 nm, 30% iPrOH/hexanes, flow rate = 1.0 mL/min).

<Chromatogram>

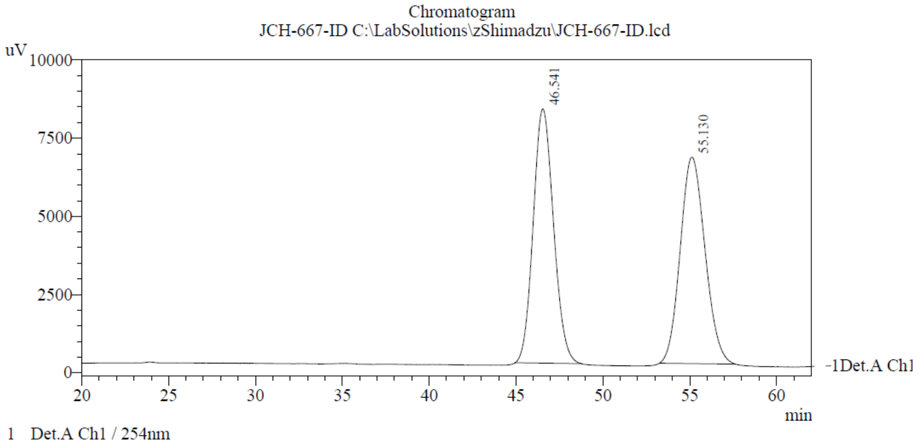

PeakTable

Detector A Ch1 254nm

Peak#	Ret. Time	Area	Height	Area %	Height %
1	46.541	662776	8134	50.259	55.183
2	55.130	655949	6606	49.741	44.817
Total		1318726	14740	100.000	100.000

Racemic **3k**

<Chromatogram>

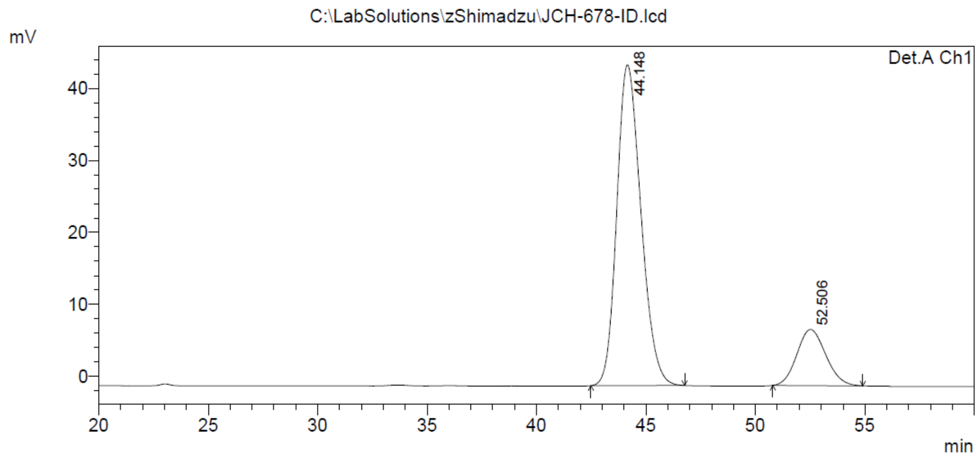

1 Det.A Ch1/254nm

PeakTable

Detector A Ch1 254nm

Peak#	Ret. Time	Area	Height	Area %	Height %
1	44.148	3425598	44597	82.706	85.117
2	52.506	716281	7798	17.294	14.883
Total		4141879	52395	100.000	100.000

Enantiomerically enriched **3k**

(*R*)-4-Methoxy-N-(3-oxo-3-phenyl-1-*o*-tolylpropyl)benzenesulfonamide (**3l**)

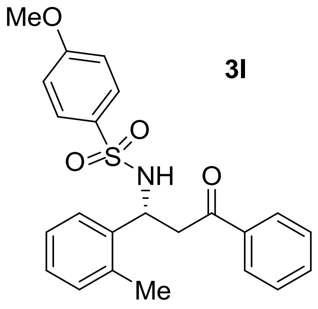

3l

A white solid; $[\alpha]^{25}_{D}$ = +42.4 (*c* 1.00, $CHCl_3$); ^{1}H NMR (500 MHz, $CDCl_3$) δ 7.86–7.79 (m, 1H), 7.65–7.60 (m, 1H), 7.54 (t, *J* = 7.4 Hz, 1H), 7.41 (t, *J* = 7.8 Hz, 1H), 7.27–7.20 (m, 1H), 7.11–6.98 (m, 1H), 6.81 (d, *J* = 8.9 Hz, 1H), 5.47 (d, *J* = 5.6 Hz, 1H), 5.10 (q, *J* = 6.2 Hz, 1H), 3.81 (s, 3H), 3.61 (dd, *J* = 17.2 Hz, 6.0 Hz, 1H), 3.50 (dd, *J* = 17.2 Hz, 6.7 Hz, 1H), 2.18 (s, 3H); ^{13}C NMR (126 MHz, $CDCl_3$) δ 197.76, 162.73, 137.98, 136.39, 135.28, 133.48, 131.66, 130.61, 129.29, 128.62, 128.03, 127.64, 126.36, 113.94, 55.54, 50.43, 44.64, 19.10; HRMS (ESI) *m*/*z*: calcd for $C_{23}H_{23}NO_4S$ $[M + Na]^+$ 432.1240, found 432.1240; The *ee* value was 65%, t_R (major) = 28.9 min, t_R (minor) = 38.1 min (Chiralcel OD-H, λ = 254 nm, 30% iPrOH/hexanes, flow rate = 1.0 mL/min).

<Chromatogram>

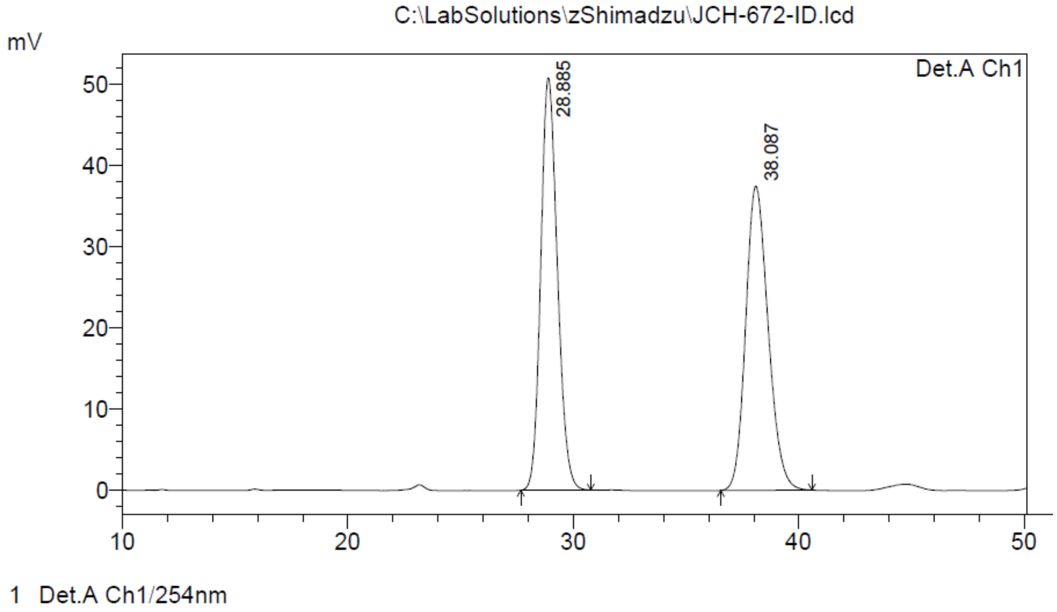

PeakTable

Detector A Ch1 254nm

Peak#	Ret. Time	Area	Height	Area %	Height %
1	28.885	2606214	50772	50.059	57.549
2	38.087	2600055	37452	49.941	42.451
Total		5206269	88224	100.000	100.000

Racemic **3l**

<Chromatogram>

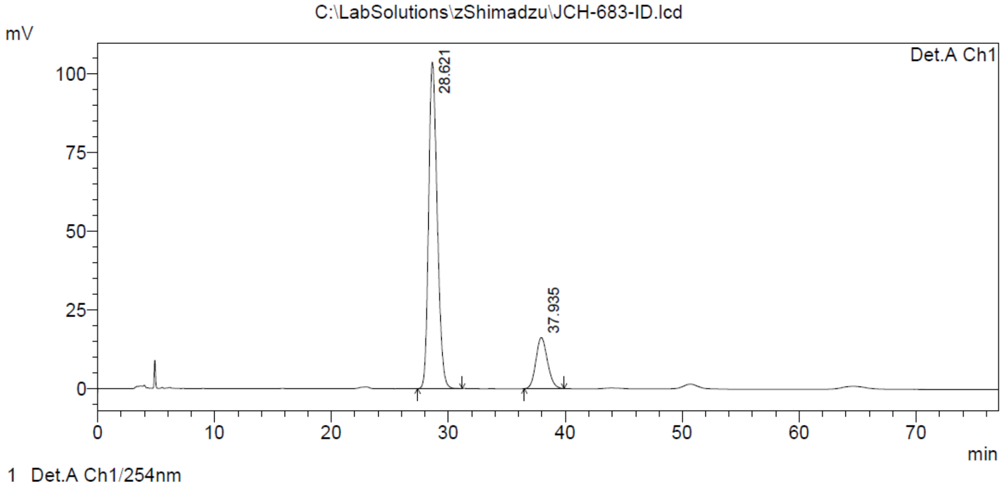

PeakTable

Detector A Ch1 254nm

Peak#	Ret. Time	Area	Height	Area %	Height %
1	28.621	5311807	103730	82.710	86.470
2	37.935	1110382	16231	17.290	13.530
Total		6422189	119961	100.000	100.000

Enantiomerically enriched **3l**

(R)-N-(1-(2-Bromophenyl)-3-oxo-3-phenylpropyl)-4-methoxybenzenesulfonamide (**3m**)

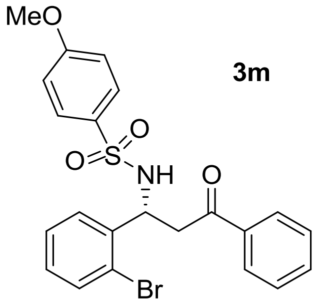

3m

A white solid; $[\alpha]^{25}_{D}$ = +40.4 (c 1.00, $CHCl_3$); ^1H NMR (500 MHz, $CDCl_3$) δ 7.84–7.77 (m, 2H), 7.69–7.57 (m, 2H), 7.54 (t, J = 7.4 Hz, 1H), 7.42 (ddd, J = 16.9 Hz, 9.5 Hz, 4.8 Hz, 4H), 7.14 (dd, J = 10.9 Hz, 4.3 Hz, 1H), 7.02 (td, J = 7.7 Hz, 1.6 Hz, 1H), 6.76 (d, J = 8.9 Hz, 2H), 6.12 (d, J = 7.2 Hz, 1H), 5.17 (dd, J = 12.8 Hz, 6.1 Hz, 1H), 4.12 (q, J = 7.1 Hz, 1H), 3.78 (s, 3H), 3.45 (dd, J = 5.8 Hz, 3.4 Hz, 2H); ^{13}C NMR (126 MHz, $CDCl_3$) δ 197.98, 162.70, 138.66, 136.07, 133.71, 132.83, 131.31, 129.50, 129.30, 129.01, 128.63, 128.16, 127.53, 122.09, 113.89, 55.48, 53.93, 42.86; HRMS (ESI) m/z: calcd for $C_{22}H_{20}BrNO_4S$ $[M + Na]^+$ 496.0189, found 496.0182; The *ee* value was 59%, t_R (major) = 26.8 min, t_R (minor) = 23.7 min (Chiralcel OD-H, λ = 254 nm, 40% iPrOH/hexanes, flow rate = 0.7 mL/min).

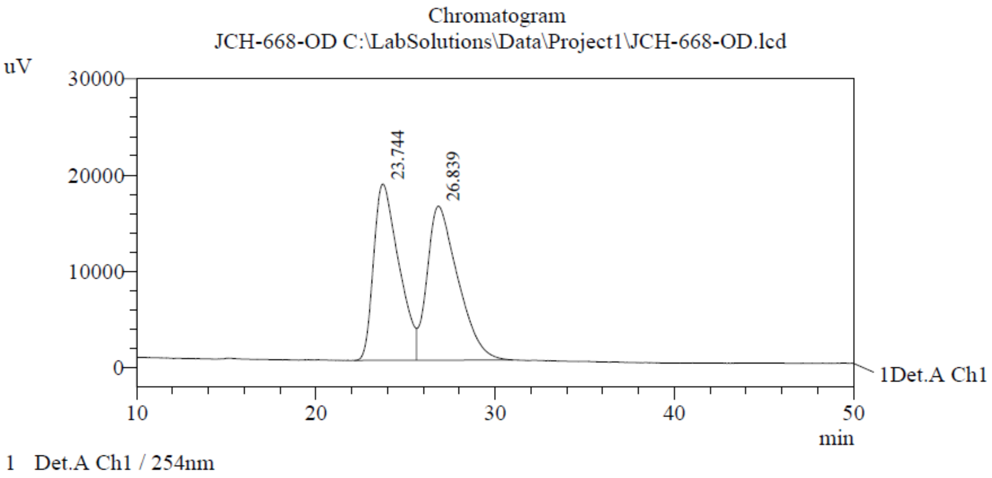

PeakTable

Detector A Ch1 254nm

Peak#	Ret. Time	Area	Height	Area %	Height %
1	23.744	1793094	18303	48.340	53.374
2	26.839	1916207	15989	51.660	46.626
Total		3709302	34291	100.000	100.000

Racemic **3m**

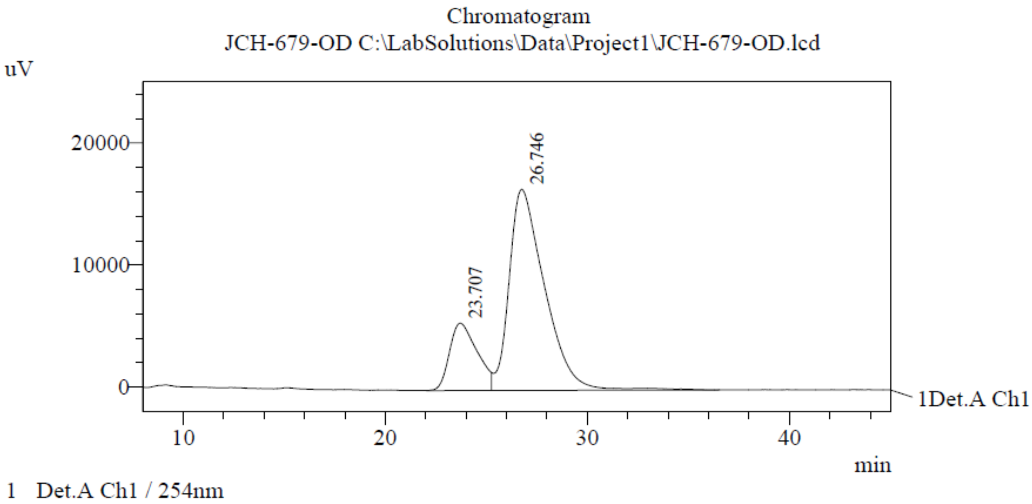

PeakTable

Detector A Ch1 254nm

Peak#	Ret. Time	Area	Height	Area %	Height %
1	23.707	523265	5506	20.590	25.072
2	26.746	2018065	16454	79.410	74.928
Total		2541331	21959	100.000	100.000

Enantiomerically enriched **3m**

(*R*)-4-Methoxy-N-(3-oxo-3-phenyl-1-*m*-tolylpropyl)benzenesulfonamide (**3n**)

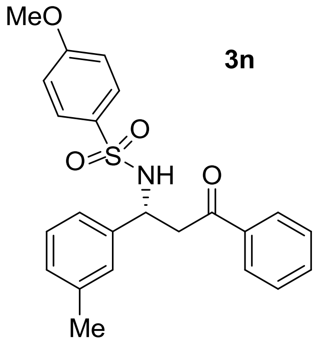

A white solid; $[\alpha]^{25}_{D}$ = +35.4 (*c* 1.00, $CHCl_3$); ^{1}H NMR (500 MHz, $CDCl_3$) δ 7.85–7.78 (m, 2H), 7.67–7.47 (m, 3H), 7.48 (d, *J* = 7.9 Hz, 1H), 7.41 (t, *J* = 7.8 Hz, 2H), 7.08 (t, *J* = 7.6 Hz, 1H), 6.96 (d, *J* = 7.6 Hz, 2H), 6.90 (s, 1H), 6.87–6.70 (m, 2H), 5.67 (d, *J* = 6.8 Hz, 1H), 4.82 (q, *J* = 6.2 Hz, 1H), 3.82 (s, 3H), 3.57 (dd, *J* = 17.3 Hz, 5.7 Hz, 1H), 3.46 (dd, *J* = 17.3 Hz, 6.3 Hz, 1H), 2.21 (s, 3H); ^{13}C NMR (126 MHz, $CDCl_3$) δ 197.77, 162.67, 139.75, 138.16, 136.34, 133.52, 131.82, 129.31, 128.62, 128.43, 128.04, 127.48, 123.69, 113.89, 55.51, 54.42, 44.83, 21.27; HRMS (ESI) *m*/*z*: calcd for $C_{23}H_{23}NO_4S$ $[M + Na]^+$ 432.1240, found 432.1240; The *ee* value was 65%, t_R (major) = 10.6 min, t_R (minor) = 14.6 min (Chiralcel OD-H, λ = 254 nm, 30% iPrOH/hexanes, flow rate = 1.0 mL/min).

<Chromatogram>

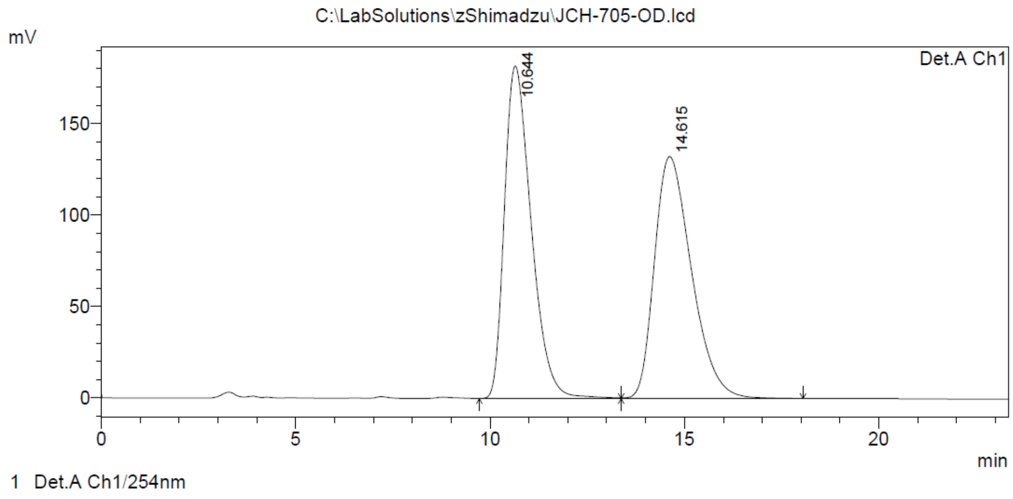

PeakTable

Detector A Ch1 254nm

Peak#	Ret. Time	Area	Height	Area %	Height %
1	10.644	8730449	181906	49.884	57.889
2	14.615	8770977	132329	50.116	42.111
Total		17501426	314234	100.000	100.000

Racemic **3n**

<Chromatogram>

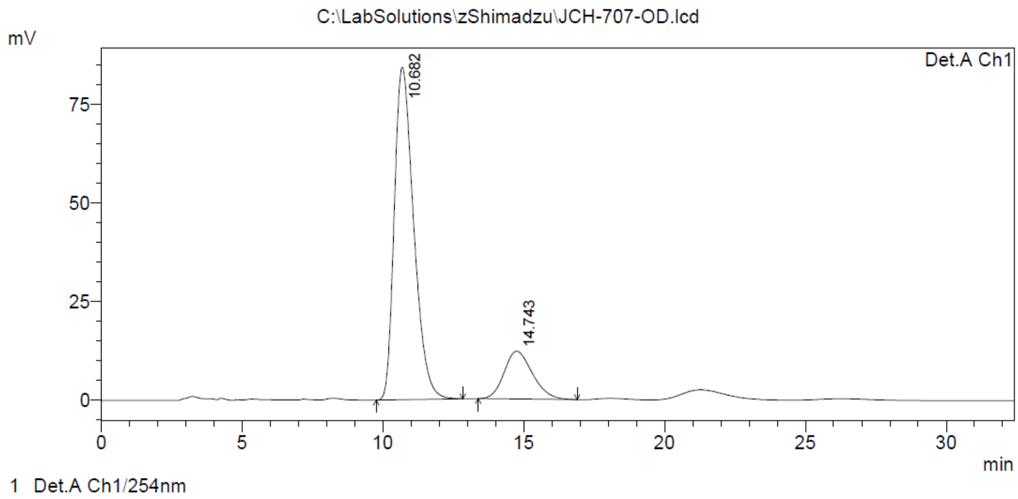

PeakTable

Detector A Ch1 254nm

Peak#	Ret. Time	Area	Height	Area %	Height %
1	10.682	4036364	84288	82.616	87.412
2	14.743	849320	12138	17.384	12.588
Total		4885685	96425	100.000	100.000

Enantiomerically enriched **3n**

(*R*)-*N*-(1-(3-Bromophenyl)-3-oxo-3-phenylpropyl)-4-methoxybenzenesulfonamide (**3o**)

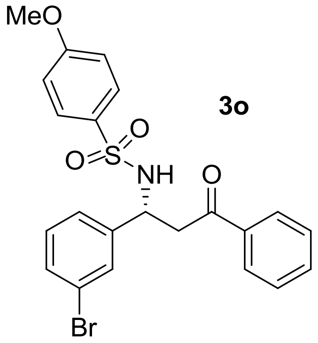

3o

A white solid; $[\alpha]^{25}_{D}$ = +37.4 (*c* 1.00, $CHCl_3$); ^{1}H NMR (500 MHz, $CDCl_3$) δ 7.81 (dd, *J* = 8.3 hz, 1.1 Hz, 2H), 7.65–7.52 (m, 3H), 7.42 (t, *J* = 7.8 Hz, 2H), 7.23 (t, *J* = 1.7 Hz, 1H), 7.14 (d, *J* = 7.8 Hz, 1H), 7.06 (t, *J* = 7.8 Hz, 1H), 6.82 (d, *J* = 8.9 Hz, 2H), 5.85 (d, *J* = 7.1 Hz, 1H), 4.83 (dd, *J* = 12.8 Hz, 6.0 Hz, 1H), 3.83 (s, 3H), 3.52 (dd, *J* = 17.4 Hz, 5.8 Hz, 1H), 3.44 (dd, *J* = 17.4 Hz, 5.9 Hz, 1H); ^{13}C NMR (126 MHz, $CDCl_3$) δ 197.43, 162.82, 142.05, 136.09, 133.77, 131.59, 130.71, 130.03, 129.23, 128.71, 128.05, 125.46, 122.56, 114.04, 55.56, 53.90, 44.53; HRMS (ESI) *m*/*z*: calcd for $C_{22}H_{20}BrNO_4S$ $[M + Na]^+$ 496.0189, found 496.0182; The *ee* value was 61%, t_R (major) = 12.3 min, t_R (minor) = 19.0 min (Chiralcel OD-H, λ = 254 nm, 30% iPrOH/hexanes, flow rate = 1.0 mL/min).

<Chromatogram>

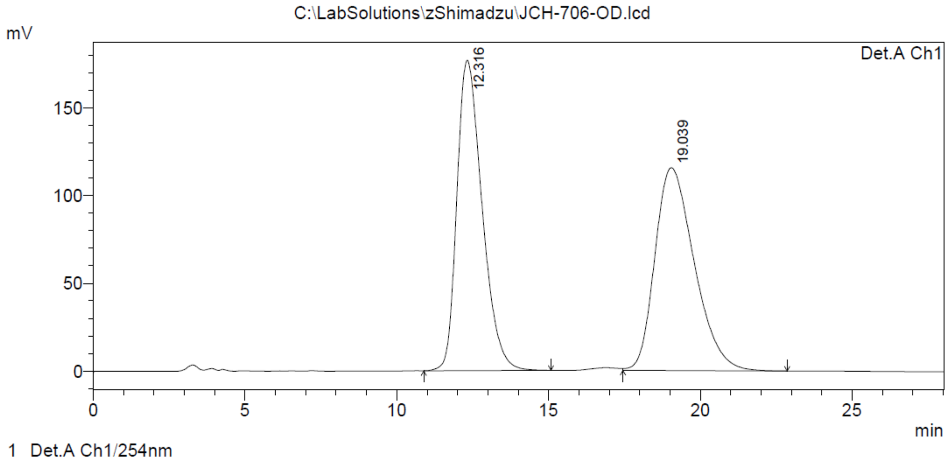

PeakTable

Detector A Ch1 254nm

Peak#	Ret. Time	Area	Height	Area %	Height %
1	12.316	10216376	176725	49.494	60.472
2	19.039	10425112	115516	50.506	39.528
Total		20641488	292240	100.000	100.000

Racemic **3o**

<Chromatogram>

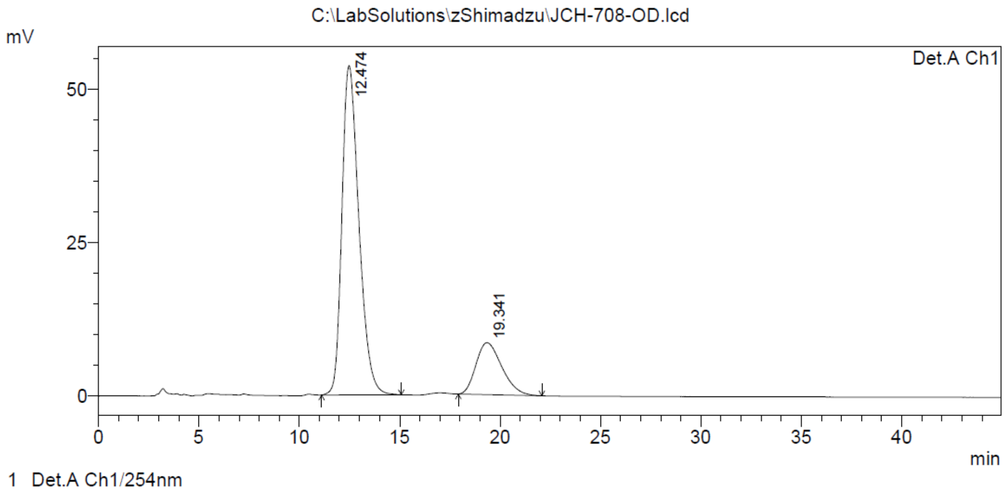

PeakTable

Detector A Ch1 254nm

Peak#	Ret. Time	Area	Height	Area %	Height %
1	12.474	3154260	53707	80.570	86.379
2	19.341	760692	8469	19.430	13.621
Total		3914951	62176	100.000	100.000

Enantiomerically enriched **3o**

(*R*)-*N*-(1-(Furan-2-yl)-3-oxo-3-phenylpropyl)-4-methoxybenzenesulfonamide (**3p**)

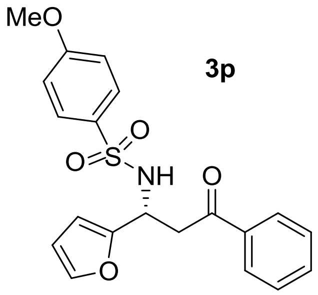

3p

A white solid; $[\alpha]^{25}_{D}$ = +27.6 (*c* 1.00, $CHCl_3$); ^{1}H NMR (500 MHz, $CDCl_3$) δ 7.86 (dd, *J* = 8.3 Hz, 1.2 Hz, 2H), 7.77–7.69 (m, 2H), 7.60–7.53 (m, 1H), 7.44 (dd, *J* = 11.1 Hz, 4.6 Hz, 2H), 7.15 (dd, *J* = 1.8 Hz, 0.8 Hz, 1H), 6.88 (d, *J* = 9.0 Hz, 2H), 6.16 (dd, *J* = 3.3 Hz, 1.8 Hz, 1H), 6.03 (dd, *J* = 2.4 Hz, 1.6 Hz, 1H), 5.69 (d, *J* = 8.6 Hz, 1H), 5.03–4.95 (m, 1H), 3.83 (s, 3H), 3.65 (dd, *J* = 17.4 Hz, 4.7 Hz, 1H), 3.46 (dd, *J* = 17.4 Hz, 6.5 Hz, 1H); ^{13}C NMR (126 MHz, $CDCl_3$) δ 197.32, 162.77, 152.27, 141.84, 136.27, 133.59, 132.04, 129.23, 128.66, 128.04, 114.06, 110.44, 107.24, 55.55, 48.29, 42.17; HRMS (ESI) *m*/*z*: calcd for $C_{20}H_{19}NO_5S$ $[M + Na]^+$ 408.0876, found 408.0863; The *ee* value was 83%, t_R (major) = 13.1min, t_R (minor) = 15.9min (Chiralcel OD-H, λ = 254 nm, 30% iPrOH/hexanes, flow rate = 1.0 mL/min).

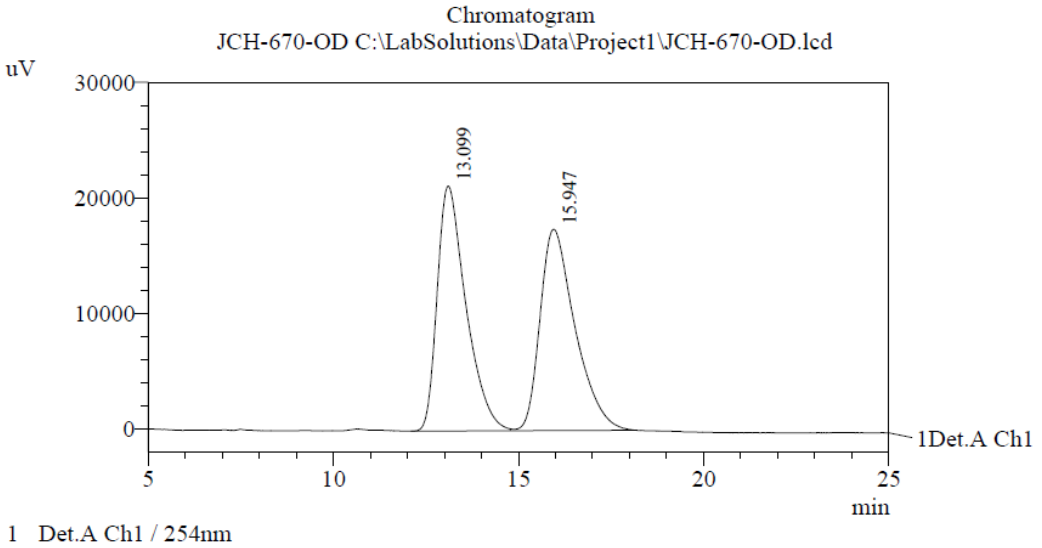

PeakTable

Detector A Ch1 254nm

Peak#	Ret. Time	Area	Height	Area %	Height %
1	13.099	1148101	21211	50.013	54.894
2	15.947	1147494	17429	49.987	45.106
Total		2295595	38640	100.000	100.000

Racemic **3p**

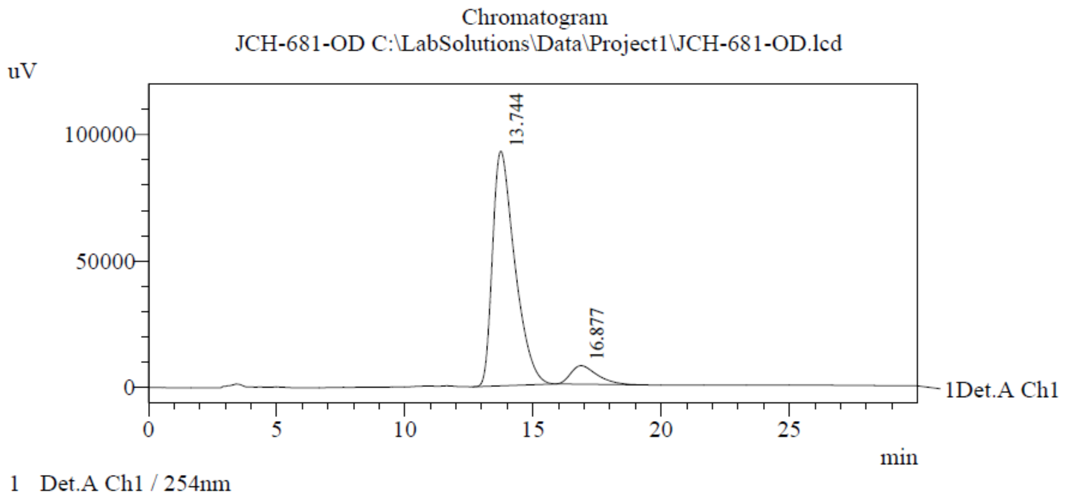

PeakTable

Detector A Ch1 254nm

Peak#	Ret. Time	Area	Height	Area %	Height %
1	13.744	5623841	92720	91.492	92.703
2	16.877	522979	7298	8.508	7.297
Total		6146819	100018	100.000	100.000

Enantiomerically enriched **3p**

(*R*)-4-Methoxy-*N*-(3-oxo-3-phenyl-1-(thiophen-2-yl)propyl)benzenesulfonamide (**3q**)

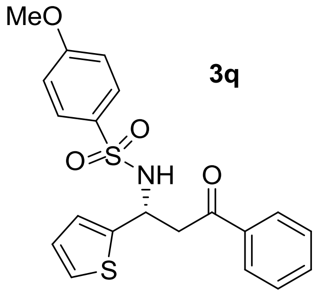

3q

A white solid; $[\alpha]^{25}_{D}$ = −5.0 (*c* 1.00, $CHCl_3$); ^{1}H NMR (500 MHz, $CDCl_3$) δ 7.89–7.82 (m, 2H), 7.78–7.64 (m, 2H), 7.57 (t, *J* = 7.4 Hz, 1H), 7.44 (t, *J* = 7.8 Hz, 2H), 7.09 (dd, *J* = 5.0 Hz, 1.1 Hz, 1H), 6.90–6.84 (m, 2H), 6.81–6.74 (m, 2H), 5.82 (d, *J* = 8.1 Hz, 1H), 5.20–5.11 (m, 1H), 3.83 (s, 3H), 3.68 (dd, *J* = 17.6 Hz, 4.5 Hz, 1H), 3.55 (dd, *J* = 17.6 Hz, 6.2 Hz, 1H); ^{13}C NMR (126 MHz, $CDCl_3$) δ 197.59, 162.80, 143.68, 136.29, 133.69, 131.91, 129.29, 128.69, 128.04, 126.66, 125.15, 125.08, 114.06, 55.55, 50.25, 44.85; HRMS (ESI) *m/z*: calcd for $C_{20}H_{19}NO_4S_2$ $[M + Na]^+$ 424.0648, found 424.0651; The *ee* value was 77%, t_R (major) = 16.8 min, t_R (minor) = 20.6 min (Chiralcel OD-H, λ = 254 nm, 30% iPrOH/hexanes, flow rate = 1.0 mL/min).

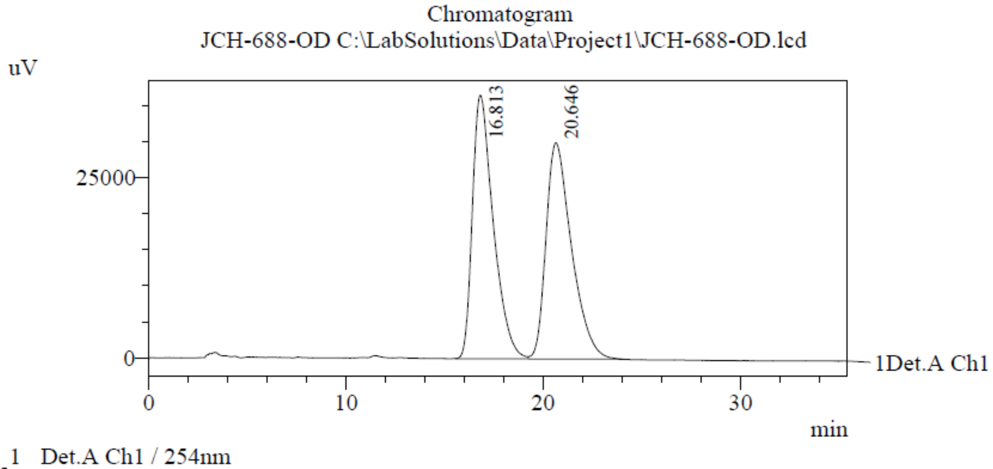

1 Det.A Ch1 / 254nm
Detector A Ch1 254nm

Peak#	Ret. Time	Area	Height	Area %	Height %
1	16.813	2702096	36597	49.940	54.936
2	20.646	2708591	30021	50.060	45.064
Total		5410687	66618	100.000	100.000

Racemic **3q**

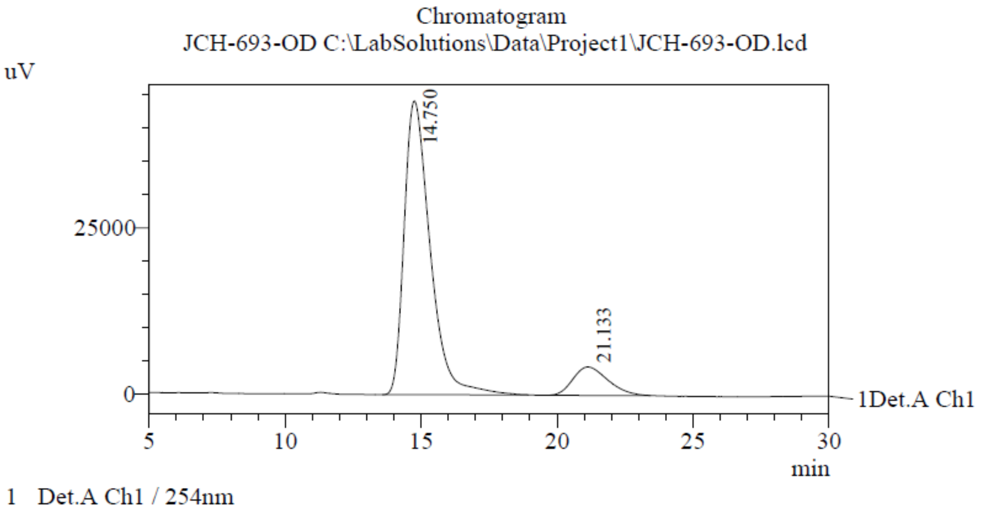

PeakTable

Detector A Ch1 254nm

Peak#	Ret. Time	Area	Height	Area %	Height %
1	14.750	2943386	44208	88.477	91.153
2	21.133	383321	4291	11.523	8.847
Total		3326707	48498	100.000	100.000

Enantiomerically enriched **3q**

(*R*)-*N*-(3-(4-Fluorophenyl)-3-oxo-1-phenylpropyl)-4-methoxybenzenesulfonamide (**3r**)

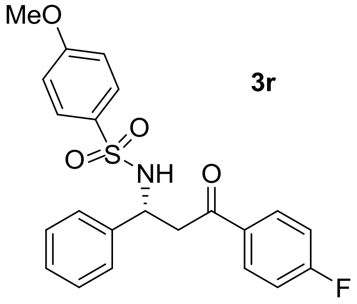

3r

A white solid; $[\alpha]^{25}_{D}$ = +35.8 (*c* 1.00, $CHCl_3$); ^{1}H NMR (500 MHz, $CDCl_3$) δ 7.87–7.81 (m, 2H), 7.65 (d, *J* = 8.9 Hz, 2H), 7.25–7.12 (m, 5H), 7.08 (t, *J* = 8.6 Hz, 2H), 6.86–6.80 (m, 2H), 5.67 (d, *J* = 6.8 Hz, 1H), 4.84 (dd, *J* = 12.5 Hz, 6.2 Hz, 1H), 3.82 (s, 3H), 3.57 (dd, *J* = 17.2 Hz, 5.6 Hz, 1H), 3.44 (dd, *J* = 17.1 Hz, 6.3 Hz, 1H); ^{13}C NMR (126 MHz, $CDCl_3$) δ 196.12, 165.11, 162.78, 139.81, 131.76, 130.79, 130.71, 129.31, 128.60, 127.75, 126.68, 115.87, 115.70, 114.01, 55.54, 54.42, 44.79; HRMS (ESI) *m*/*z*: calcd for $C_{22}H_{20}FNO_4S$ $[M + Na]^+$ 436.0989, found 436.1002; The *ee* value was 64%, t_R (major) = 16.0 min, t_R (minor) = 26.0 min (Chiralcel OD-H, λ = 254 nm, 30% iPrOH/hexanes, flow rate = 1.0 mL/min).

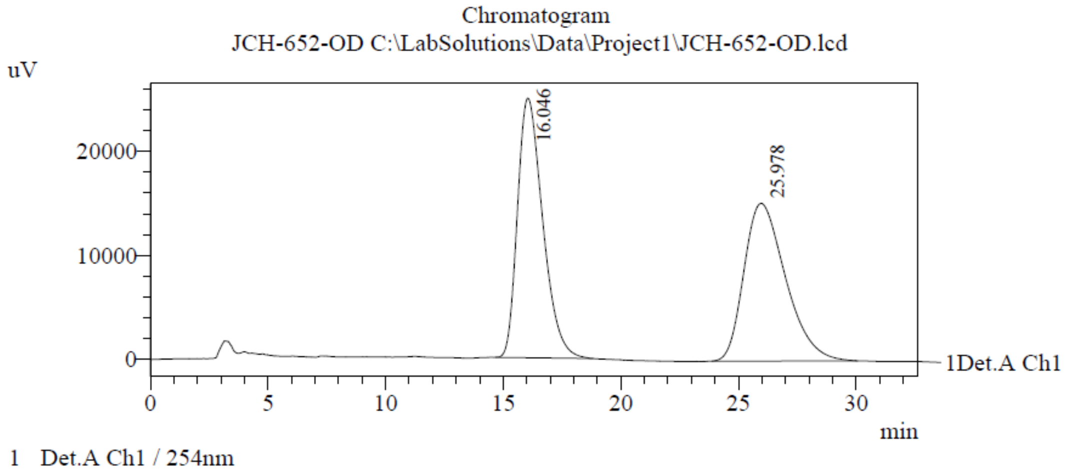

PeakTable

Detector A Ch1 254nm

Peak#	Ret. Time	Area	Height	Area %	Height %
1	16.046	1862410	24926	49.951	62.180
2	25.978	1866087	15161	50.049	37.820
Total		3728496	40087	100.000	100.000

Racemic **3r**

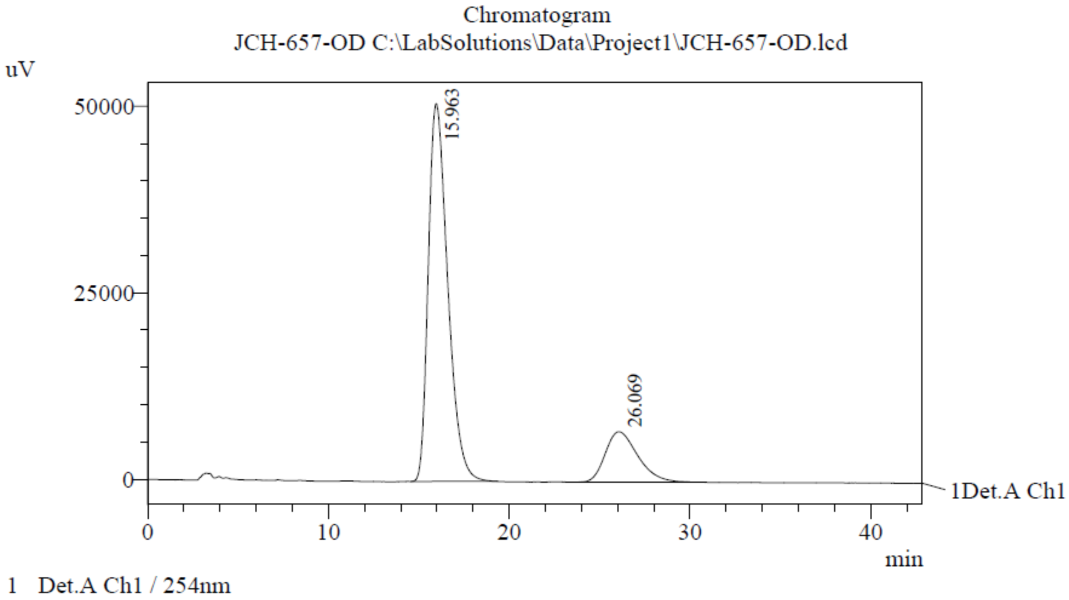

PeakTable

Detector A Ch1 254nm

Peak#	Ret. Time	Area	Height	Area %	Height %
1	15.963	3780474	50626	81.736	88.241
2	26.069	844764	6746	18.264	11.759
Total		4625239	57373	100.000	100.000

Enantiomerically enriched **3r**

(*R*)-*N*-(3-(3-Chlorophenyl)-3-oxo-1-phenylpropyl)-4-methoxybenzenesulfonamide (**3s**)

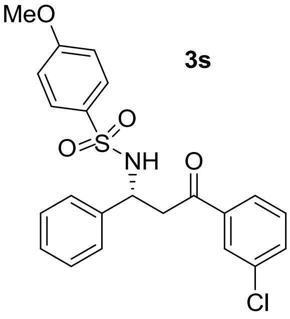

3s

A white solid; $[\alpha]^{25}_{D}$ = +30.8 (*c* 1.00, $CHCl_3$); ^1H NMR (500 MHz, $CDCl_3$) δ 7.77 (t, *J* = 1.8 Hz, 1H), 7.72–7.59 (m, 3H), 7.52 (ddd, *J* = 8.0 Hz, 2.0 Hz, 0.9 Hz, 1H), 7.36 (t, *J* = 7.9 Hz, 1H), 7.24–7.12 (m, 5H), 6.87–6.82 (m, 2H), 5.52 (d, *J* = 6.8 Hz, 1H), 4.84 (dd, *J* = 12.4 Hz, 6.3 Hz, 1H), 3.83 (s, 3H), 3.59 (dd, *J* = 17.3 Hz, 5.5 Hz, 1H), 3.45 (dd, *J* = 17.3 Hz, 6.4 Hz, 1H); ^{13}C NMR (126 MHz, $CDCl_3$) δ 196.47, 162.84, 139.69, 137.85, 135.07, 133.48, 131.70, 129.99, 129.34, 128.67, 128.16, 127.85, 126.66, 126.11, 114.05, 55.57, 54.31, 45.0; HRMS (ESI) *m*/*z*: calcd for $C_{22}H_{20}ClNO_4S$ $[M + Na]^+$ 452.0694, found 452.0693; The *ee* value was 70%, t_R (major) = 19.2 min, t_R (minor) = 24.7 min (Chiralcel OD-H, λ = 254 nm, 30% iPrOH/hexanes, flow rate = 1.0 mL/min).

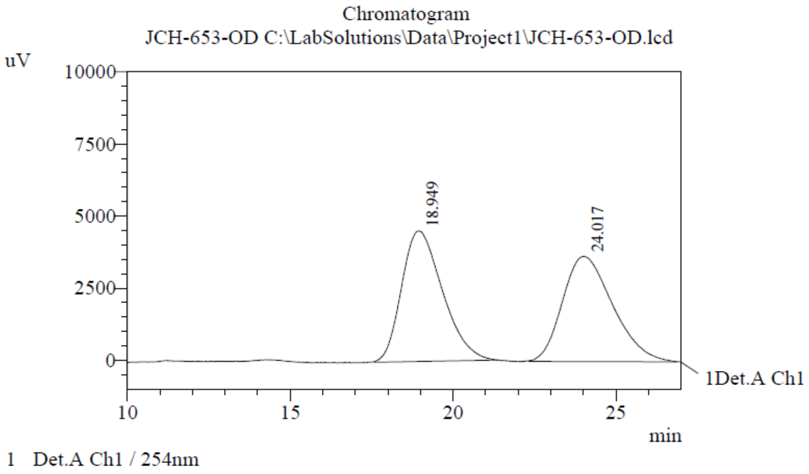

PeakTable

Detector A Ch1 254nm

Peak#	Ret. Time	Area	Height	Area %	Height %
1	18.949	395516	4523	50.279	55.377
2	24.017	391131	3645	49.721	44.623
Total		786647	8168	100.000	100.000

Racemic **3s**

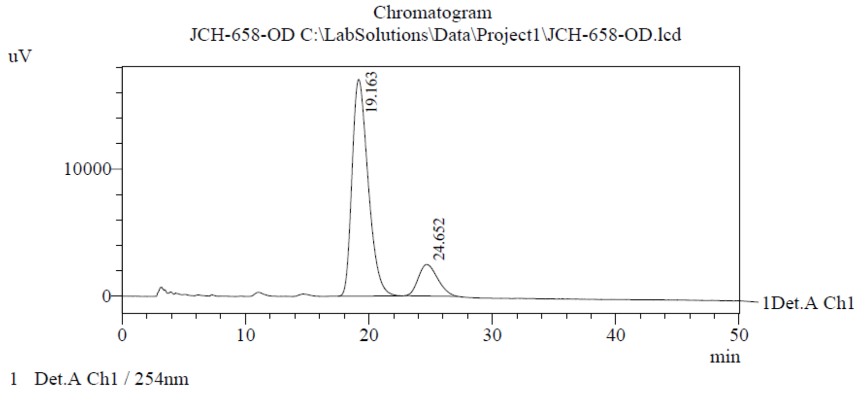

PeakTable

Detector A Ch1 254nm

Peak#	Ret. Time	Area	Height	Area %	Height %
1	19.163	1551089	17086	85.087	87.293
2	24.652	271864	2487	14.913	12.707
Total		1822953	19573	100.000	100.000

Enantiomerically enriched **3s**

(*R*)-4-Methoxy-*N*-(3-(naphthalen-2-yl)-3-oxo-1-phenylpropyl)benzenesulfonamide (**3t**)

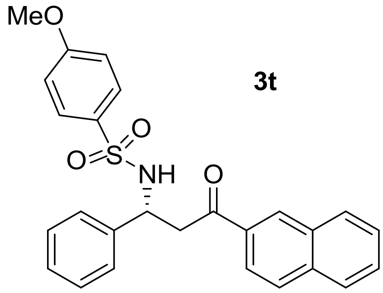

3t

A white solid; $[\alpha]^{25}_{D}$ = +21.8 (*c* 1.00, $CHCl_3$); ^1H NMR (500 MHz, $CDCl_3$) δ 8.32 (s, 1H), 7.87 (ddd, *J* = 18.1 Hz, 15.8 Hz, 8.2 Hz, 4H), 7.74–7.61 (m, 2H), 7.61–7.51 (m, 2H), 7.24–7.05 (m, 5H), 6.81 (d, *J* = 8.9 Hz, 2H), 5.71 (d, *J* = 6.7 Hz, 1H), 4.90 (q, *J* = 6.1 Hz, 1H), 3.77 (s, 3H), 3.71 (dd, *J* = 17.1 Hz, 5.8 Hz, 1H), 3.59 (dd, *J* = 17.1 Hz, 6.2 Hz, 1H); ^{13}C NMR (126 MHz, $CDCl_3$) δ 197.73, 162.74, 140.01, 135.75, 133.67, 132.35, 131.81, 130.02, 129.62, 129.34, 128.79, 128.60, 128.55, 127.77, 127.73, 126.94, 126.75, 123.50, 114.00, 55.48, 54.62, 44.83; HRMS (ESI) *m*/*z*: calcd for $C_{26}H_{23}NO_4S$ $[M + Na]^+$ 468.1240, found 468.1244; The *ee* value was 69%, t_R (major) = 21.9 min, t_R (minor) = 29.9 min (Chiralcel OD-H, λ = 254 nm, 30% iPrOH/hexanes, flow rate = 1.0 mL/min).

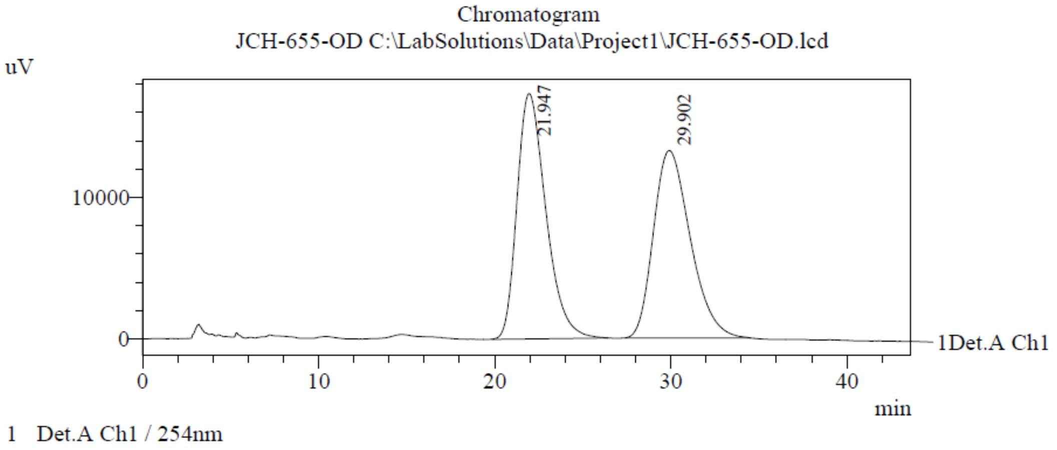

PeakTable

Detector A Ch1 254nm

Peak#	Ret. Time	Area	Height	Area %	Height %
1	21.947	1976530	17333	50.648	56.700
2	29.902	1925921	13237	49.352	43.300
Total		3902451	30570	100.000	100.000

Racemic **3t**

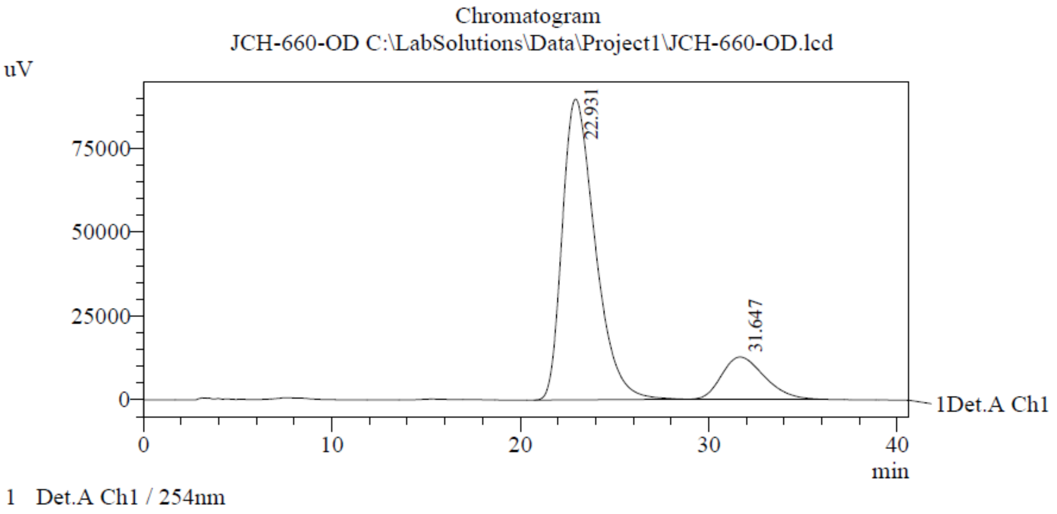

PeakTable

Detector A Ch1 254nm

Peak#	Ret. Time	Area	Height	Area %	Height %
1	22.931	10905092	89716	84.578	87.686
2	31.647	1988381	12599	15.422	12.314
Total		12893473	102315	100.000	100.000

Enantiomerically enriched **3t**

(*R*)-4-Methoxy-*N*-(3-oxo-1-phenyl-3-*p*-tolylpropyl)benzenesulfonamide (**3u**)

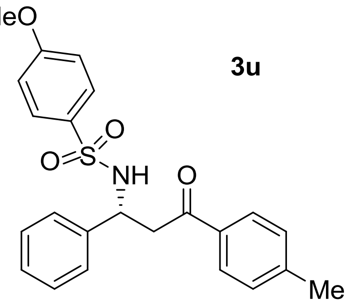

3u

A white solid; $[\alpha]^{25}_{D}$ = +23.8 (*c* 1.00, $CHCl_3$); ^{1}H NMR (500 MHz, $CDCl_3$) δ 7.75–7.63 (m, 4H), 7.18 (ddd, *J* = 7.0 Hz, 6.4 Hz, 5.2 Hz, 7H), 6.85–6.80 (m, 2H), 5.70 (d, *J* = 6.7 Hz, 1H), 4.83 (q, *J* = 6.1 Hz, 1H), 3.82 (s, 3H), 3.53 (dd, *J* = 17.2 Hz, 5.8 Hz, 1H), 3.42 (dd, *J* = 17.1 Hz, 6.1 Hz, 1H), 2.39 (s, 3H); ^{13}C NMR (126 MHz, $CDCl_3$) δ 197.47, 162.72, 144.54, 140.01, 133.92, 131.88, 129.32, 128.52, 128.18, 127.62, 126.73, 113.98, 55.52, 54.54, 44.54, 21.64; HRMS (ESI) *m/z*: calcd for $C_{23}H_{23}NO_4S$ $[M + Na]^+$ 432.1240, found 432.1250; The *ee* value was 67%, t_R (major) = 14.3 min, t_R (minor) = 20.1 min (Chiralcel OD-H, λ = 254 nm, 30% iPrOH/hexanes, flow rate = 1.0 mL/min).

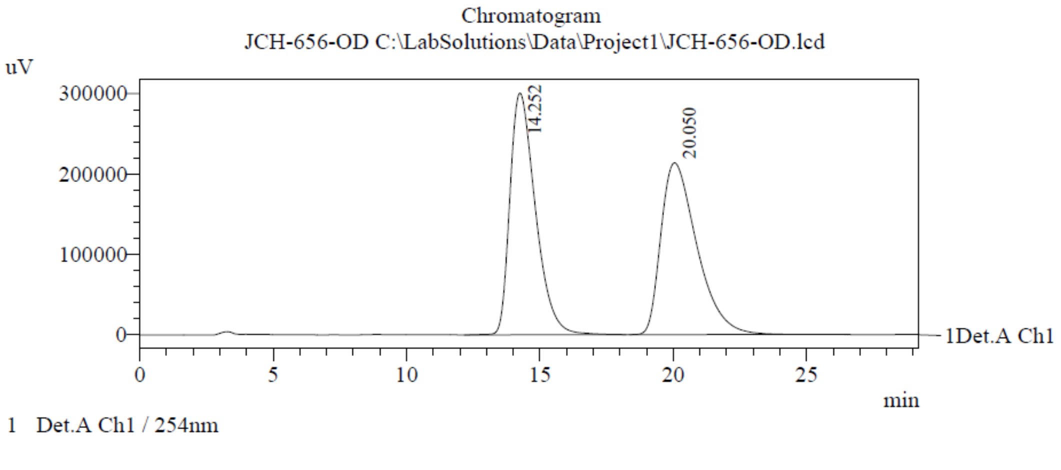

PeakTable

Detector A Ch1 254nm

Peak#	Ret. Time	Area	Height	Area %	Height %
1	14.252	19998793	300383	49.900	58.467
2	20.050	20079343	213384	50.100	41.533
Total		40078137	513766	100.000	100.000

Racemic **3u**

Chromatogram
JCH-661-OD C:\LabSolutions\Data\Project1\JCH-661-OD.lcd

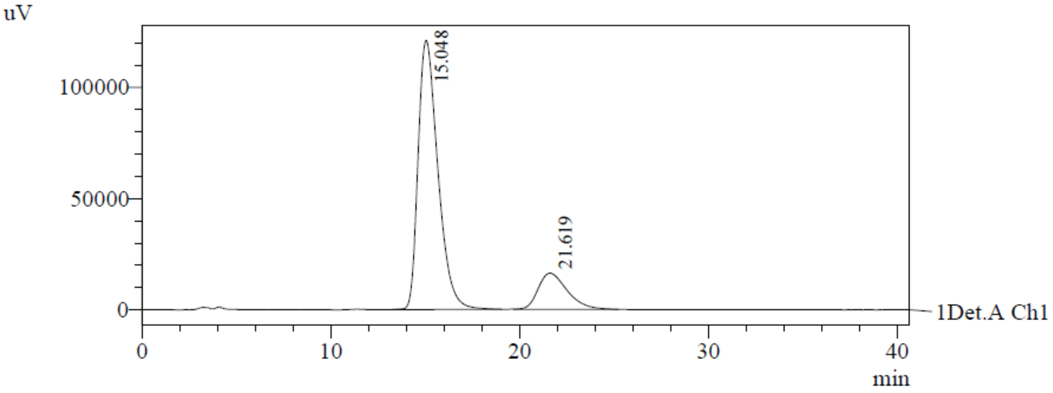

1 Det.A Ch1 / 254nm

PeakTable

Detector A Ch1 254nm

Peak#	Ret. Time	Area	Height	Area %	Height %
1	15.048	8819453	121093	83.594	88.183
2	21.619	1730828	16227	16.406	11.817
Total		10550281	137320	100.000	100.000

Enantiomerically enriched **3u**

(*R*)-4-Methoxy-*N*-(3-(2-methoxyphenyl)-3-oxo-1-phenylpropyl)benzenesulfonamide (**3v**)

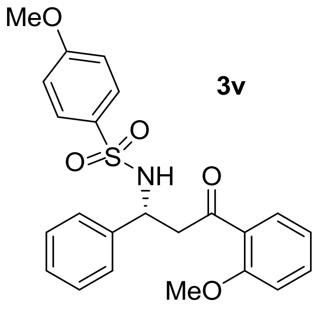

3v

A white solid; $[\alpha]^{25}_{D}$ = +32.4 (*c* 1.00, $CHCl_3$); ^{1}H NMR (500 MHz, $CDCl_3$) δ 16.48–7.67 (m, 3H), 16.48–7.51 (m, 6H), 7.45 (ddd, *J* = 8.5 Hz, 7.4 Hz, 1.8 Hz, 1H), 7.25–7.13 (m, 6H), 6.98–6.89 (m, 2H), 6.79 (d, *J* = 8.9 Hz, 2H), 5.71 (d, *J* = 6.8 Hz, 1H), 4.82 (q, *J* = 6.3 Hz, 1H), 3.87 (s, 3H), 3.80 (s, 3H), 3.46 (t, *J* = 5.9 Hz, 2H); ^{13}C NMR (126 MHz, $CDCl_3$) δ 199.52, 162.56, 158.70, 140.48, 134.21, 132.00, 130.56, 129.27, 128.36, 127.35, 127.23, 126.71, 120.72, 113.82, 111.48, 55.48, 54.59, 49.84; HRMS (ESI) *m*/*z*: calcd for $C_{23}H_{23}NO_5S$ $[M + Na]^+$ 448.1189, found 448.1179; The *ee* value was 60%, t_R (major) = 14.9 min, t_R (minor) = 17.4 min (Chiralcel OD-H, λ = 254 nm, 30% iPrOH/hexanes, flow rate = 1.0 mL/min).

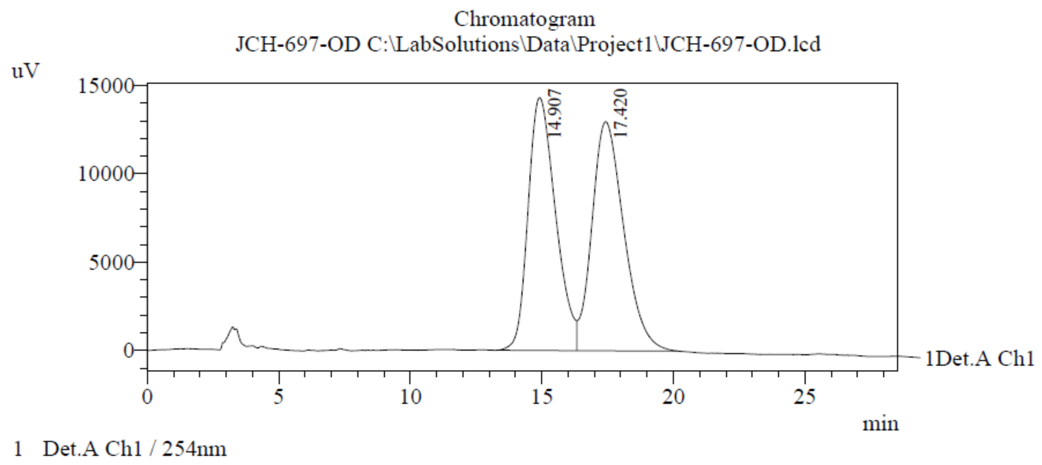

PeakTable

Detector A Ch1 254nm

Peak#	Ret. Time	Area	Height	Area %	Height %
1	14.907	1042494	14315	48.775	52.466
2	17.420	1094875	12970	51.225	47.534
Total		2137369	27285	100.000	100.000

Racemic **3v**

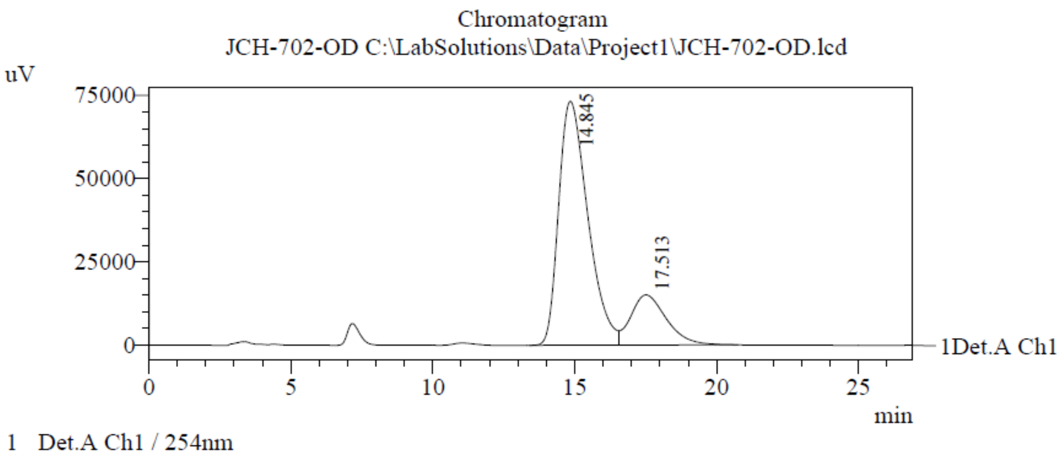

PeakTable

Detector A Ch1 254nm

Peak#	Ret. Time	Area	Height	Area %	Height %
1	14.845	5243341	73254	79.840	82.934
2	17.513	1323959	15074	20.160	17.066
Total		6567301	88327	100.000	100.000

Enantiomerically enriched **3v**

(R)-4-Methoxy-N-(3-oxo-1-phenylhexyl)benzenesulfonamide (**3w**)

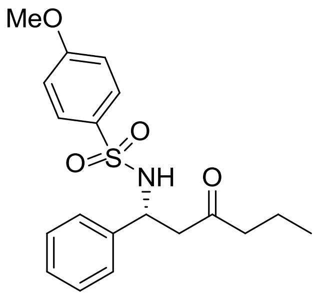

3w

A colorless oil; $[\alpha]^{25}_{D}$ = +38.4 (c 1.00, $CHCl_3$); ^{1}H NMR (500 MHz, $CDCl_3$) δ 7.63 (d, J = 8.9 Hz, 2H), 7.17 (t, J = 5.8 Hz, 3H), 7.11–7.03 (m, 2H), 6.86–6.80 (m, 2H), 5.68 (d, J = 7.1 Hz, 1H), 4.67 (dd, J = 12.8 Hz, 6.1 Hz, 1H), 3.83 (s, 3H), 2.98 (dd, J = 17.0 Hz, 5.6 Hz, 1H), 2.86 (dd, J = 17.0 Hz, 6.2 Hz, 1H), 2.22 (t, J = 7.3 Hz, 2H), 1.53–1.35 (m, 2H), 0.79 (t, J = 7.4 Hz, 3H); ^{13}C NMR (126 MHz, $CDCl_3$) δ 209.17, 162.70, 139.76, 131.85, 129.25, 128.50, 127.60, 126.55, 113.95, 55.55, 54.17, 48.56, 45.50, 16.76, 13.46; HRMS (ESI) m/z: calcd for $C_{19}H_{23}NO_4S$ $[M + Na]^+$ 384.1240, found 384.1236; The ee value was 54%, t_R (major) = 9.00 min, t_R (minor) = 11.8 min (Chiralcel OD-H, λ = 254 nm, 30% iPrOH/hexanes, flow rate = 1.0 mL/min).

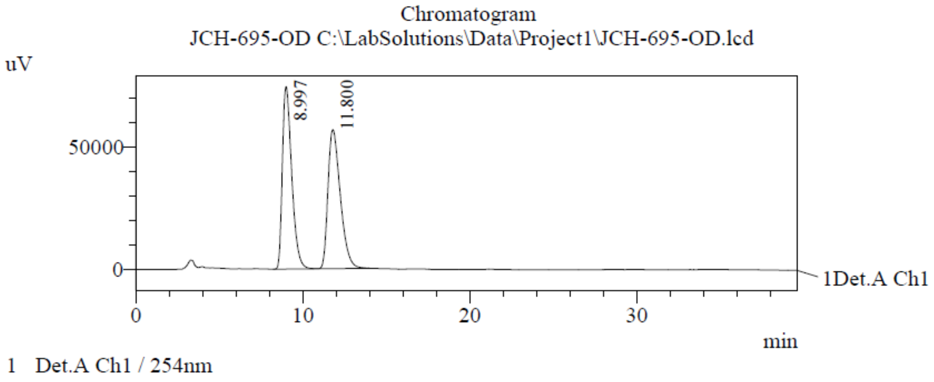

PeakTable

Detector A Ch1 254nm

Peak#	Ret. Time	Area	Height	Area %	Height %
1	8.997	2903490	74601	49.846	56.761
2	11.800	2921412	56828	50.154	43.239
Total		5824902	131428	100.000	100.000

Racemic **3w**

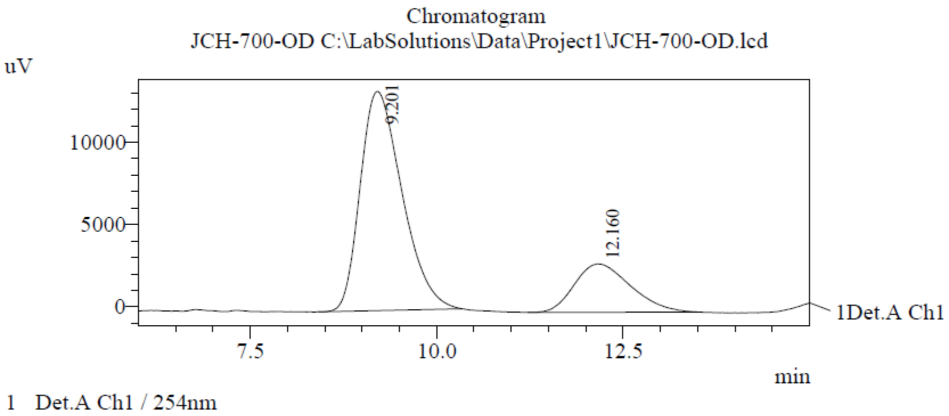

PeakTable

Detector A Ch1 254nm

Peak#	Ret. Time	Area	Height	Area %	Height %
1	9.201	519648	13305	77.125	81.933
2	12.160	154126	2934	22.875	18.067
Total		673774	16239	100.000	100.000

Enantiomerically enriched **3w**

(*R*)-*N*-(4,4-Dimethyl-3-oxo-1-phenylpentyl)-4-methoxybenzenesulfonamide (**3x**)

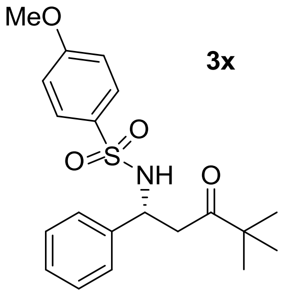

3x

A colorless oil; $[\alpha]^{25}_{D}$ = +32.2 (*c* 1.00, $CHCl_3$); ^{1}H NMR (500 MHz, $CDCl_3$) δ 7.64 (d, *J* = 8.9 Hz, 2H), 7.21–7.12 (m, 3H), 7.07 (dd, *J* = 7.5 Hz, 1.9 Hz, 2H), 6.84 (d, *J* = 8.9 Hz, 2H), 5.77 (d, *J* = 6.9 Hz, 1H), 4.67 (dd, *J* = 12.4 Hz, 6.0 Hz, 1H), 3.83 (s, 3H), 3.08 (dd, *J* = 17.4 Hz, 5.3 Hz, 1H), 2.95 (dd, *J* = 17.4 Hz, 6.1 Hz, 1H), 0.94 (s, 9H); ^{13}C NMR (126 MHz, $CDCl_3$) δ 214.21, 162.71, 139.88, 131.94, 129.29, 128.43, 127.54, 126.58, 113.96, 55.56, 54.44, 44.31, 42.93, 25.74; HRMS (ESI) *m*/*z*: calcd for $C_{20}H_{25}NO_4S$ $[M + Na]^+$ 398.1397, found 398.1395; The *ee* value was 73%, t_R (major) = 7.1 min, t_R (minor) = 11.0 min (Chiralcel OD-H, λ = 254 nm, 30% iPrOH/hexanes, flow rate = 1.0 mL/min).

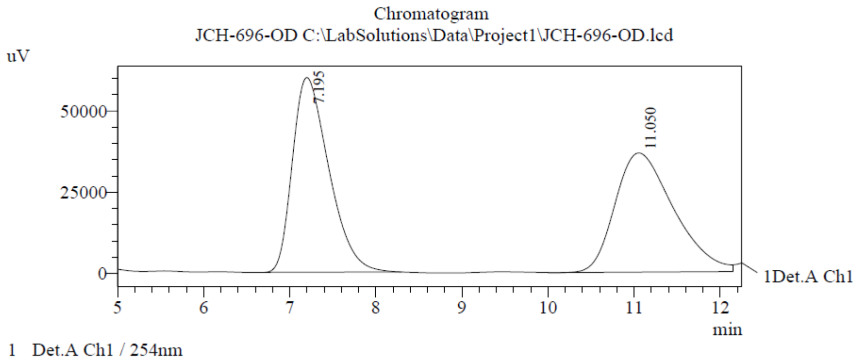

PeakTable

Detector A Ch1 254nm

Peak#	Ret. Time	Area	Height	Area %	Height %
1	7.195	1764841	59940	50.376	62.036
2	11.050	1738476	36681	49.624	37.964
Total		3503317	96621	100.000	100.000

Racemic **3x**

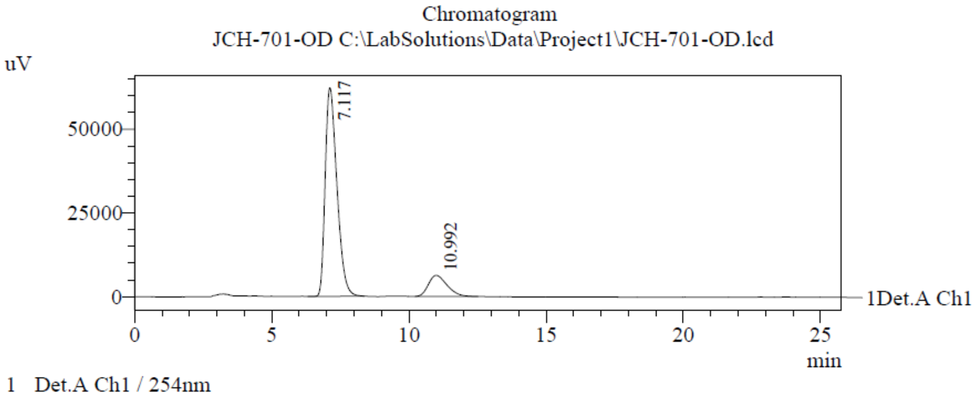

PeakTable

Detector A Ch1 254nm

Peak#	Ret. Time	Area	Height	Area %	Height %
1	7.117	1853370	62284	86.189	90.821
2	10.992	296982	6295	13.811	9.179
Total		2150352	68579	100.000	100.000

Enantiomerically enriched **3x**

References:

1. Hayashi, T.; Ishigedani, M. *J. Am. Chem. Soc.* **2000**, *122*, 976–977.

2. Chemla, F.; Hebbe, V.; Normant, J.-F. *Synthesis* **2000**, 75–77.

3. Evans, D. A.; Mito, S.; Seidel, D. *J. Am. Chem. Soc.* **2007**, *129*, 11583–11592.

4. Zhao, C-H.; Liu, L.; Wang, D.; Chen, Y-J. *Eur. J. Org. Chem.* **2006,** 2977–2986.

E. NMR spectra

^{1}H NMR spectrum of **3a**

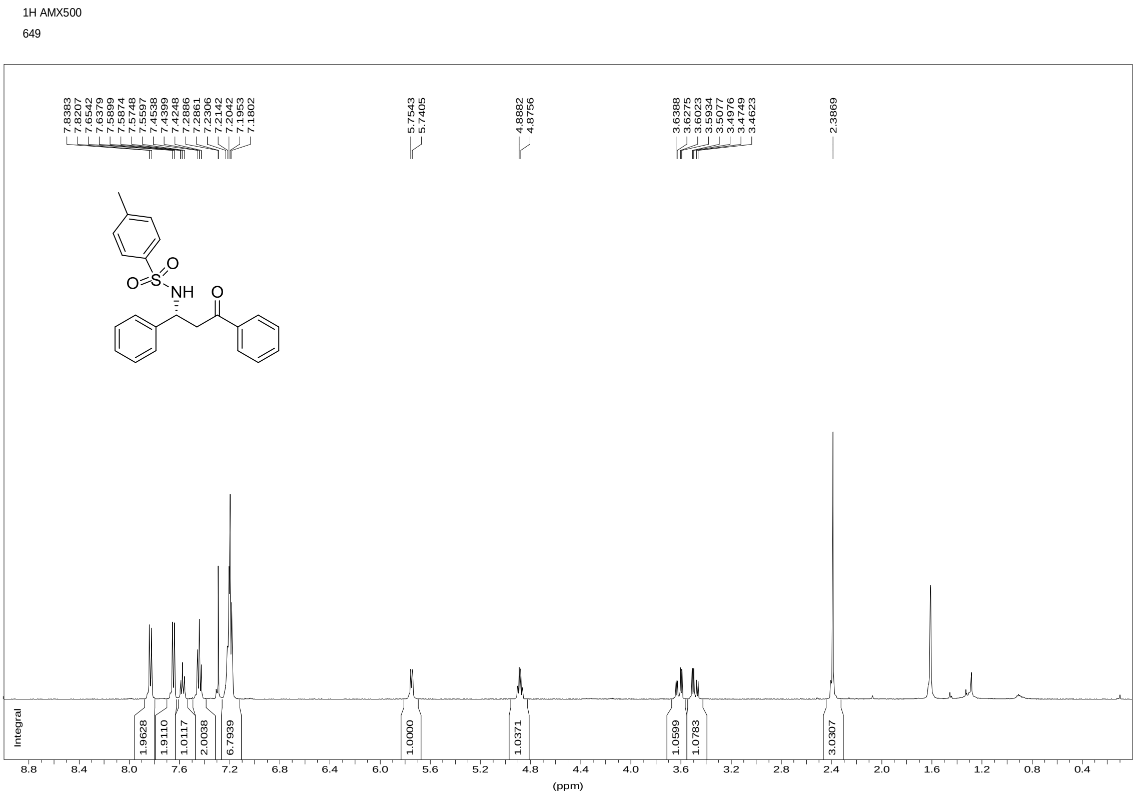

^{1}H and ^{13}C NMR spectra of **3c**

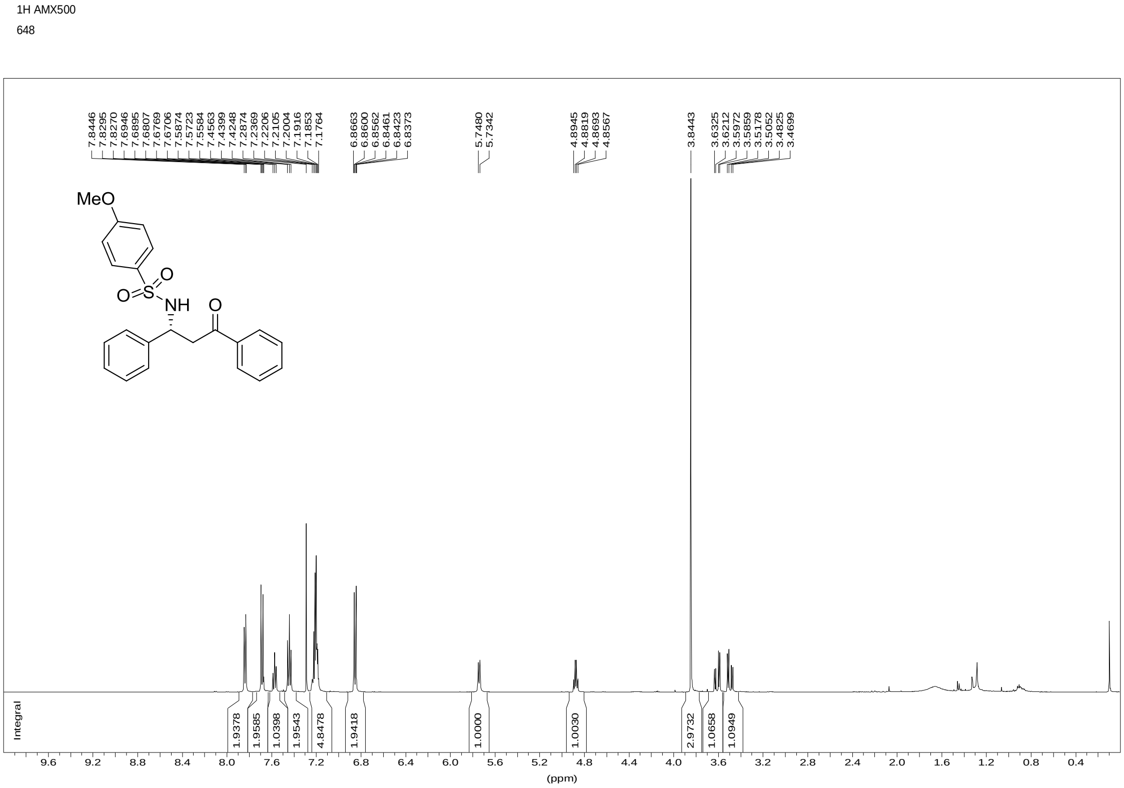

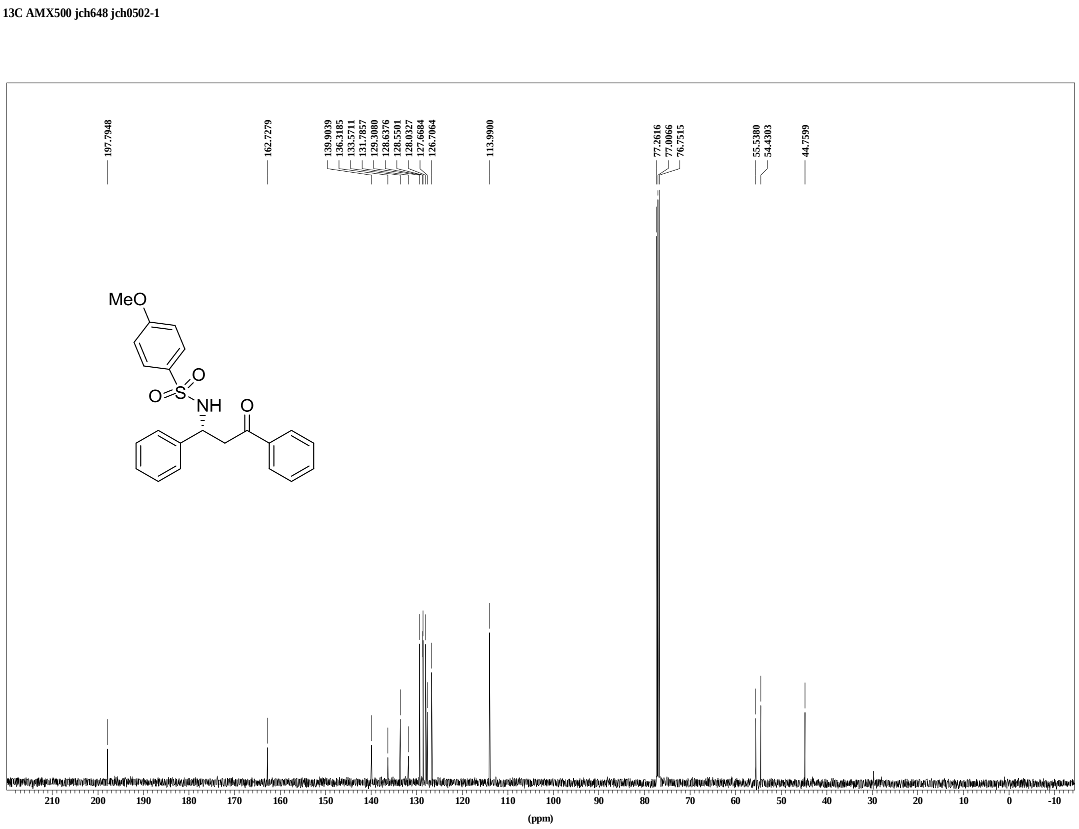

^{1}H and ^{13}C NMR spectra of **3g**

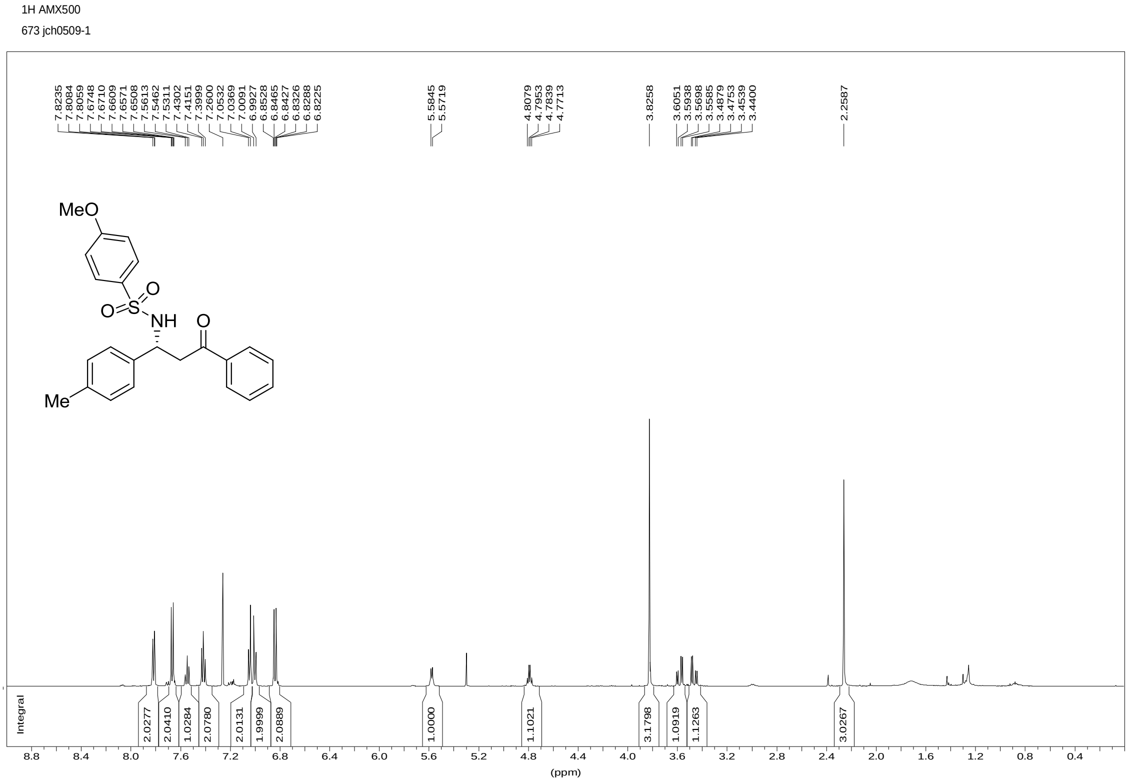

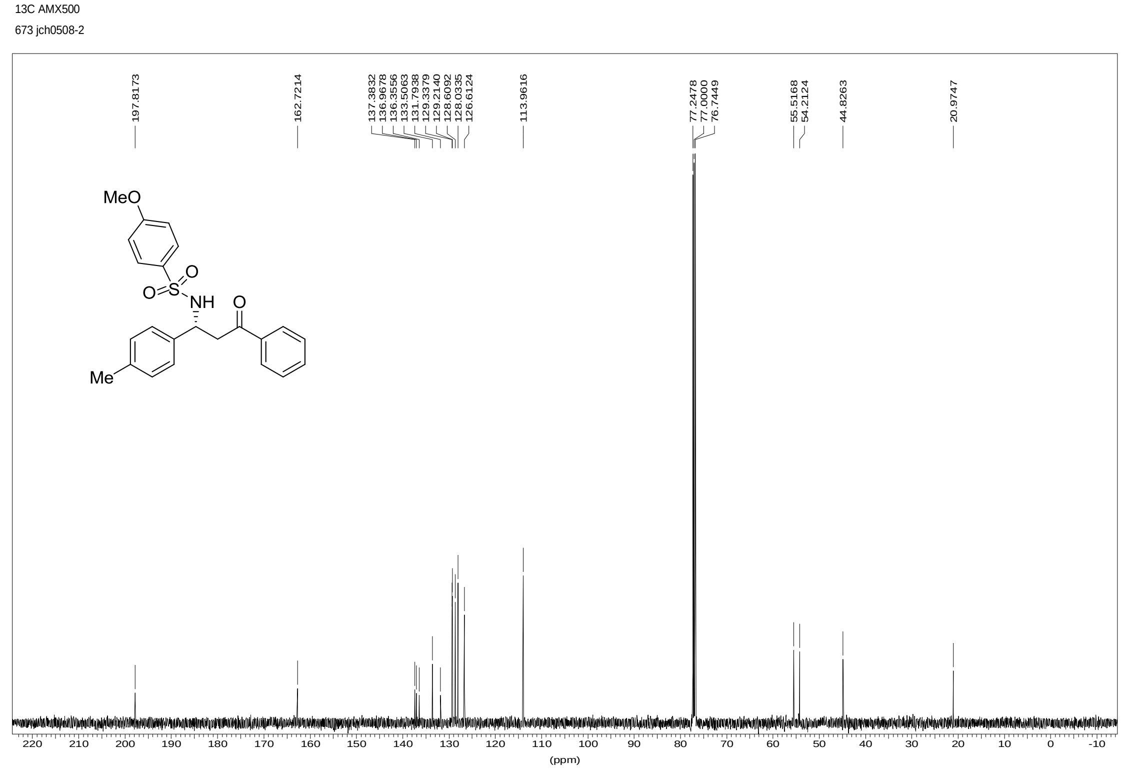

^{1}H and ^{13}C NMR spectra of **3h**

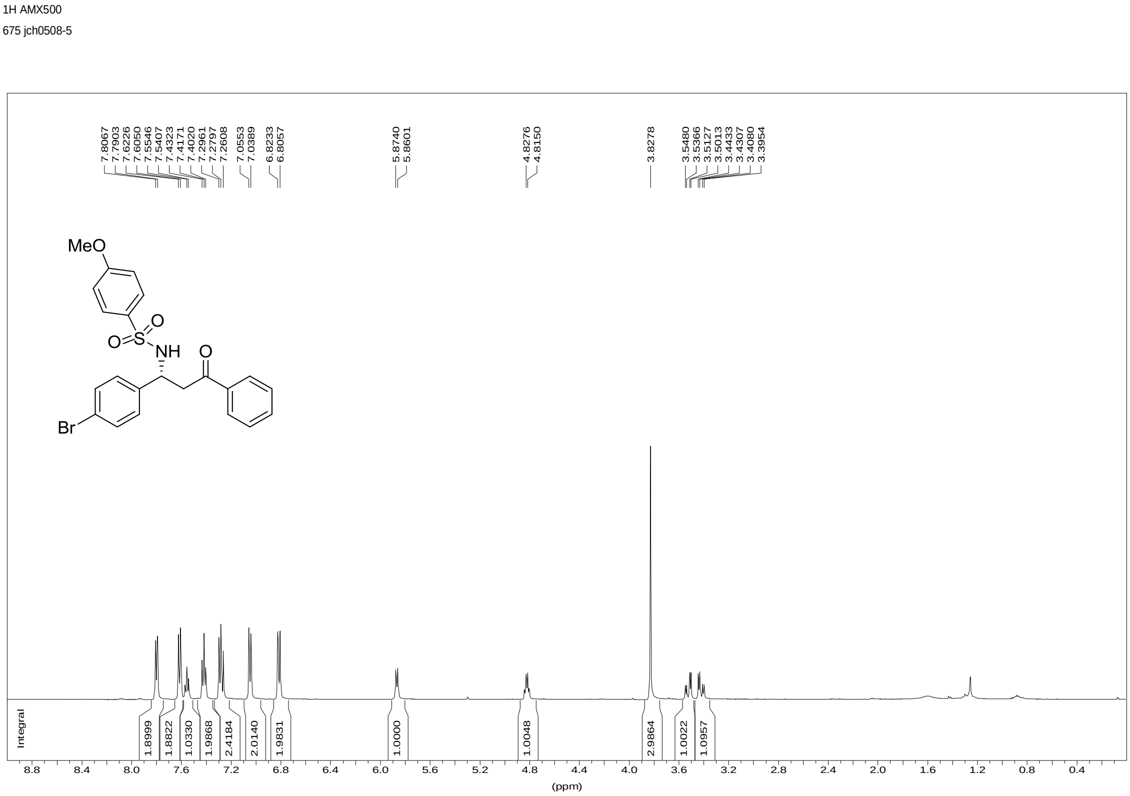

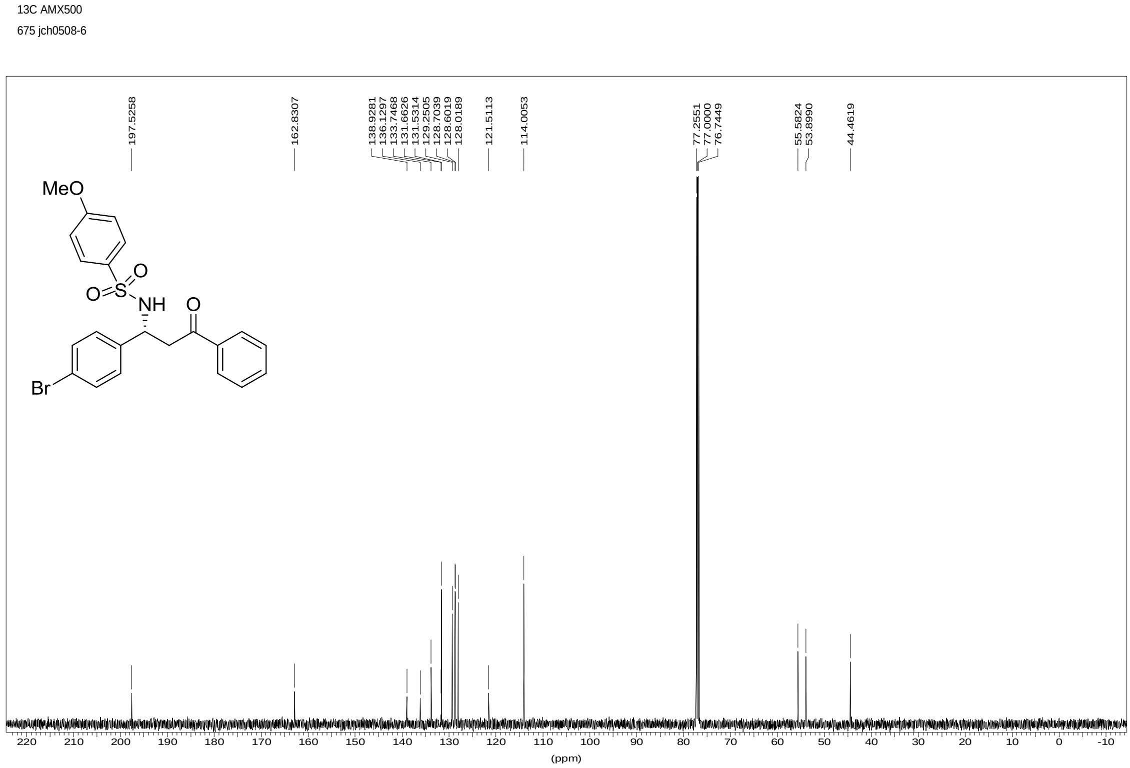

<u>^{1}H and ^{13}C NMR spectra of **3i**</u>

1H AMX500
676 jch0508-7

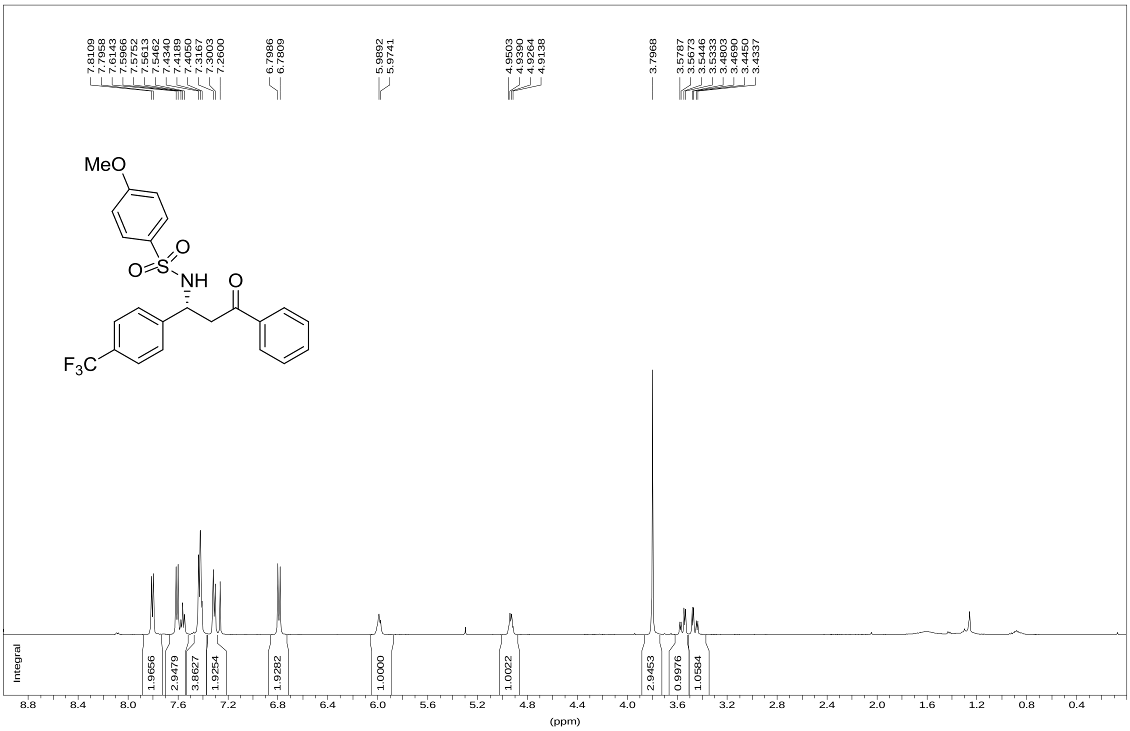

13C AMX500
676 jch0508-8

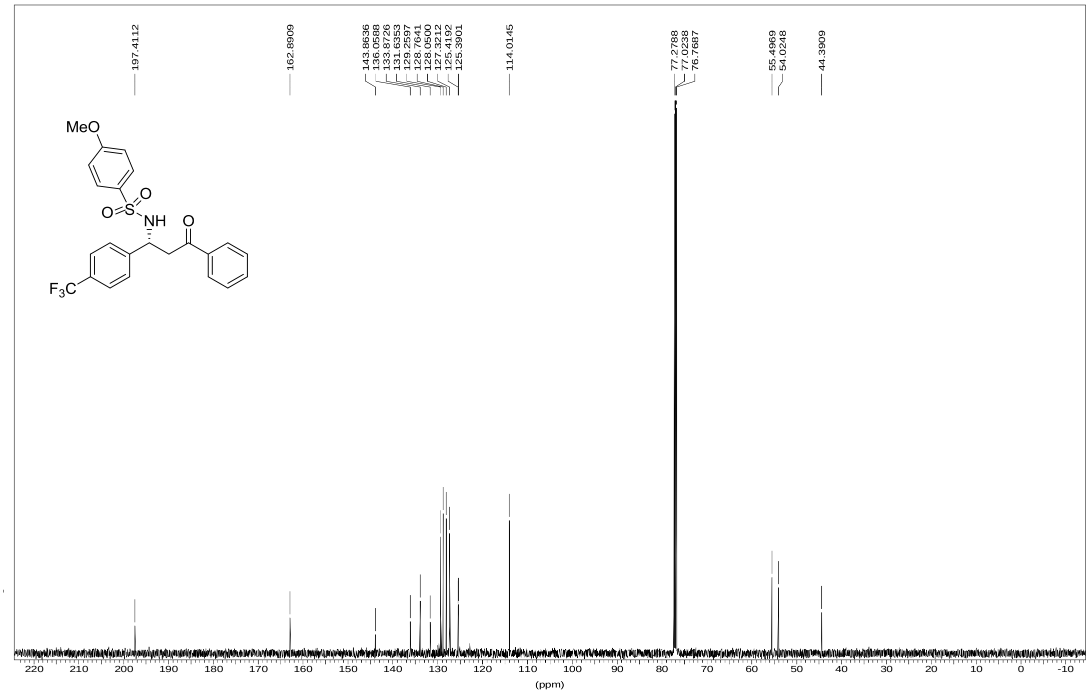

^{1}H and ^{13}C NMR spectra of **3j**

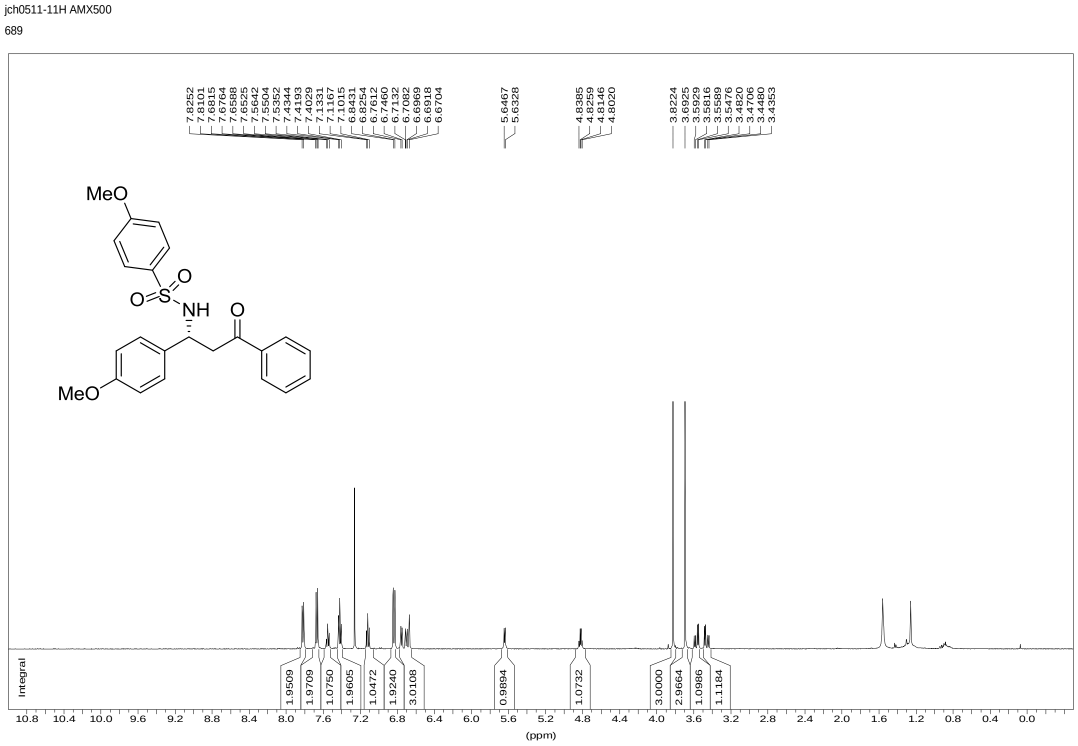

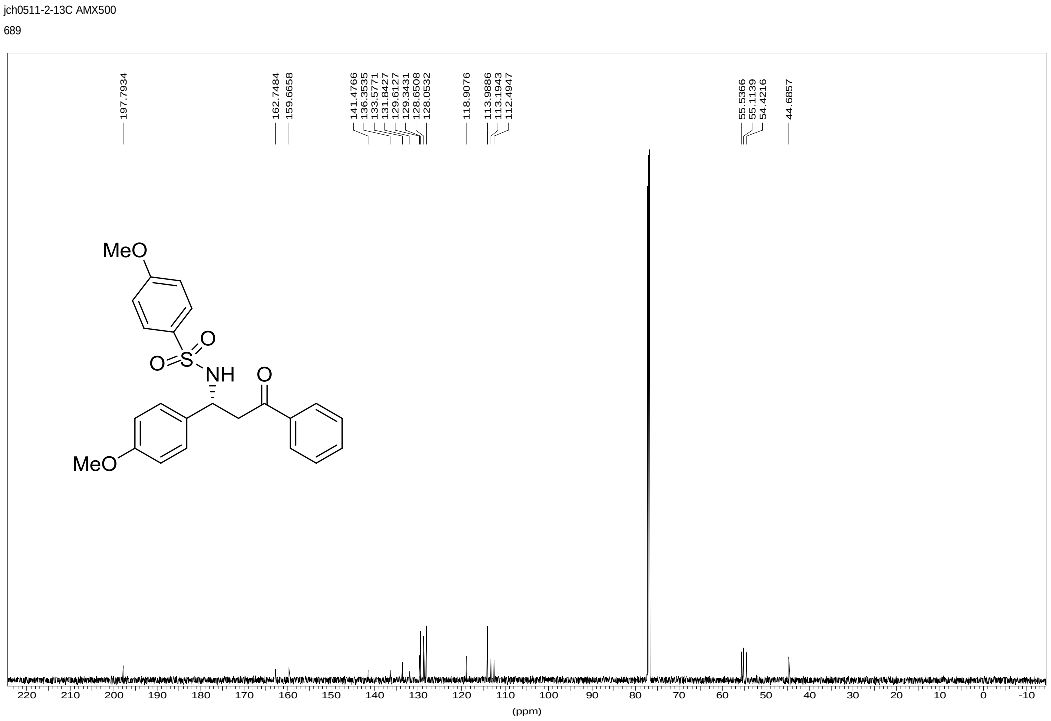

^{1}H and ^{13}C NMR spectra of **3k**

1H AMX500
678 jch0509-4

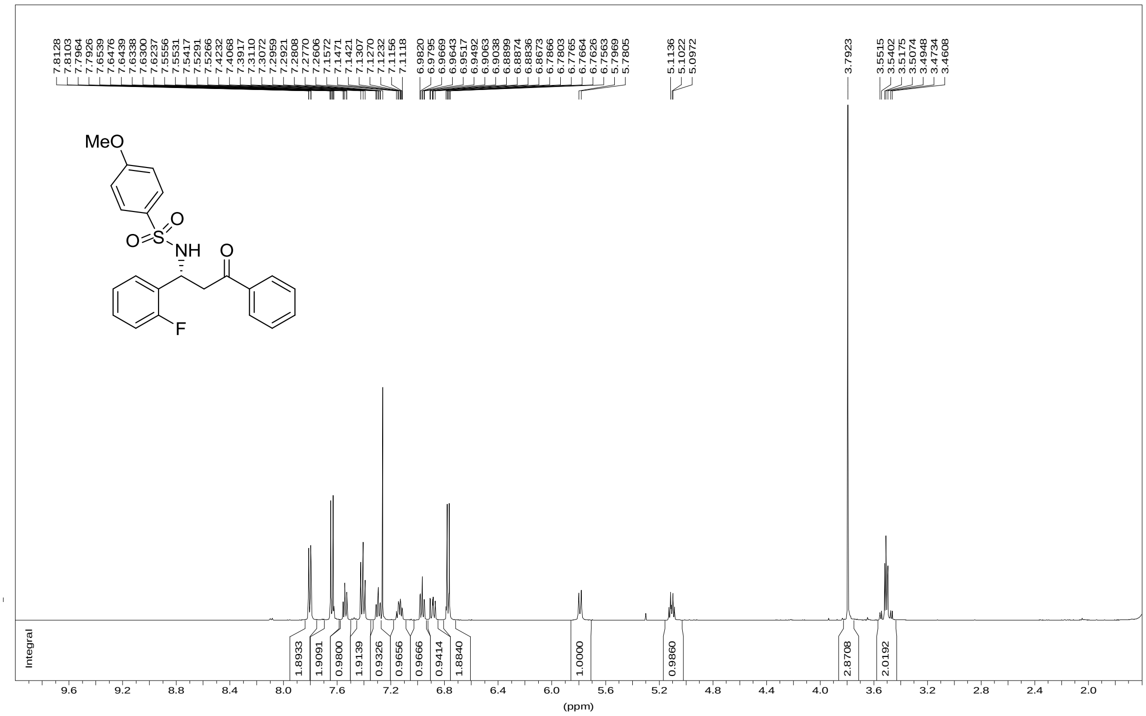

13C AMX500
678 jch0509-1-1

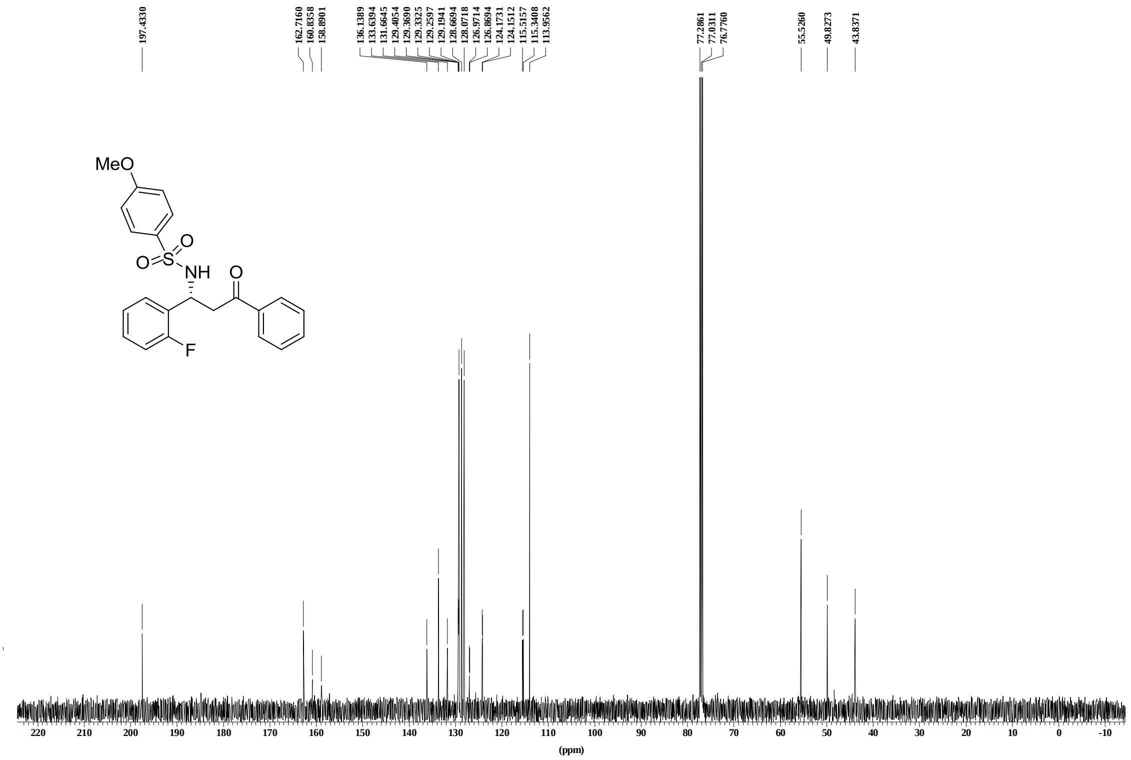

^{1}H and ^{13}C NMR spectra of **3l**

jch05-11-111H AMX500
702

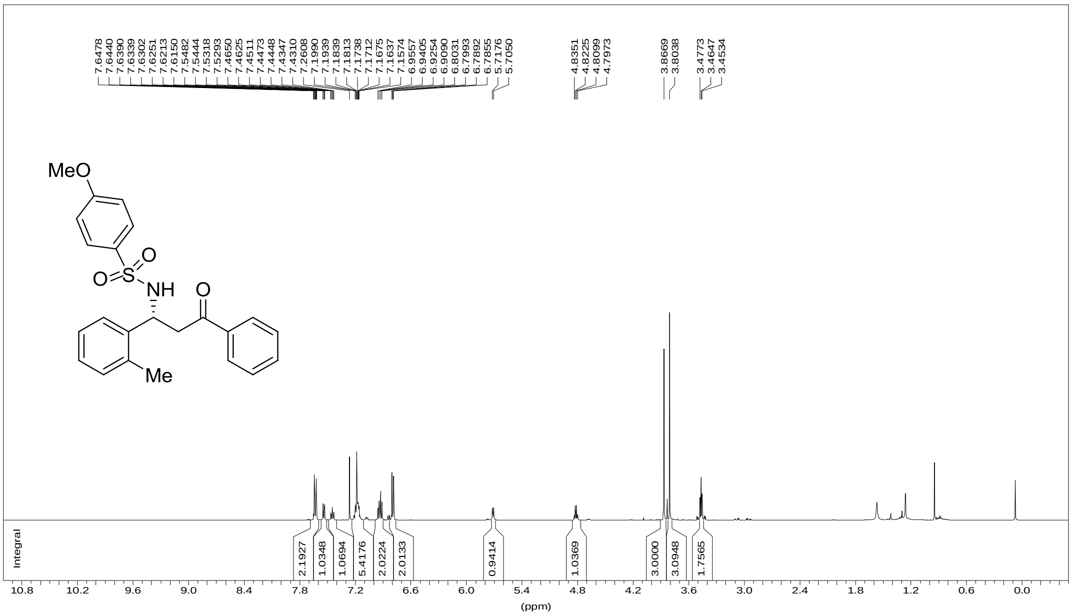

jch0510-4 13C AMX500
683

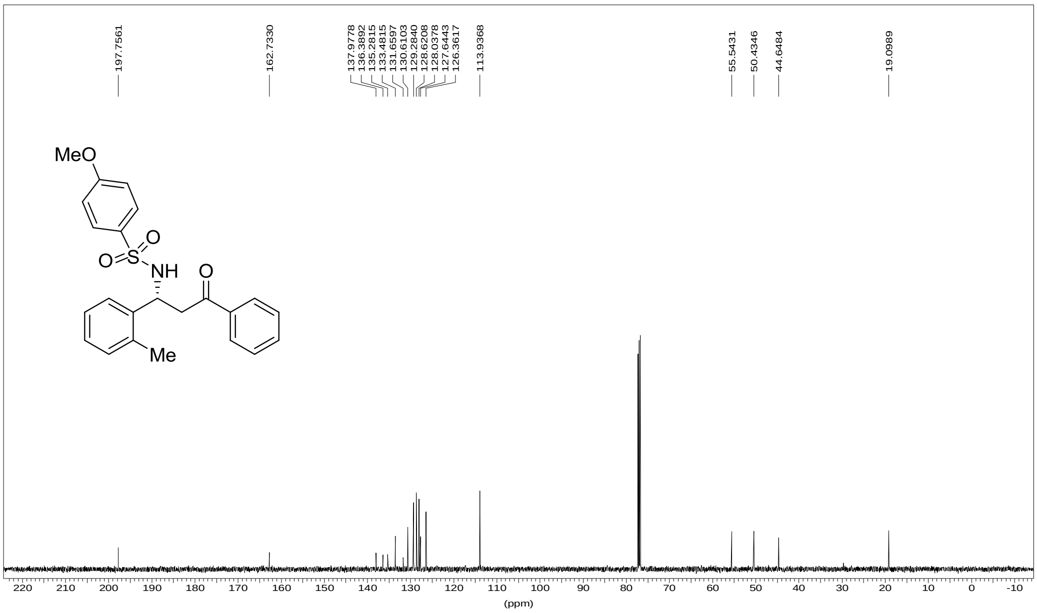

^1H and ^{13}C NMR spectra of **3m**

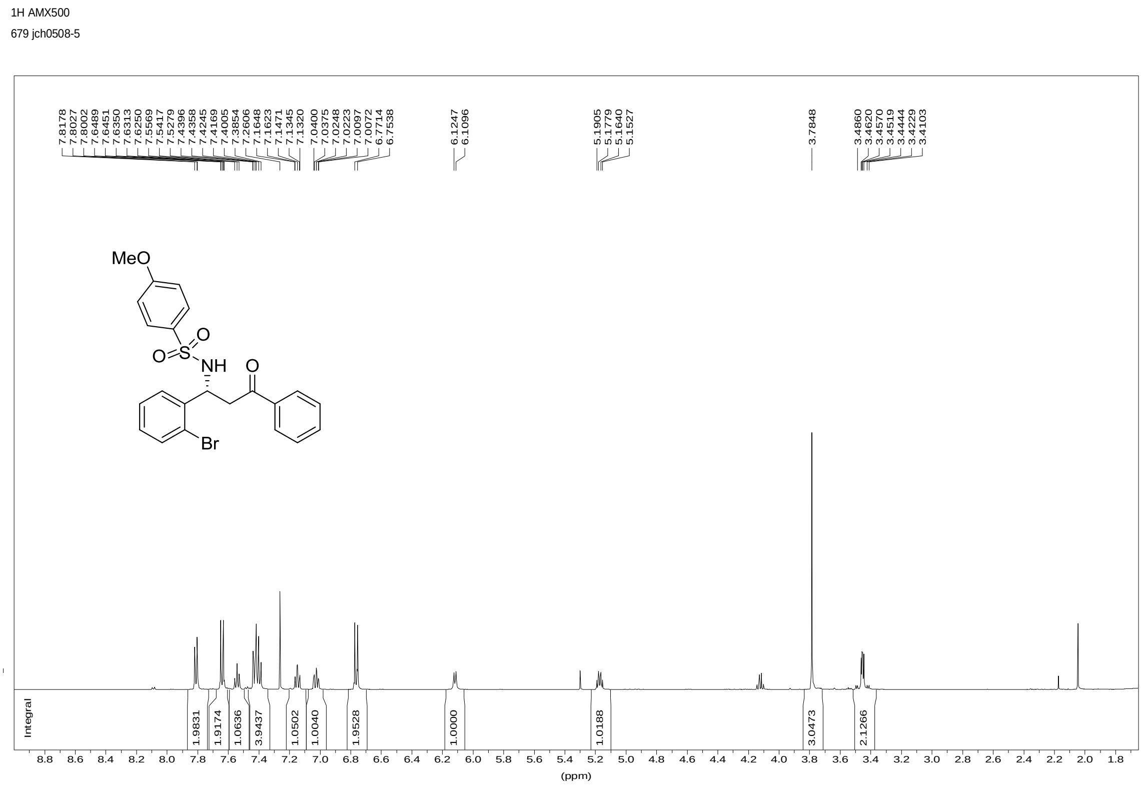

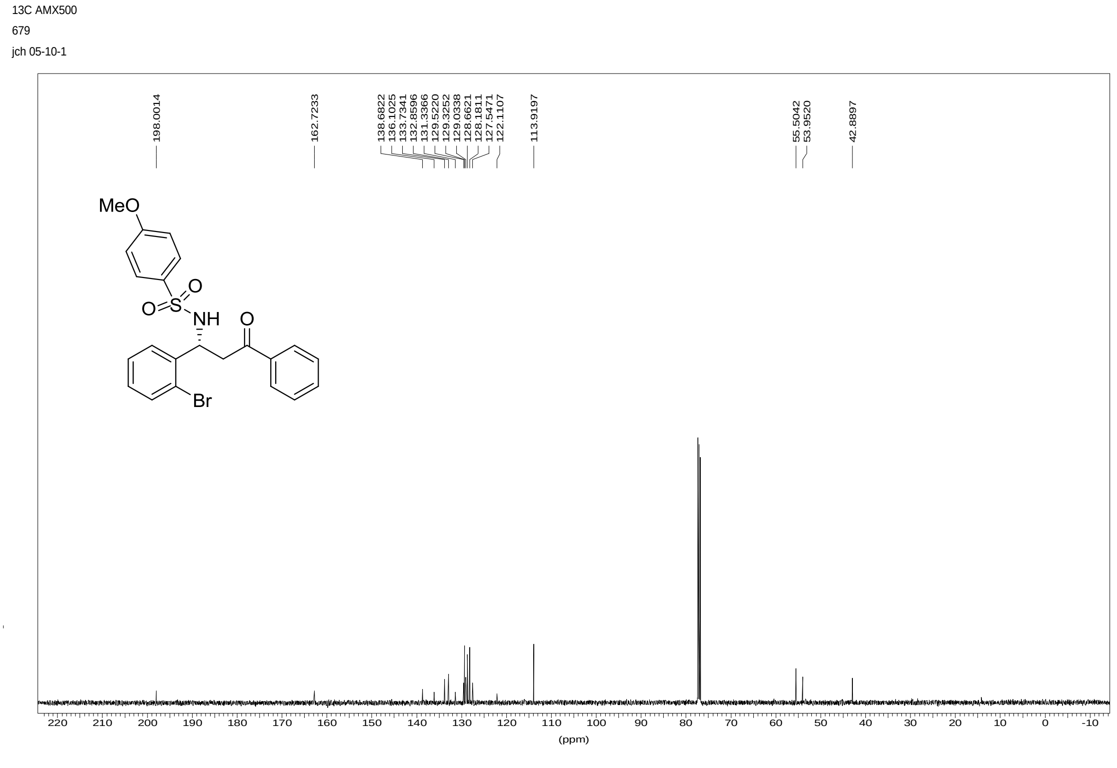

__^{1}H and ^{13}C NMR spectra of **3n**__

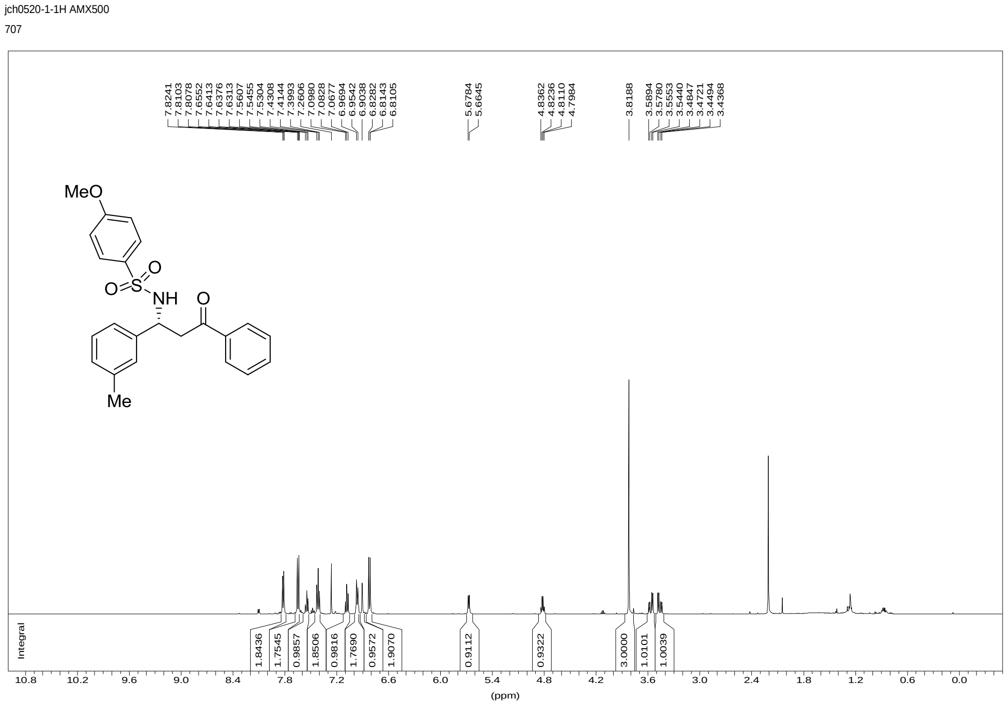

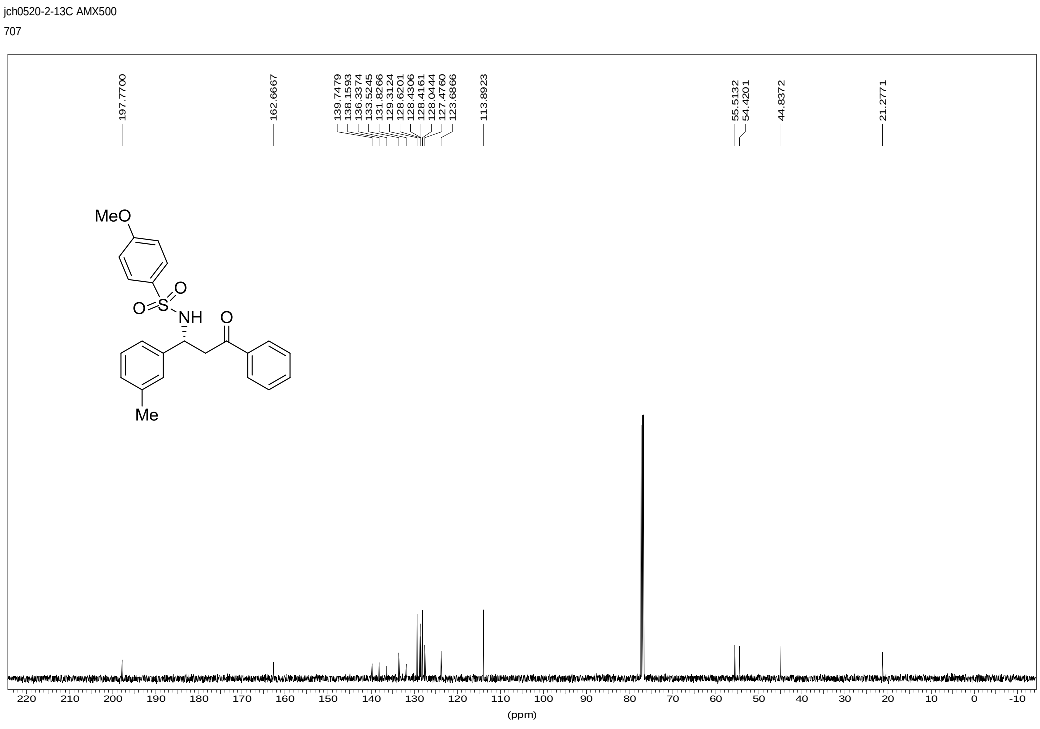

^1H and ^{13}C NMR spectra of **3o**

jch0520-3-1H AMX500
708

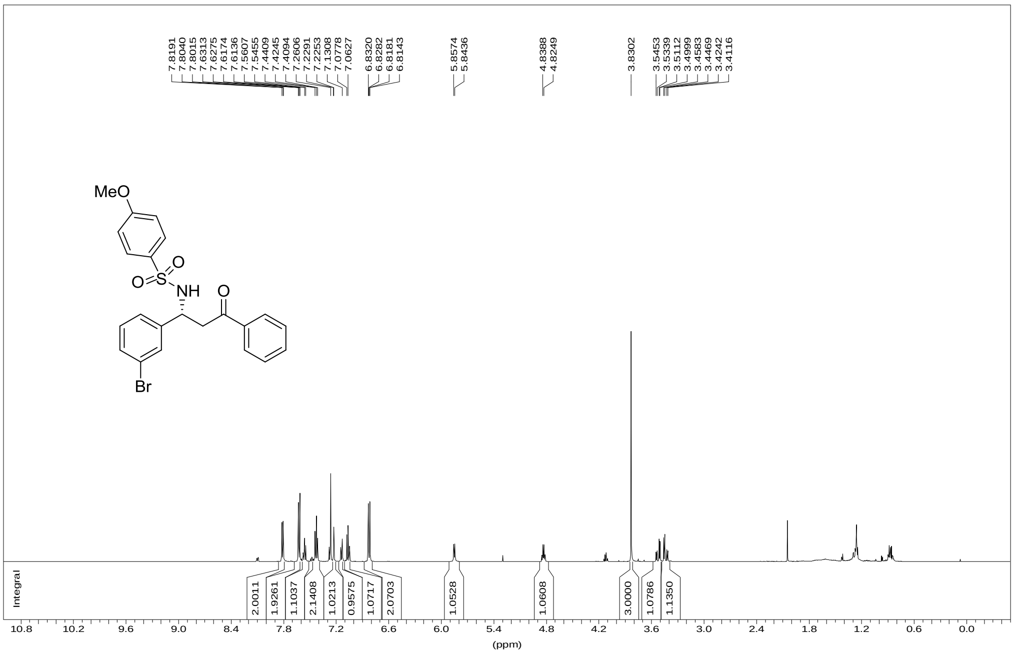

jch05-20-4-13C AMX500
708

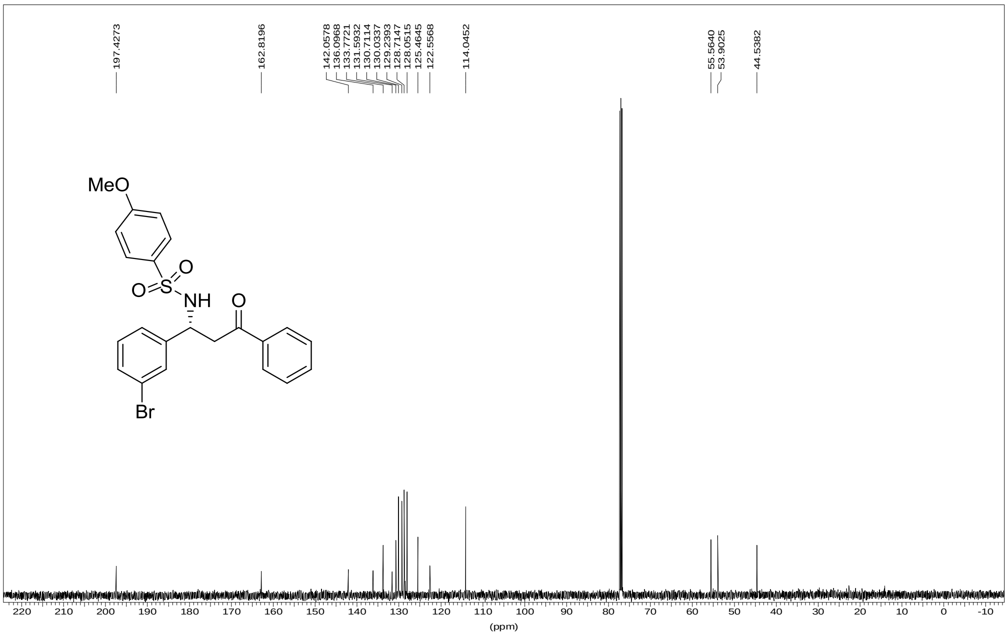

^{1}H and ^{13}C NMR spectra of **3p**

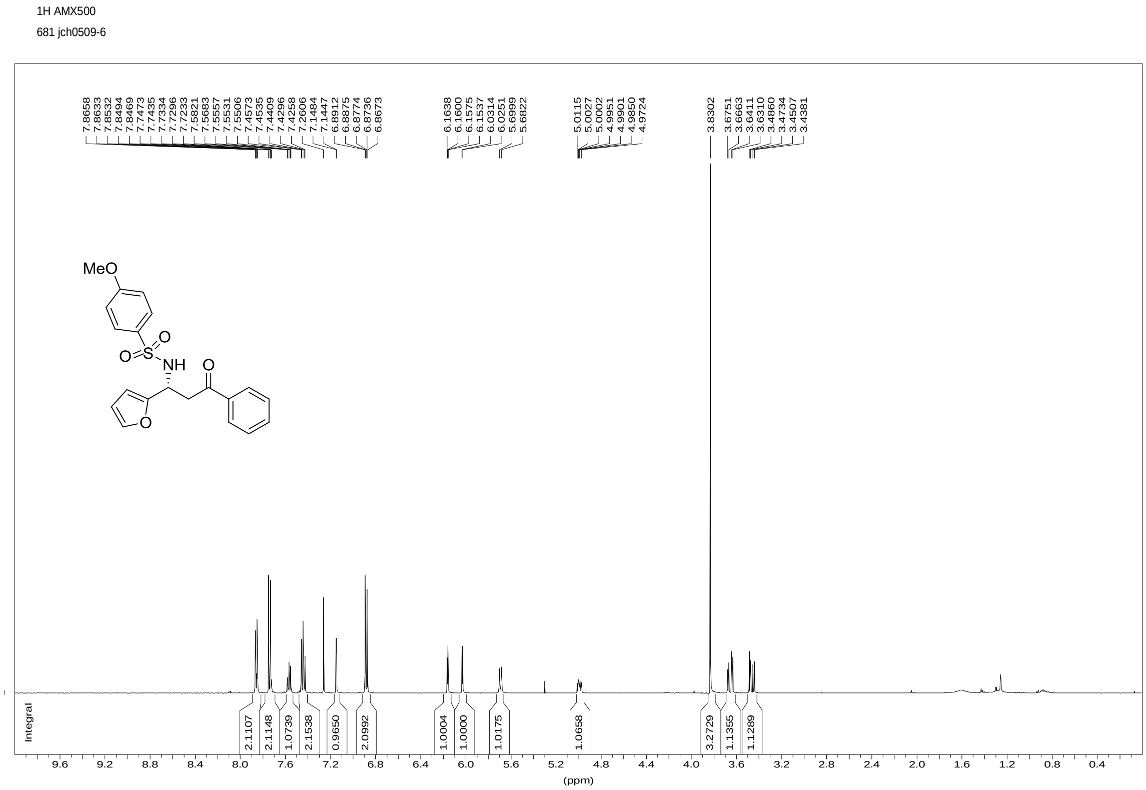

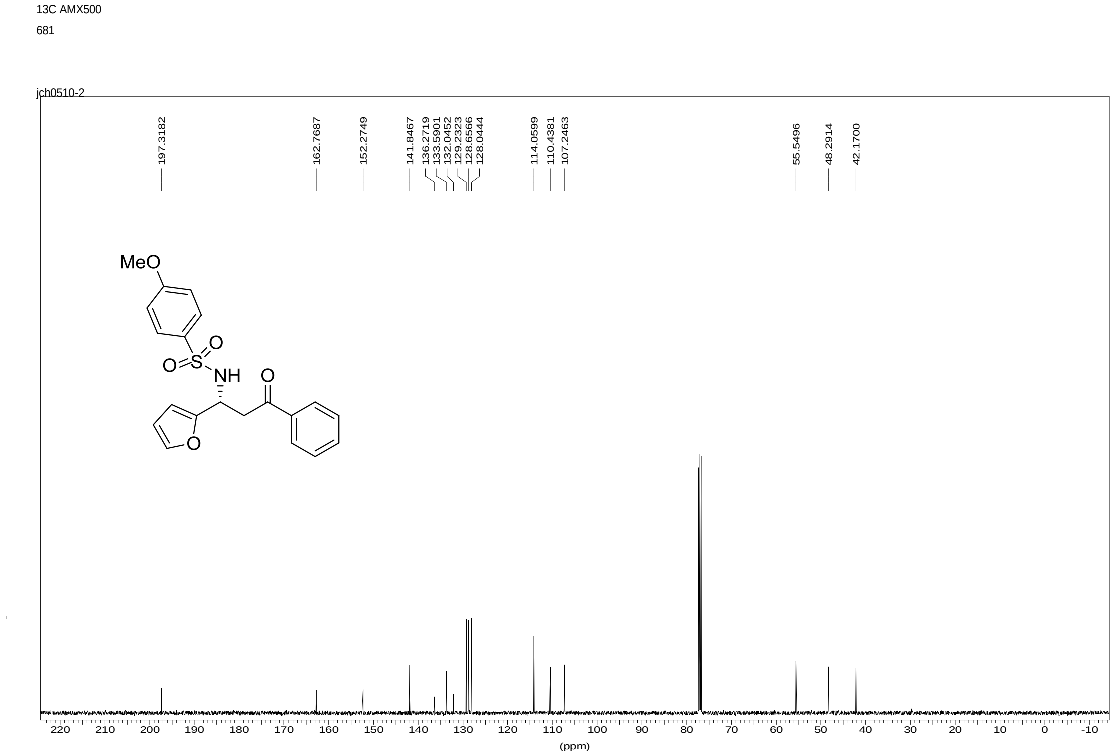

<u>^{1}H and ^{13}C NMR spectra of **3q**</u>

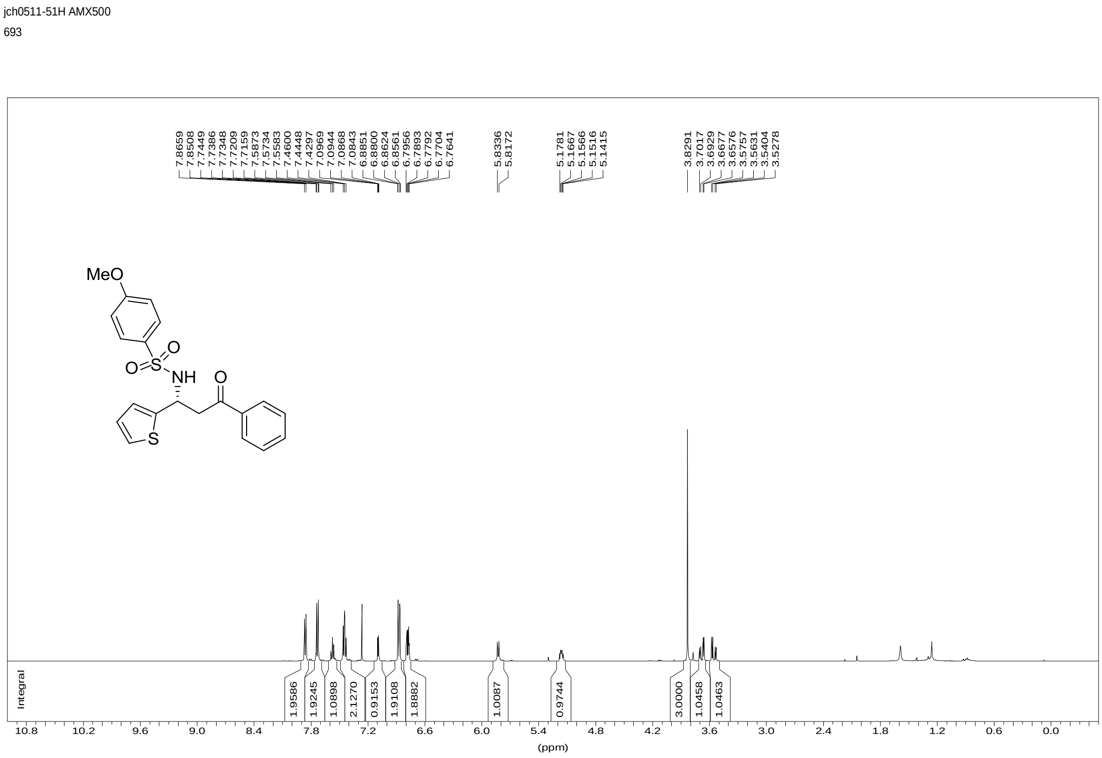

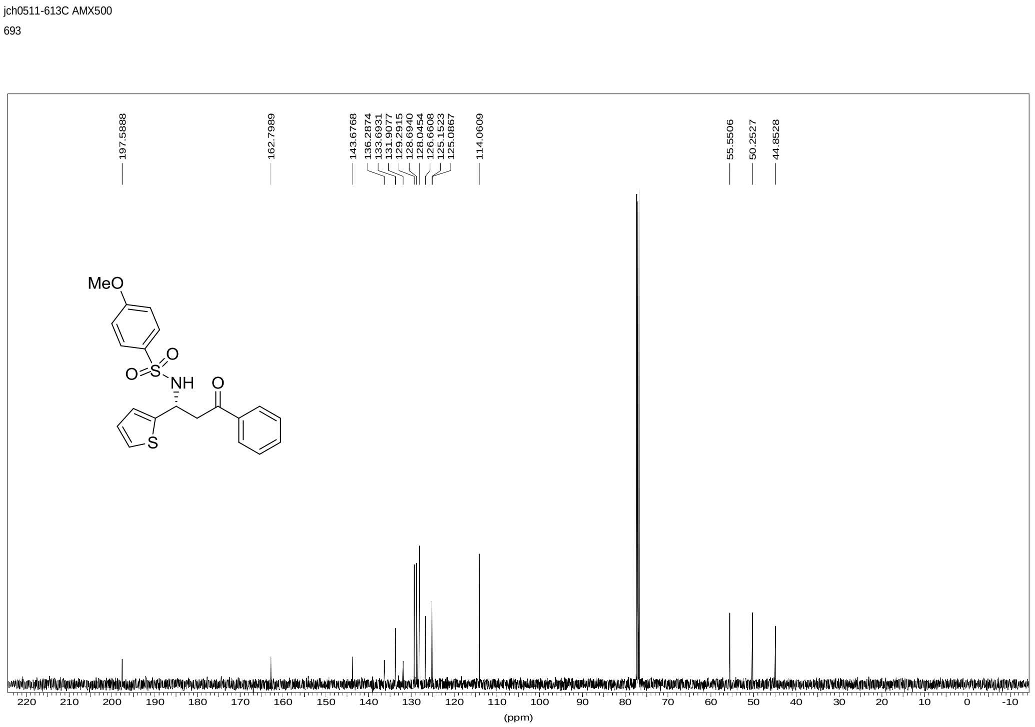

^{1}H and ^{13}C NMR spectra of **3r**

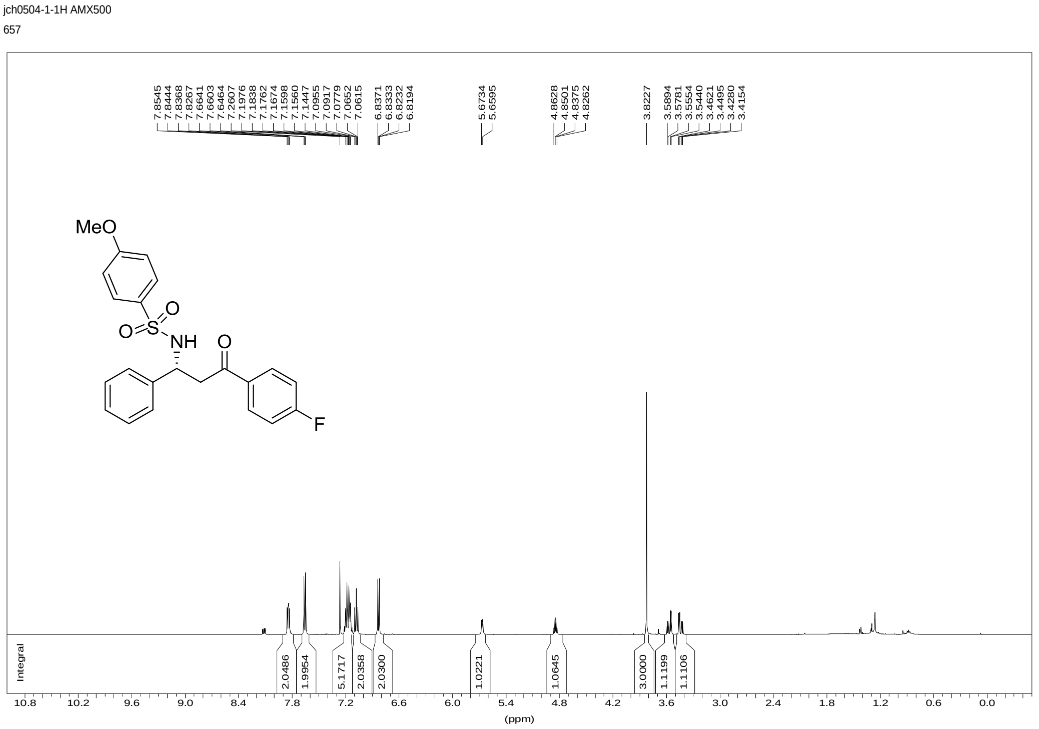

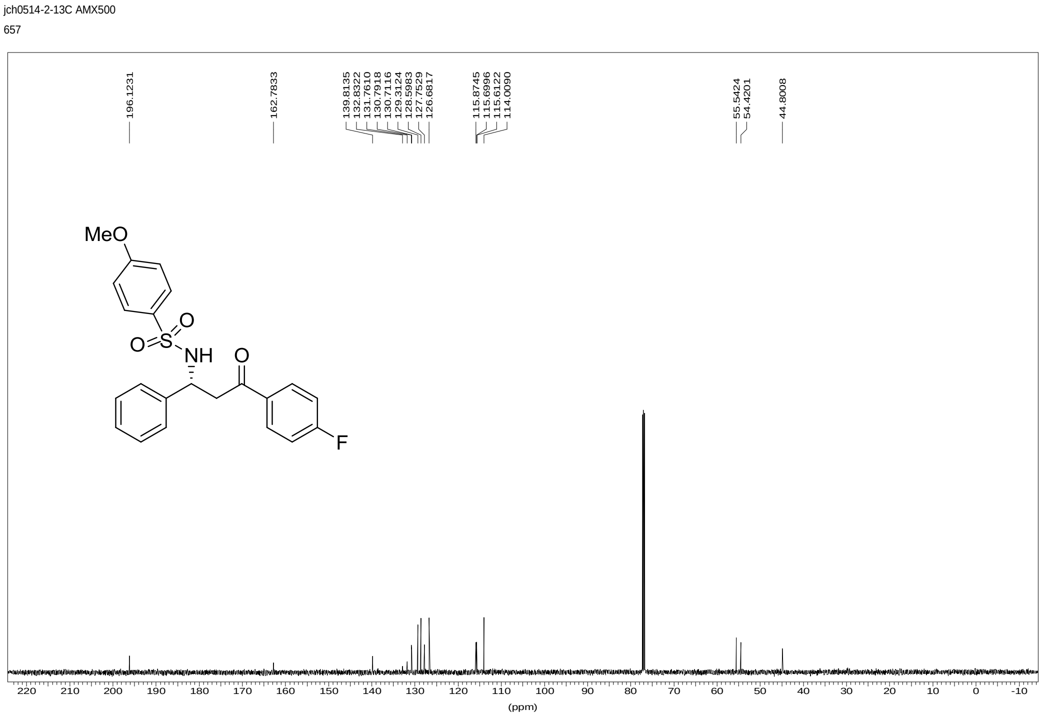

^{1}H and ^{13}C NMR spectra of **3s**

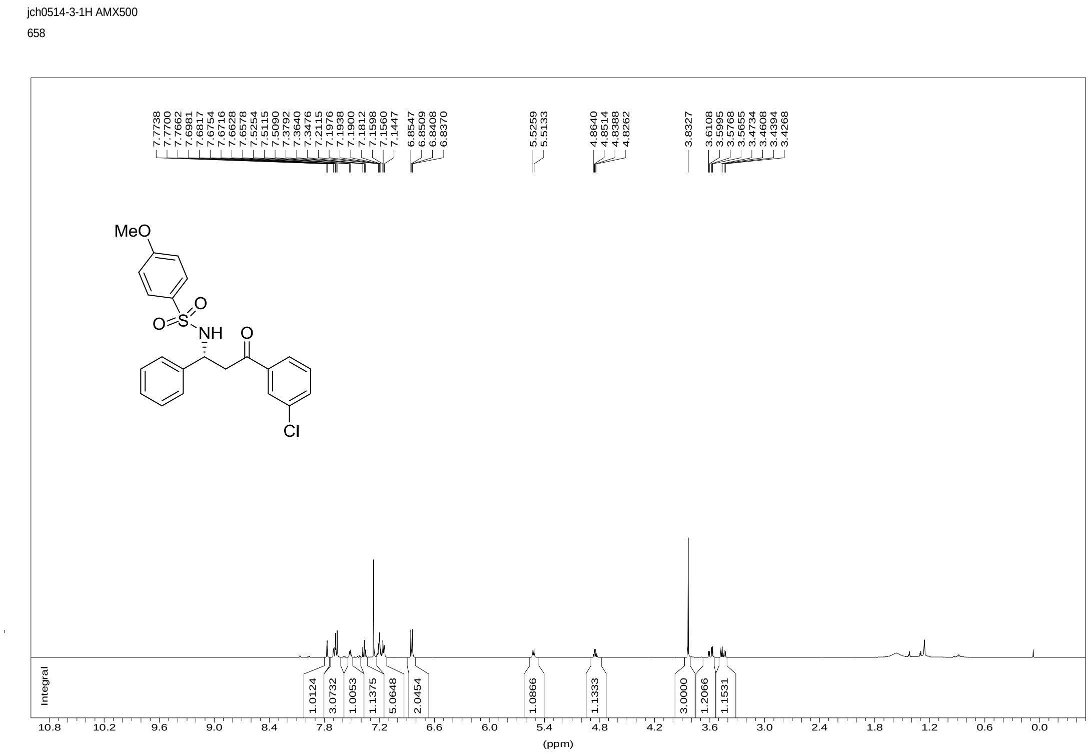

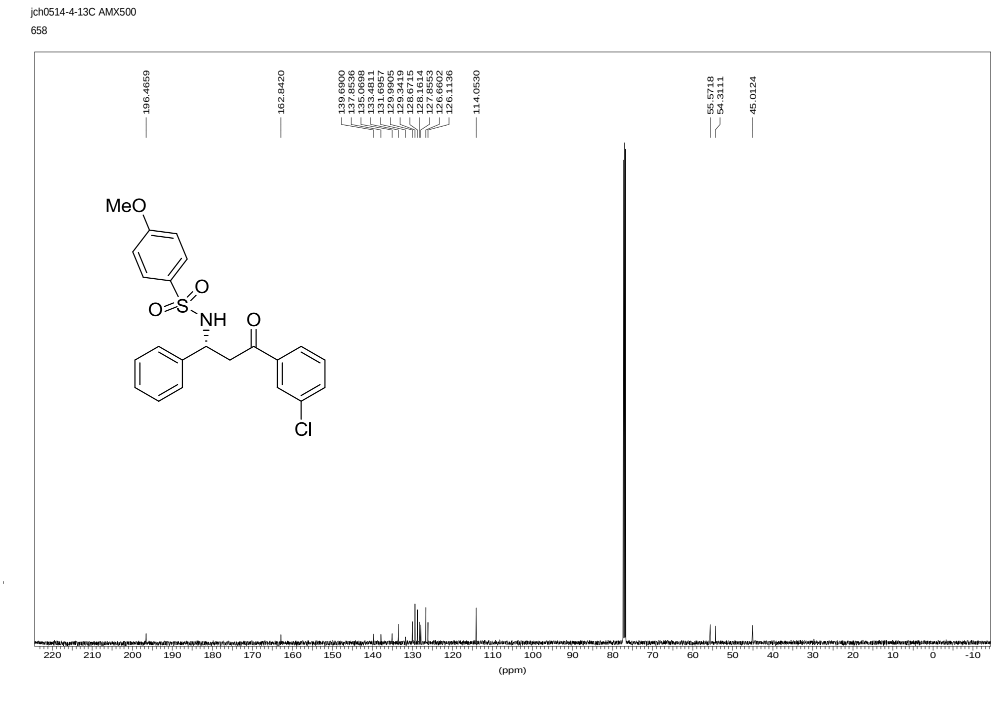

^{1}H and ^{13}C NMR spectra of **3t**

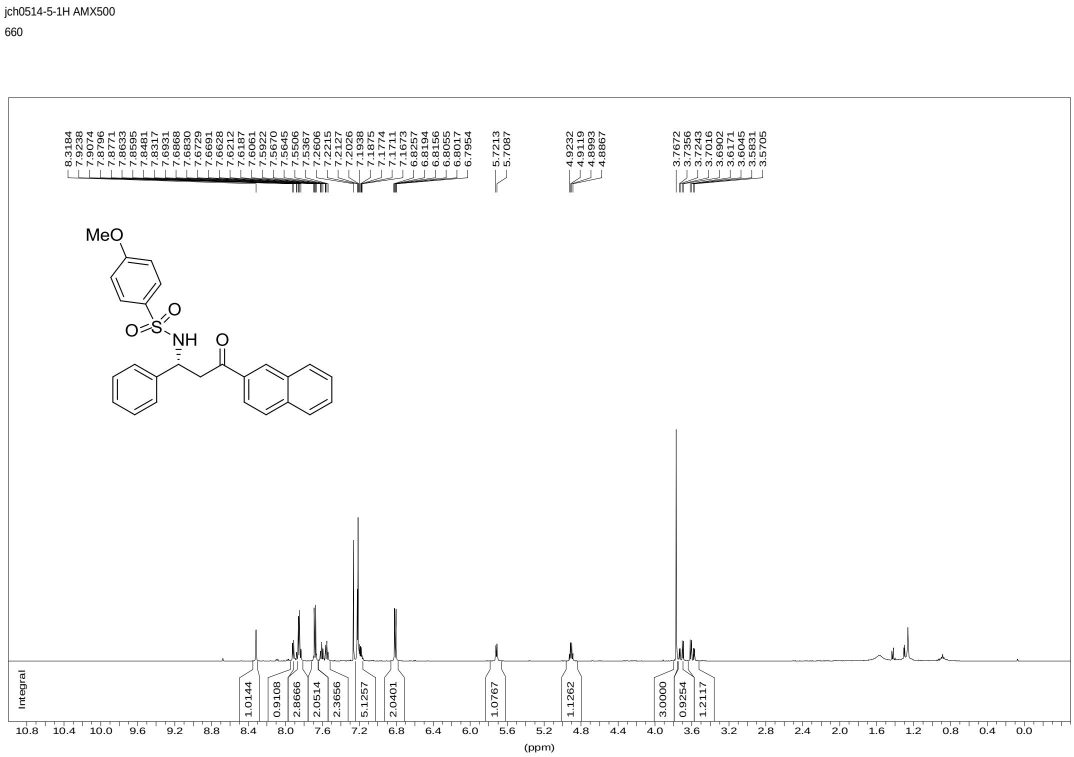

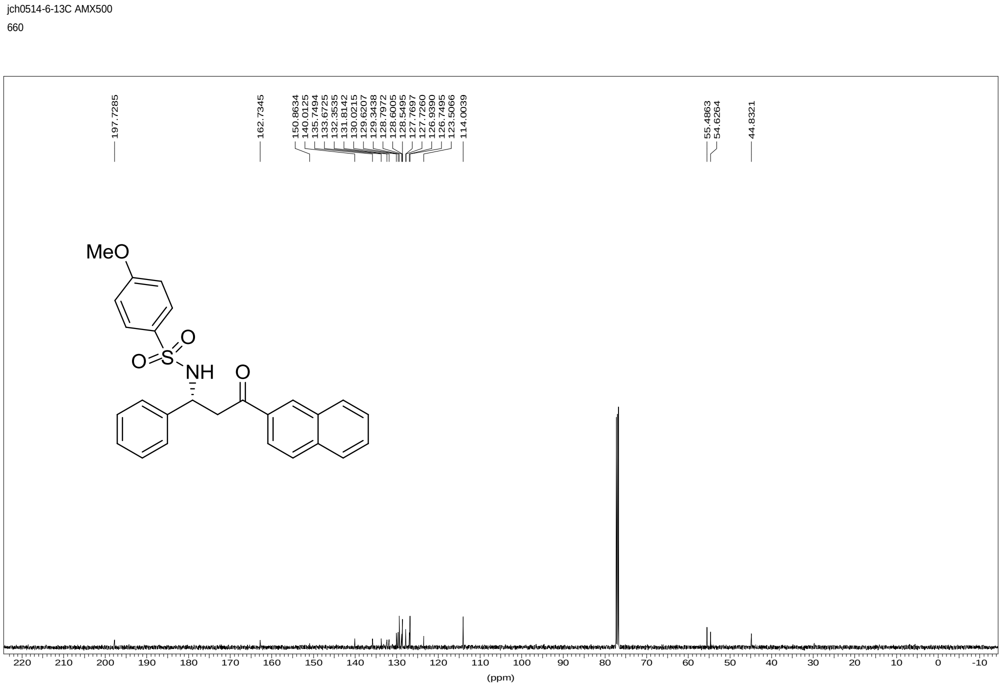

^{1}H and ^{13}C NMR spectra of **3u**

jch0514-7-1H AMX500
661

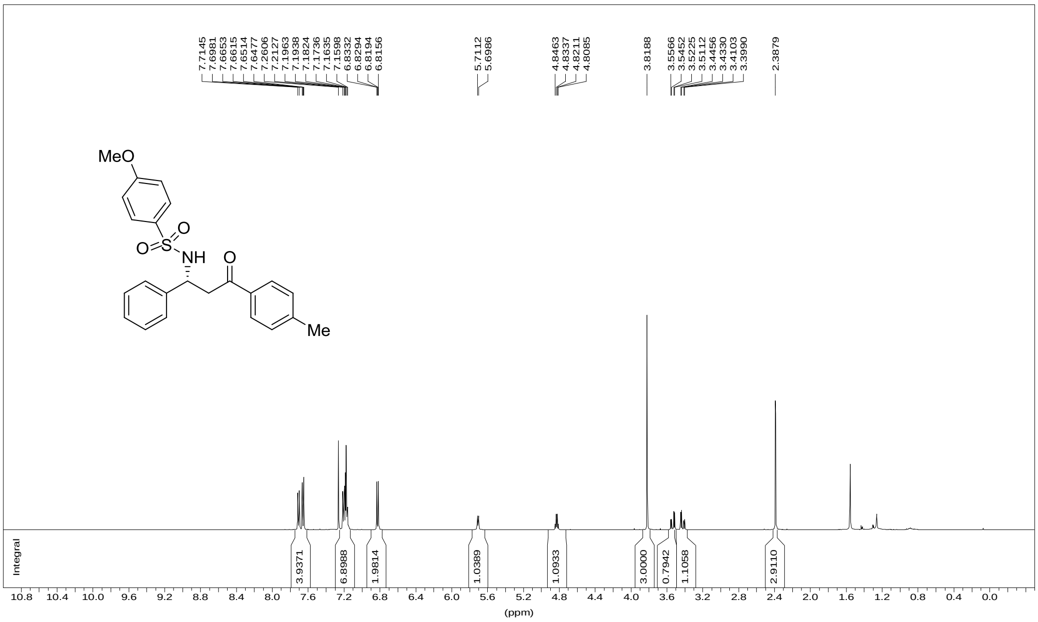

jch0514-8-13C AMX500
661

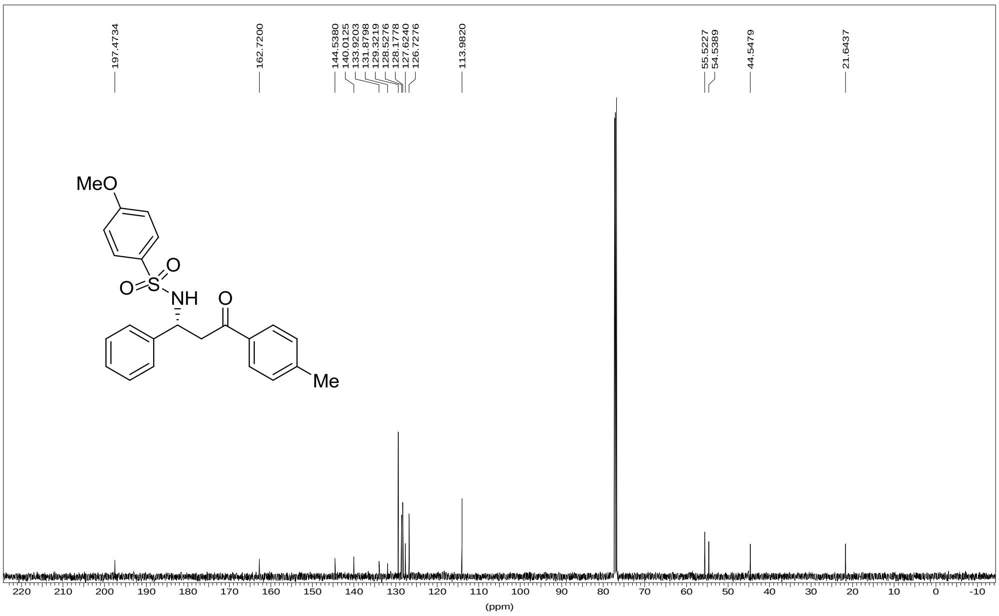

^{1}H and ^{13}C NMR spectra of **3v**

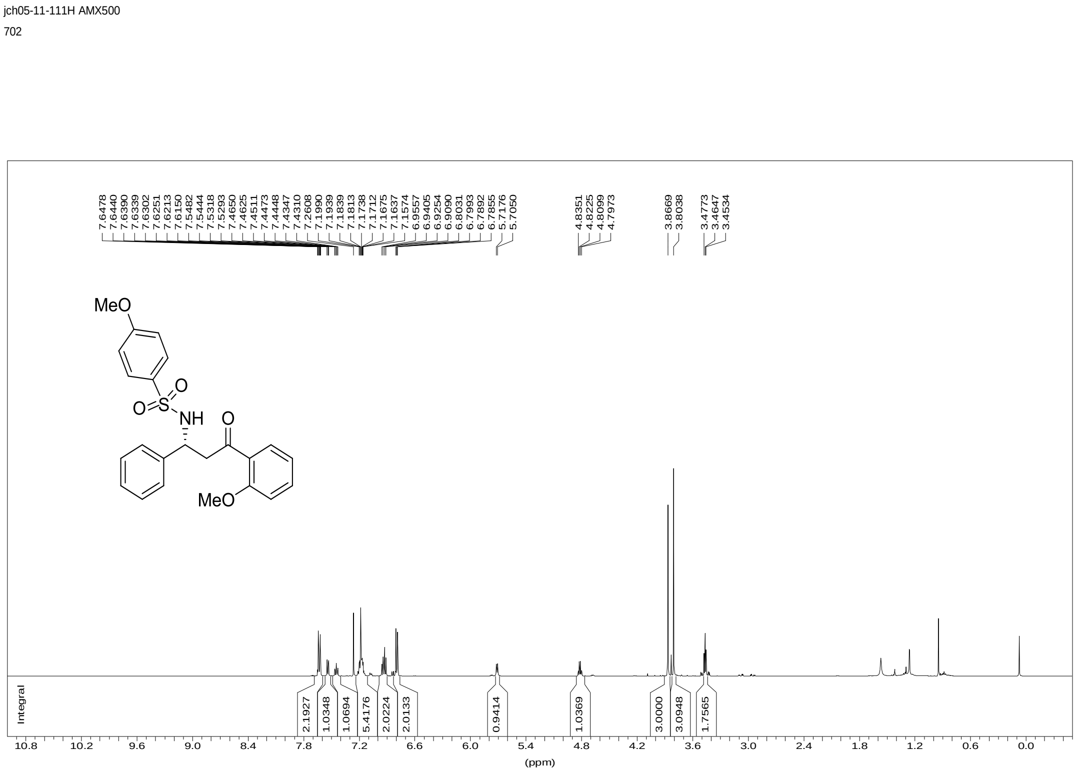

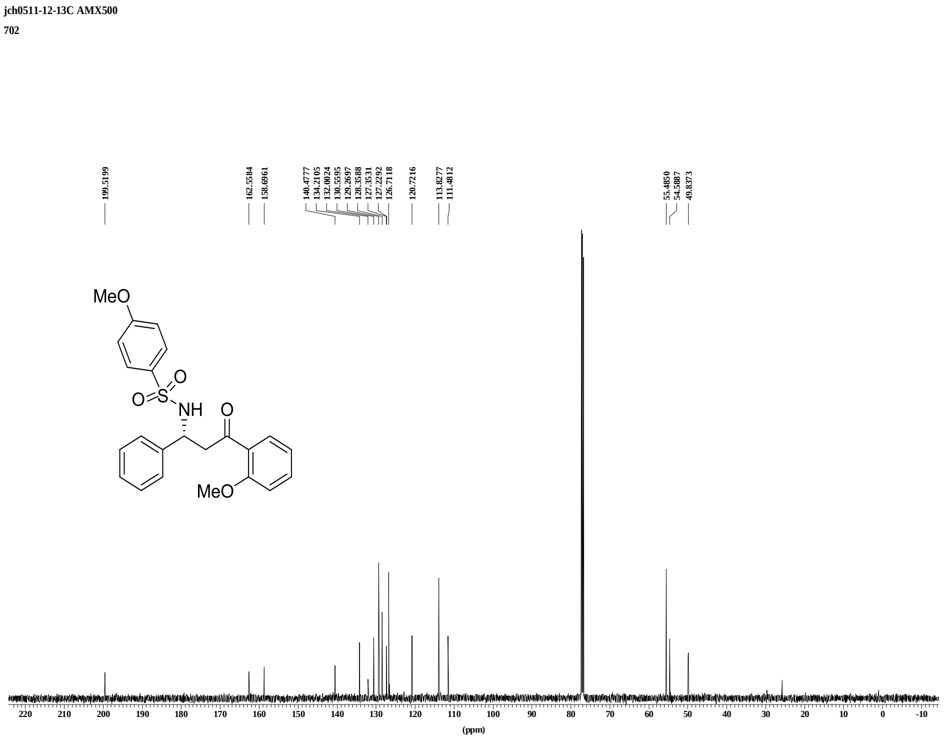

^{1}H and ^{13}C NMR spectra of **3w**

jch0511-71H AMX500
700

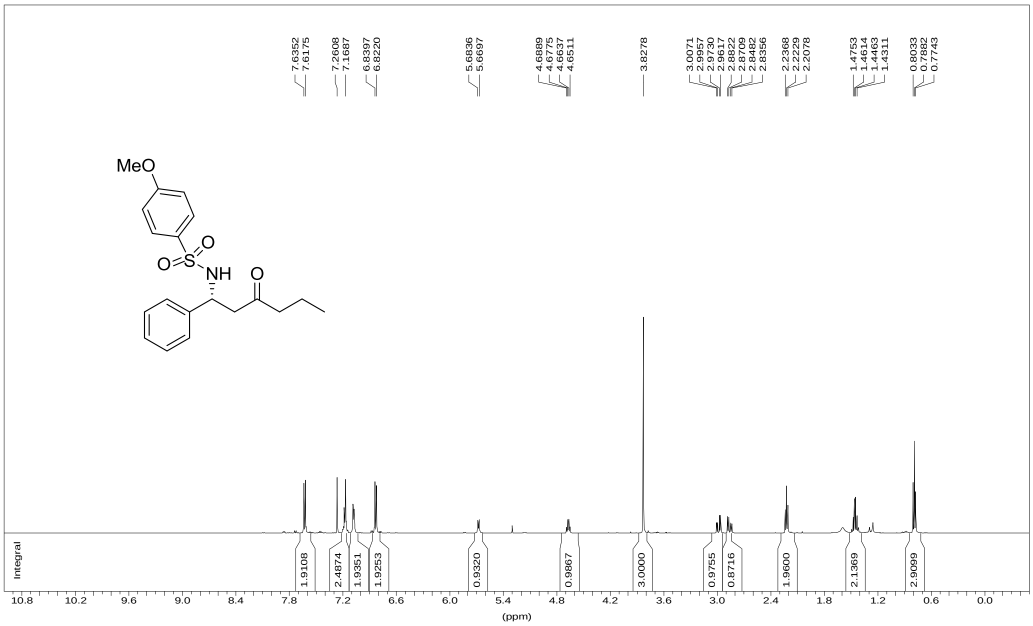

jch0511-813C AMX500
700

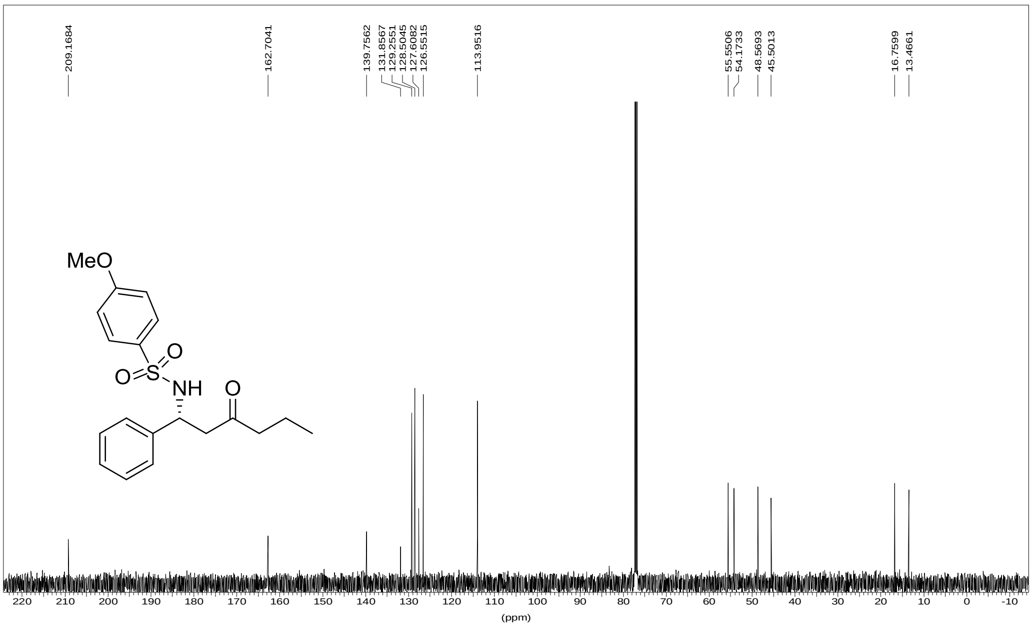

<u>^{1}H and ^{13}C NMR spectra of **3x**</u>

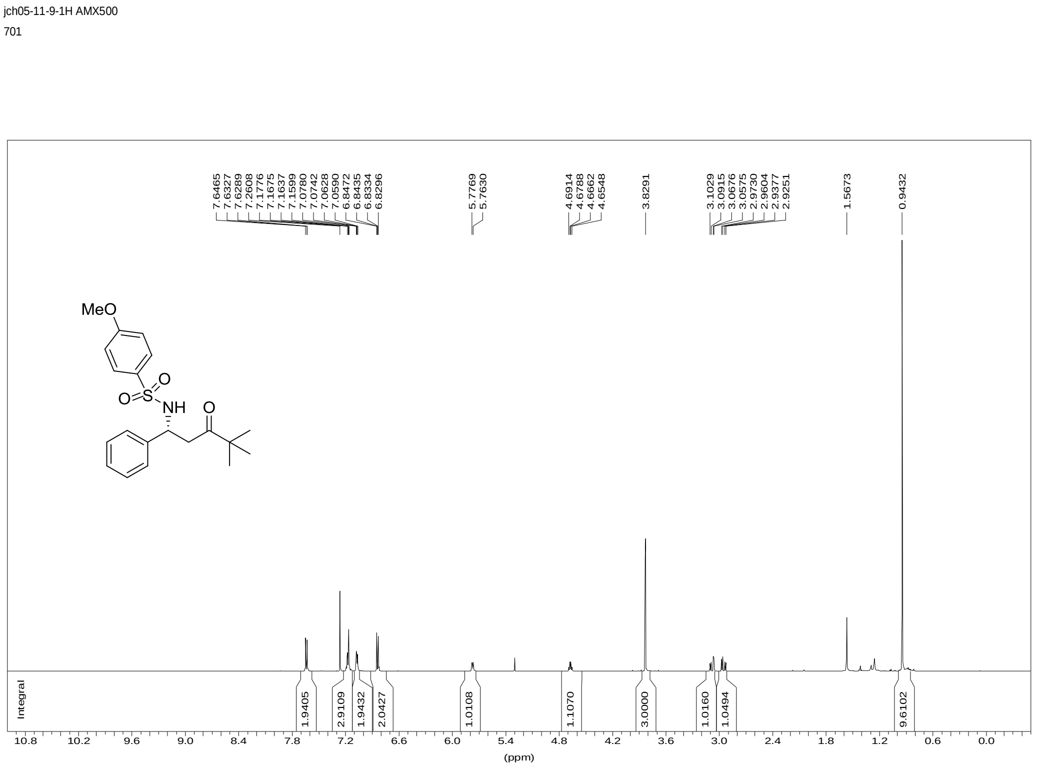

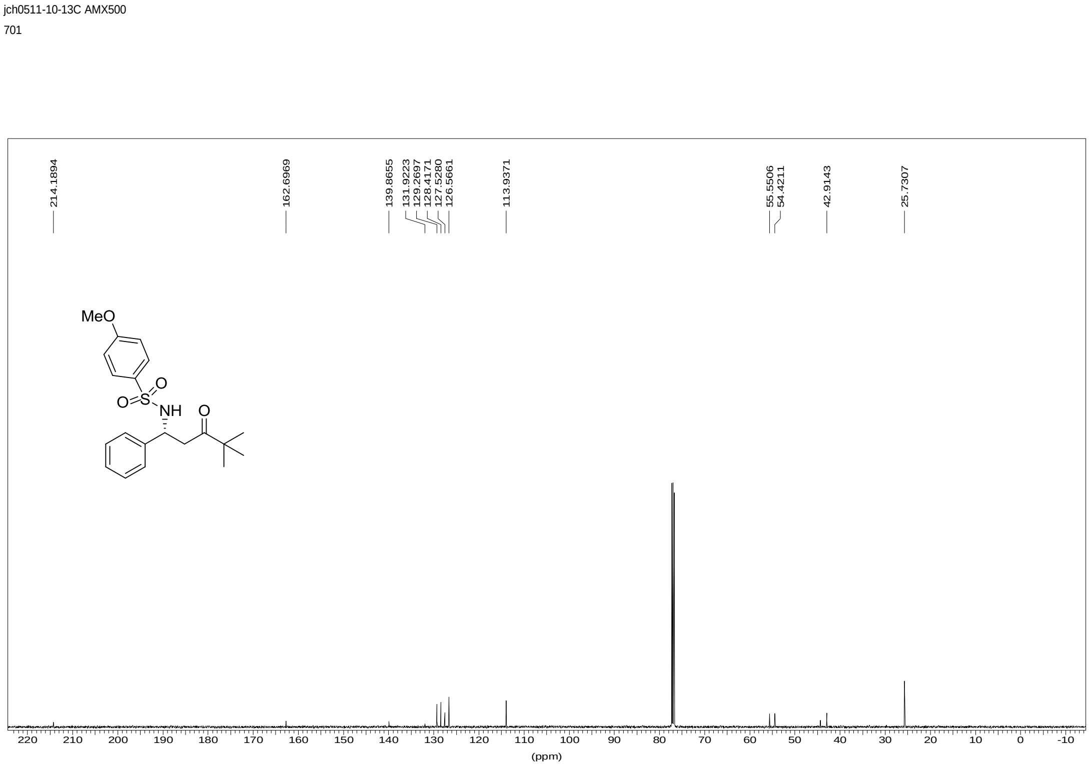

Supplement: File 1 — Characterization data and spectra of synthesized compounds. [file Beilstein_J_Org_Chem-08-1279-s001.pdf]
